# Genome-Wide Identification of Brassinosteroid Signaling Downstream Genes in Nine Rosaceae Species and Analyses of Their Roles in Stem Growth and Stress Response in Apple

**DOI:** 10.3389/fgene.2021.640271

**Published:** 2021-03-18

**Authors:** Liwei Zheng, Yingli Yang, Shengjie Ma, Wenming Wang, Jimeng Zhang, Caipeng Yue, Yongmei Wang, Maoping Song, Xinqi Hao, Jinyong Huang

**Affiliations:** ^1^School of Agricultural Sciences, Zhengzhou University, Zhengzhou, China; ^2^National Tobacco Quality Supervision and Inspection Center, Zhengzhou, China; ^3^First Affiliated Hospital of Zhengzhou University, Zhengzhou, China; ^4^College of Chemistry, Zhengzhou University, Zhengzhou, China

**Keywords:** brassinosteroid signaling downstream genes, Rosaceae, stress responses, apple, stem growth

## Abstract

Brassinosteroid signaling downstream genes regulate many important agronomic traits in rice. However, information on such genes is limited in *Arabidopsis* and Rosaceae species. We identified these genes in *Arabidopsis* and nine Rosaceae species. They were, respectively, named based on chromosomal locations. Segmental duplication and whole-genome duplication under purifying selection, as determined by Ka/Ks analysis, likely contributed to Rosaceae gene expansion. Apple (*Malus domestica*), *Arabidopsis*, and rice genes were generally similar, while several Rosaceae genes differed from their rice homologs in various characteristics, such as gene length, subcellular localization, transmembrane topology, conserved domains, secondary structures, and responses to external signals. The brassinosteroid downstream genes in apple were, respectively, induced or repressed by five phytohormones. Furthermore, these apple downstream genes were differentially expressed in different apple grafting combinations (“Nagafu No. 2”/“Malling 9” and “Nagafu No. 2”/“Nagafu No. 2”) and long–short shoot varieties (“Yanfu No. 6” and “Nagafu No. 2”). Responses of the *MdBZR* genes to diverse stress signals were examined and candidate hub genes were identified. These findings indicated that several brassinosteroid signaling downstream genes in Rosaceae functionally differed from their rice homologs, and certain apple genes may play roles in plant height and stress responses. This study provided valuable information and presented enriched biological theories on brassinosteroid signaling downstream genes in apple. Identification of such genes serve to help expand apple breeding and growth. This study provides useful information for brassinosteroid signaling downstream genes.

## Introduction

Previous studies report that brassinosteroid (BR) is involved in plant growth and development as well as stress responses (Singh and Savaldi-Goldstein, 2015; Zheng et al., 2018). Both BR biosynthesis and signaling genes have been systematically studied in model plants [e.g., *Arabidopsis thaliana* and rice (*Oryza sativa*)] (Ohnishi et al., 2006; Ma et al., 2014). Certain BR metabolic mechanisms, including hydroxylation, glycosylation, and reduction of active BR, protect against unexpected endogenous BR increases or decreases and have been clarified in species such as *A. thaliana* (Poppenberger et al., 2003). In apple, primary BR biosynthesis and metabolism genes, including *DWF1/4*, *CPD*, *CYP90D1*, *DET2*, *ROT3*, *BR6ox1/2*, *BAS1*, *UGT73C5*, and *BEN1*, have been identified (Zheng et al., 2018). The functions of BR signaling components [BR receptors, BRI1 and BAK1, BSK3, BSU1, BIN2 kinase, transcription factors (TFs), BZR1] have also been reported in plants (Wang et al., 2012; Vriet et al., 2013). The downstream functional mechanisms of BR have garnered increasing interest (Tong and Chu, 2018). Various BR downstream genes, including *BZR1-2*, *DLT*, *LIC*, atypical *bHLH TF (ILI1)*, *KN1-*type homeobox gene (*OSH1*), *RAV6*, *RAV1-like 1* (*RAVL1)*, *SMOS1*, *CSA*, *SPY*, and *GAST* gene *GSR1*, have also been identified in *O. sativa* (Zhang et al., 2012; Hirano et al., 2017; Qiao et al., 2017). *Arabidopsis* lacks some agronomic traits (leaf angle, grain size, and tillering) identified in rice, and roles of BR downstream genes in regulating these traits are limited.

Key BR signaling downstream genes and their activities have been widely reported in rice (Supplementary Table S1). The activity of *BZR1-2* is regulated by BR signaling, and *BZR1-2* can affect the transcriptional levels of genes involved in BR biosynthesis (Yin et al., 2005; Salazar-Henao et al., 2016; Wang et al., 2019). *BZR1-2* is involved in stem elongation, cell elongation through cell wall modification, transportation of ions and water, cytoskeletal rearrangement, and gibberellin (GA) biosynthesis and signaling (Xie et al., 2011; Tong et al., 2014). *BZR1-2* also participates in biological and abiotic stress responses. In rice, abscisic acid (ABA)-induced drought resistance, senility delay, and salt resistance are mediated by *BZR1*, *ABA INSENSITIVE 5* (*ABI5*), and *ABI3*, respectively (Lopez-Molina et al., 2002; Yang et al., 2016). Furthermore, jasmonic acid methyl ester (MeJA) enhances plant resistance to insects through the *BZR1* gene (Miyaji et al., 2014). Pathogen-associated molecular pattern (PAMP) receptors promote phosphorylation of BZR2, which controls plant immune responses to pathogens (Kang et al., 2015). *DLT* encodes a plant-specific GRAS (Tong et al., 2009) and participates in BR negative feedback regulation and GA metabolism in rice (Li et al., 2010). As a CCCH-type zinc finger gene, *LIC* is induced by BR and regulates tiller angles, plant height, and production in rice (Wang et al., 2008). Acting downstream of *BZR1*, *ILI1* controls shoot elongation and is up-regulated by BR (Zhang et al., 2009). The OSH1 protein with a KNOX domain plays an indispensable role in cytokinin (CK)-mediated shoot apical meristem (SAM) maintenance (Sentoku et al., 1999). Moreover, several BR catabolic genes are under the control of OSH1 during cell differentiation in SAM (Tsuda et al., 2014). BR treatment down-regulates *AtRAV1*, influencing lateral root and leaf growth (Hu et al., 2004). The RAV6 protein participates in BR homeostasis and affects plant architecture (Zhang et al., 2015). *SMOS1* (also named *RLA1*) can form a protein complex with BZR1 in regulating BR signaling and rice architecture (Qiao et al., 2017). *OsCSA* is a direct target of OsBZR1 and influences rice reproduction and yield (Zhu et al., 2015). OsSPY, an O-linked N-acetylglucosamine transferase, controls BR synthesis and GA signaling by interacting with the DELLA protein (Shimada et al., 2006; Wang et al., 2009). *OsGSR1* is involved in crosstalk between GA and BR (Shimada et al., 2006; Wang et al., 2009).

In summary, BR signaling downstream genes in rice share various functions in regulating important agronomic traits, such as plant height, tillering, yield, and environmental adaptations. Rosaceae plants, as important economic crops, exhibit many similar agronomic traits to rice. To date, however, limited information on the BR signaling downstream mechanisms in Rosaceae is available. Previously published Rosaceae genomes (e.g., *Malus domestica*, *Fragaria vesca*, *Pyrus communis*, *Prunus persica*, *Prunus avium*, *Prunus dulcis*, *Prunus mume*, *Rubus occidentalis*, and *Rosa chinensis*) provide useful tools for genome-wide searching of BR downstream genes. Here, we systematically identified BR signaling downstream genes in the above Rosaceae genomes. In total, 196 BR signaling downstream genes were characterized in this study. We analyzed their transmembrane helices, chromosomal locations, conserved domains, evolutionary relationships, gene/protein structures, synteny, and Ka/Ks ratios. Apple, as an important Rosaceae species, occupies a decisive position in the global fruit market in terms of cultivation area and yield. As a perennial woody fruit tree, the apple tree exhibits complex growth performance mediated by multiple hormones. For example, ABA, MeJA, and salicylic acid (SA) play essential roles in stress adaptation in apples (Li et al., 2015). Therefore, it is vital to identify apple BR signaling downstream genes and their functions. Promoter and protein–protein interaction analyses of the apple BR signaling downstream genes were performed, and their expression profiles were analyzed in various tissues. In addition, their responses to BR, brassinazole (BRZ), auxin (IAA), GA, and CK were explored using quantitative reverse transcription polymerase chain reaction (qRT-PCR). Their expression patterns in different shoot varieties [“Yanfu No. 6” (YF) and “Nagafu No. 2” (CF)] and grafting combinations [CF/“Malling 9” (M9) and CF/CF] were confirmed. In addition, the expression profiles of *MdBZR* genes in response to stress-related signals (ABA, MeJA, SA, drought, and salt) were identified. To provide a foundation for future studies on *MdBZR* genes, we cloned several candidate genes, i.e., *MdBZR1*, *MdBZR4*, *MdBZR6*, and *MdBZR7*, and identified the subcellular localization of *MdBZR1*. The data presented herein not only serve as a valuable resource for elucidating the functions of BR signaling downstream genes in nine Rosaceae species but also provide insights into their potential functions in apple.

## Materials and Methods

### Identification, Chromosomal Location, Gene Duplication, Collinearity, and Ka/Ks Ratio Analyses of BR Signaling Downstream Genes in Nine Rosaceae Species

The amino acid sequences of rice BR signaling downstream genes (*OsBZR1*, *OsBZR2*, *OsDLT*, *OsLIC*, *OsILI1*, *OsOSH1*, *OsRAVL1/OsRAV6*, *OsSMOS1*, *OsCSA*, *OsSPY*, and *OsGSR1*) were used to blast the *Arabidopsis* genome^1^ and nine Rosaceae databases using Blast v2.7.1 + software. Their distinctive domains were confirmed using pfam^2^, SMART^3^, and sequence alignment analysis. Protein sequences lacking corresponding characteristic domains were discarded from further analyses. Chromosomal locations were completed using the “gene location visualize” tool in TBtools (Chen et al., 2020). Genes were determined as duplicates in each of the genomes based on the following: (1) The aligned gene sequences were more than 70% identical, and the length of matching sequences was at least 70% of the longer gene (Yang et al., 2008). (2) On the same chromosome, duplicated genes separated by less than five genes within a 100 kb region were considered as tandem duplicates (Wang et al., 2010). (3) Duplicates on different chromosomes were characterized as segmental duplications (Wei et al., 2007). Collinearity analysis and visualization were completed using the “one step MCScanX” and “Amazing Super Circos” tools in TBtools (Chen et al., 2020). The Ka/Ks ratio was calculated using the “simple Ka/Ks calculator” tool in TBtools (Chen et al., 2020).

### Analyses of Phylogenetic Trees, Protein–Protein Interactions, Promoters, Gene/Protein Structures, Transmembrane Helices, Chemical Characters, and Sequence Alignments

The maximum-likelihood approach in MEGA X was used to construct a phylogenetic tree, and protein sequences were aligned using the ClustalW program with default parameters. STRING^4^ (option value >0.800) was used to construct an apple protein–protein interaction network using rice homologous proteins. In the PlantCARE database, *cis*-elements were identified in promoter sequences (2 kb upstream of the transcriptional start site). Gene structures were identified using coding sequences and full-length gene sequences with the “gene structure view” tool in TBtools (Chen et al., 2020). PHYRE (v2.0)^5^ and SOMPA^6^ were used to analyze protein secondary structures, and transmembrane helices were identified in TMHMM (v2.0)^7^. Chemical characters, including peptide length, molecular weight, isoelectric point (pI), amino acid composition, instability index, aliphatic index, and grand average of hydropathicity (GRAVY), were characterized using the ExPASy program^8^. DNAMAN v6.0 was used to align amino acid sequences.

### Plant Materials and Treatments

Shoot tips (STs), young stem xylem (YX), young stem phloem (YP), mature stem xylem (MX), mature stem phloem (MP), young leaves (YLs), mature leaves (MLs), and new roots (Rs) of 1 year-old M9 (apple) nursery trees were sampled (Zheng et al., 2019). The STs were collected at 0, 30, 60, 90, and 120 min after 3 mg/L BR and 0.5 mg/L BRZ (BR synthesis inhibitor) treatment, as per previous studies (Zheng et al., 2018, 2019).

STs were also collected at 0, 14, 28, 42, and 56 days after spraying with 3 mg/L BR (Zheng et al., 2018). We treated apple trees with 100 mg/L GA and 100 mg/L IAA, and sampled STs at 0, 14, 28, 42, and 56 days after treatment (Zheng et al., 2018). CK treatment (0.1 mg/L) on “Pingyitiancha” (*Malus hupehensis*) was performed in aerated half-strength Hoagland nutrient solution, as described previously, and STs were sampled at 0, 7, 14, 21, and 28 days (Zheng et al., 2018).

Both the YF (short-branched spur-type mutation) and CF (standard-type) varieties as well as CF/M9 (dwarf tree) and CF/CF (vigorous tree) grafting combinations were compared in experimental plots at the Yangling National Apple Improvement Center, Yangling, China (34.31°N, 108.04°E) (Song et al., 2017). STs from YF and CF were sampled at 65, 85, 105, 125, and 145 days after bud break (DABB); STs from CF/M9 and CF/CF were collected at 55, 80, 105, 130, and 155 DABB (Song et al., 2017). Stress-related treatments [300 μM ABA, 50 μM MeJA, 100 μM SA, drought, and salt (200 mM NaCl)] were carried out following a previous study (Chen et al., 2018). ABA-, MeJA-, and SA-treated YLs were sampled at 0, 3, 6, 12, and 24 h, and drought- and salt-treated YLs were also collected on days 0, 2, 4, 6, and 8.

The plant materials and treatments are summarized in Supplementary Table S2.

### Gene Expression Analysis, Molecular Cloning, and Subcellular Localization of Apple *MdBZR* Genes

RNA was isolated from the above apple samples (Gambino et al., 2008). A PrimeScript RT Reagent Kit with gDNA Eraser (Takara, Dalian, China) was used to synthesize first-strand cDNA. Primers of apple genes were designed using Primer 3 software (Supplementary Table S3). qRT-PCR analysis was completed using a SYBR Green qPCR Kit (TaKaRa, Dalian, China) on a Bio-Rad CFX 134 Connect Real-Time PCR Detection System (United States). Relative gene expression was calculated with the 2^–ΔΔCt^ method (Livak and Schmittgen, 2001). Target genes were amplified using Phanta HS Super-Fidelity DNA Polymerase (Vazyme, Nanjing, China). After their introduction into the pMD-18T vector (Takara), target genes were sequenced. The coding sequence of the *MdBZR1* gene without a termination codon was cloned in pCAMBIA1302 vector^9^ and then transferred into tobacco (*Nicotiana benthamiana*) leaves by the *Agrobacterium tumefaciens*-mediated method (Yoo et al., 2007). The transgenic tobacco plants were observed using confocal microscopy (TCS SP8, Leica, Germany).

### Statistical Analysis

The qRT-PCR was completed using three biological replicates for each sample, and three technical replicates for each reaction. *EF-1*α (GenBank accession no. DQ341381) served as the internal standard. Standard values, standard errors, and significance analyses of experimental data were subjected to an analysis of variance (ANOVA) at the 0.05 and 0.01 level with OriginPro 9.0 software (OriginLab Inc., Northampton, Massachusetts, United States).

## Results

### Characterization, Synteny, and Gene Structure Analyses of Genes Encoding BR Signaling Downstream Components in Rosaceae Species

**FIGURE 1:**
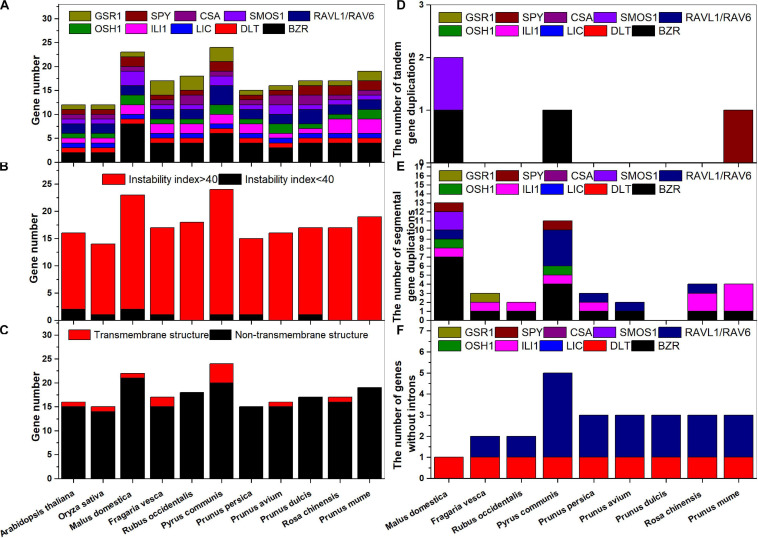
Numbers of BR downstream genes for each classification in Rosaceae species. **(A)** Each species; **(B)** instability index; **(C)** transmembrane structure; **(D)** tandem gene duplications; **(E)** segmental gene duplications; **(F)** genes without introns.

A genome-wide analysis of key BR signaling downstream genes (*BZR1-2*, *DLT*, *LIC*, *ILI1*, *OSH1*, *RAVL1*, *RAV6*, *SMOS1*, *CSA*, *SPY*, and *GSR1*) was completed for the nine Rosaceae species. We named them according to their chromosomal or scaffold positions (Supplementary Figure S1). A total of 196 BR signaling downstream genes were identified: 15–23 from nine Rosaceae species (Figure 1A), including 3–8 *BZR1*/*2s* and 1–3 other genes. All proteins, except for several SPYs, AtGSR1, and FvGSR1-3, shared instability indices above 40 (Figure 1B and Supplementary Table S4) and were unstable according to the published criteria (Feng et al., 2019). Most proteins did not contain transmembrane structures, except for MdBZR8, PcBZR3, RcBZR4, and so on (Figure 1C and Supplementary Figure S2).

Tandem gene duplications were identified in *M. domestica* (*MdBZR7*_*MdBZR8* and *MdSMOS1-1*_*MdSMOS1-2*), *P. communis* (*PcBZR2*_*PcBZR3*), and *P. mume* (*PmSPY1*_*PmSPY2*) (Figure 1D and Supplementary Figure S3). A total of 42 pairs of segmental duplications of BR signaling downstream genes were found in the nine Rosaceae species (Figure 1E). There were 0–13 segmental gene pairs in the nine Rosaceae species, respectively (Figure 1E and Supplementary Figure S3). One to seven segmental *BZR* gene pairs and 1–3 segmental *ILI1* gene pairs were identified in all Rosaceae species, except for *P. avium* and *P. dulcis*. Segmental *OSH1s* were only identified in *M. domestica* and *P. communis* (Supplementary Figures S3-1, 3-4), and 1–4 segmental *RAVL1/RAV6* genes were detected in *M. domestica*, *P. communis*, *P. persica*, *P. avium*, and *R. chinensis* (Supplementary Figures S3-1, 3-4, 3-5, 3-6, 3-8). Both *M. domestica* and *P. communis* had one segmental *SPY* gene pair (Supplementary Figures S3-1, 3-4); in addition, one pair of segmental *FvGSR1* genes was found (Supplementary Figure S3-2). The BR signaling downstream genes are well studied in rice (Tong and Chu, 2018). To identify their functions in Rosaceae, we compared syntenic genes in each of the Rosaceae and rice genomes, and some Rosaceae and rice genes were in the same pairs (Supplementary Figure S4). For example, *OsBZR2*_*MdBZR2*, *OsBZR2*_*MdBZR4*, and *OsBZR1*_*MdBZR6* were characterized as gene pairs (Supplementary Figure S4-1); two pairs were identified in *F. vesca* and rice (Supplementary Figure S4-2); two syntenic BR signaling downstream orthologous gene pairs were detected in *R. occidentalis* and *O. sativa* (Supplementary Figure S4-3); and other orthologous gene pairs are displayed in Supplementary Figures S4-4–S4-9. To identify selective pressure on gene pairs, Ka, Ks, and Ka/Ks values were calculated. The Ka/Ks values of all gene pairs were lower than 1 (Supplementary Tables S5, S4). However, some gene pairs, i.e., *PcRAVL1-1*_*PcRAV6-1*/*PcRAVL1-2*_*PcRAV6-2*, *PcILI1-1*/*PcILI1-2*, *PmILI1-2*/*PmILI1-3*, *OsOSH1*/*PmOSH1-1*, and so on, showed no non-synonymous mutations based on their Ks values (Supplementary Tables S5, S4).

All BR signaling downstream genes, except for certain *RAVL1/RAV6* and *DLT* genes, featured several introns (Figure 1F and Supplementary Figure S5). In general, the homologous genes shared similar gene structures (Supplementary Figure S5). For example, *BZRs*, *RAVL1s/FvRAV6s*, *CSAs*, *LICs*, *DLTs*, *GSR1s*, *ILI1s*, *SMOS1s*, *OSH1s*, and *SPYs* contained 1–17 exons, respectively. Detailed information on the BR signaling downstream genes in *Arabidopsis*, rice, and Rosaceae species is shown in Supplementary Table S4. Their coding sequences (CDSs) ranged from 249 (*PaLIC*) to 7248 (*PcBZR3*) base pairs (bp). The deduced polypeptides of these proteins were 83–2,416 amino acids (aa) in length, with molecular weights ranging from 9.35 to 266.69 kDa. In addition, protein characteristics, including pI, amino acid composition, aliphatic index, GRAVY, and subcellular localization, were determined (Supplementary Table S4).

### Phylogenetic, Conserved Domain, and Secondary Structure Analyses of BR Signaling Downstream Proteins

To identify the characteristic domains of the BR signaling downstream proteins, multiple protein sequences were aligned (Supplementary Figure S6). In the N-terminus of most BZR1/2s, a nuclear localization signal (NLS) and atypical basic helix-loop-helix (bHLH) DNA-binding motif with basic, helix1, loop, and helix2 regions were detected (Supplementary Figure S6-1). However, PaBZR1, PcBZR1, and PcBZR2 shared no NLS or bHLH DNA-binding motif, and PaBZR1 lost the S-rich phosphorylation site. There were less consistent PEST and C-terminal domains among all BZRs, except for MdBZR8 and PcBZR4 (Supplementary Figure S6-1). The DLTs contained conserved leucine heptad I domain with IA and IB, VHIID motif, leucine heptad II with A and II B, PFYRE, and SAW domains (Supplementary Figure S6-2). In rice, *Arabidopsis*, and Rosaceae RAVL1-RAV6s, a conserved B3 DNA-binding domain consisting of ∼90 aa residues was uncovered in the central part (Supplementary Figure S6-3). At the N-terminus, LIC proteins contained a single C-x8-C-x5-C-x3-H (CCCH)-type domain. A conserved EELR domain was found in the middle part of the LICs and a less conserved serine-rich region was located at the C-terminus. However, PaLIC lacked the EELR and serine rich domains (Supplementary Figure S6-4). All OSH proteins contained a KNOX domain at their N-terminus, and there was a conserved ELK domain, including one conserved basic end and three conserved helix motifs at the C-terminus (Supplementary Figure S6-5). As AP2 TFs, the SMOS1 proteins contained five conserved domains, including AKER, AP2 (containing highly constant AP2-R1, linker region, and AP2-R2 motifs), EPY, ILS, and C-terminus WTNF domains; however, the EPY, ILS, and WTNF domains were not identified in PaSMOS1-2 (Supplementary Figure S6-6). Highly conserved R2 and R3 domains were detected in the CSA proteins (Supplementary Figure S6-7). There was a C-terminal region containing C, R, G, K, and Y residues in the GSR1 proteins (Supplementary Figure S6-8). Long conserved TRP, CD I, and CD II domains were identified in SPY proteins (Supplementary Figure S6-9). Two helix regions and one small loop region were identified in the ILI1 proteins (Supplementary Figure S6-10).

These protein secondary structures are shown in Supplementary Figure S7 and Supplementary Table S7. Most proteins contained an α helix, extended strand, random coil, and β-turn, and a mass of α helices or random coils were found in all proteins. Random coils and β-turns were, respectively, the most and least abundant structures in the BZR proteins, except for PmBZR3. In space, α helices occupied the main positions in most BZR proteins, except for AtBZR1, OsBZR2, PcBZR4, PaBZR1, RcBZR1-2, RcBZR4, and PmBZ1 (Supplementary Figure S7-1 and Supplementary Table S7). Among the DLTs, α helices and random coils accounted for more than 80% of all secondary structures, and α helical structures took up considerable space (Supplementary Figure S7-2 and Supplementary Table S7). The LIC proteins contained more than 70% random coils and less than 5% β-turns, with the proteins predominately made up of random coils in space (Supplementary Figure S7-3 and Supplementary Table S7). The ILI1, OSH1, and GSR1 proteins, which shared a few extended strands and β-turns, mainly consisted of α helices or random coils in space (Supplementary Figures S7-4, S7-5, S7-10, and Supplementary Table S7). Many random coils were found in RAVL1s/RAV6s, SMOS1s, and CSAs (Supplementary Figures S7-6–S7-8 and Supplementary Table S7). For SPYs, α helices were the most abundant structure (Supplementary Figure S7-9 and Supplementary Table S7).

**FIGURE 2:**
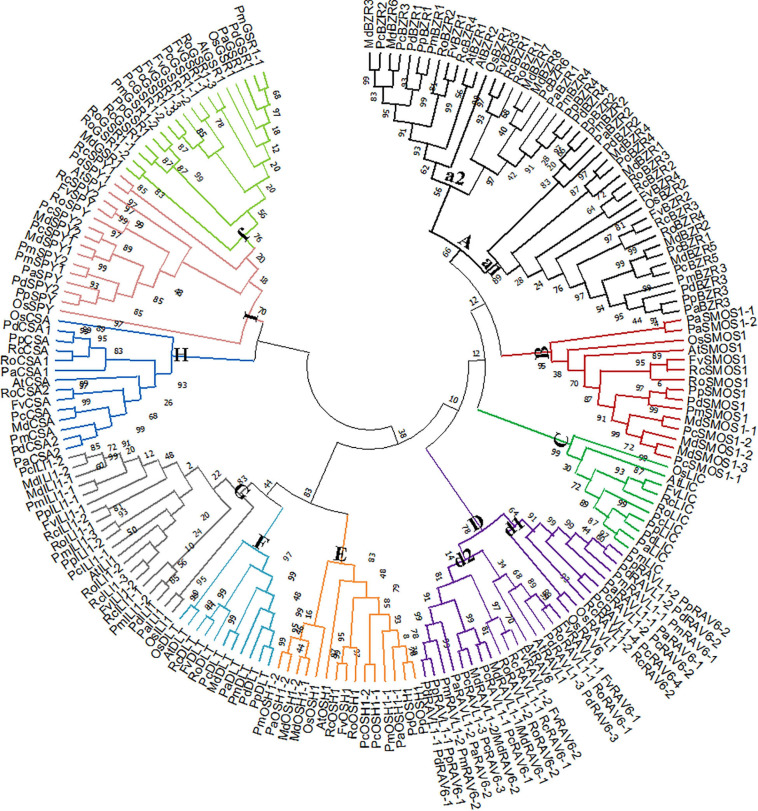
Phylogenetic analysis of rice, *Arabidopsis*, and Rosaceae BR downstream genes.

We next constructed a phylogenetic tree. As expected, Rosaceae BR signaling downstream genes were highly homologous to their corresponding rice and *A. thaliana* homologs (Figure 2). In group A, *BZRs* were divided into two subgroups. *OsBZR1*, *AtBZR1-2*, and *MdBZR3* were clustered in subgroup a2, with other genes located in subgroup a1. The *P. avium SMOS1* genes showed relatively distant phylogenetic relationships with other *SMOS1s* in group B. Rosaceae *LIC* genes showed a close evolutionary relationship in group C, and *OsLIC* and *AtLIC* were closely clustered together. Most *RAV* genes were located in subclass d1, although some were also identified in subclass d2. In group E, *OsOSH1* separated with other *OSH1* genes. *AtDLT*_*OsDLT* and Rosaceae *DLT* genes were closely clustered in group F. Class G was divided into subgroup g1, composed of *FvILI1-2*, *RoILI1-2*, *RoILI1-3*, *AtILI1*, *PcILI1-1*, *PpILI1-2*, and *PmILI1-3*, and subgroup g2, composed of the other *ILI1s*. All *CSA* genes clustered together in group H, while *PaCSA1* shared a distant relationship with other *CSA1* genes. All *SPYs* were clustered in subclass I, whereas *OsSPY* was relatively independent. *MdGSR1* and *RoGSR1-2* closely clustered together in group J.

### Promoters and Protein–Protein Interaction Analyses of Apple BR Signaling Downstream Genes

To further investigate apple BR signaling downstream genes, we examined the *cis*-acting elements in their promoters. We identified ABA-, MeJA-, GA-, IAA-, SA-, and BR-related motifs in these genes (Supplementary Figure S8). For example, ABA-response motifs (ABRE) and GA-related units (GARE, P-box, or TATC-box) were identified in all genes. In addition, MeJA-related elements (CGTCA- and TGACG-motifs), TGA element or AuxRR-core responding to auxin, SA-related SARE or TCA elements, BR-related elements (BRRE, G-box, E-box, or GATGTG), and stress-related elements (TC-rich repeats, MBS, and DRE) were found in most genes. In addition, elements (MSA-like or MBSI) referring to growth and development were found in *MdBZR1*, *MdBZR4*, *MdCSA*, *MdDLT*, and *MdOSH1-1.*

The STRING program was used to prepare a protein–protein interaction network. In the network, nine highly interactive proteins were encoded by the BR signaling downstream genes (Supplementary Figure S9). Other proteins [BR signaling shaggy-related protein kinase (GSK1/2), trigger factor-like protein OsJ_21100, OSH1, zinc metalloproteinase-like (OS02T0761400-01), kinesin-like protein KIN-13A (SRS3), protein GIGANTEA (GI), OS02T0499000-01, OS04T0195000-01, and OS01T0205700-01] were closely related to BR biosynthesis and signaling pathways. PRS, OsJ_22473, PRR1, HD2, OsJ_14537, OS04T0223000-01, OS08T0178100-01, OS03T0715500-02, and OS01T0666800-01 were also potential BR signaling downstream protein patterns. The components of the above network were analyzed using Gene Ontology (GO) and Kyoto Encyclopedia of Genes and Genomes (KEGG) (Supplementary Table S8). Most were enriched in BR-related signaling pathway (GO:0009742), transcription factor activity (GO:0006355, GO:0003677, GO:0003700, and GO:0005634), hormone-mediated terms (GO:0009755 and ath04075), growth and development-related terms (GO:0040008 and GO:0032502), and stress-related terms (GO:0048583).

### Expression Analysis of BR Signaling Downstream Genes in Different Tissues and Response to BR, BRZ, IAA, GA, and CK Treatments

**FIGURE 3:**
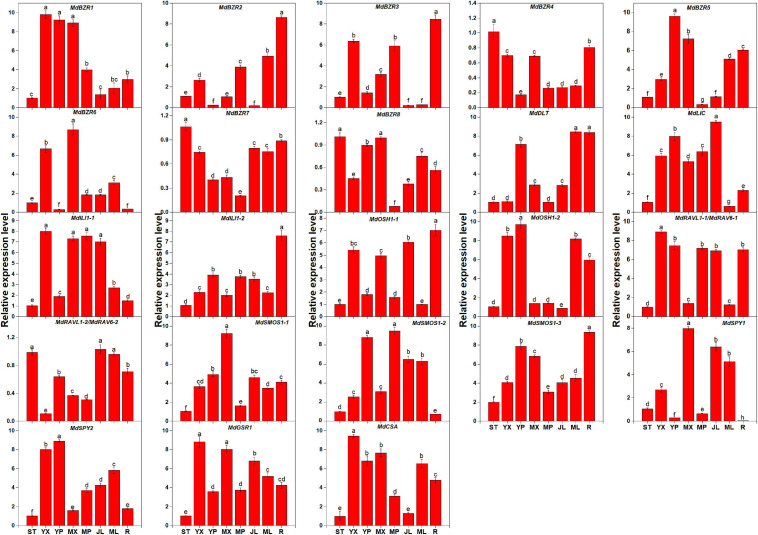
Expression profiles of apple-specific BR downstream genes in different tissues. Each value represents mean ± standard error of three biological replicates. Different lowercase letters indicate significant differences at 0.05 level.

The expression levels of BR signaling downstream genes in STs, YX, YP, MX, MP, YLs, MLs, and Rs were investigated (Figure 3). Results showed that the genes exhibited different expression patterns in the above tissues. In STs, *MdBZR4*, *MdBZR7*, and *MdBZR8* showed the highest levels. Twelve genes, such as *MdBZR1*, *MdBZR5-6*, *MdILI1-1*, and so on, were highly expressed in the stem tissues (YX, YP, MX, or MP). Nine genes (*MdBZR2*, *MdBZR3*, *MdBZR4*, and so on) were highly expressed in roots. *MdBZR4* and *MdBZR7-8* were found at the highest levels in STs. Other genes were mainly expressed in the YLs and MLs.

To detect the effects of BR on apple genes, their expression levels were assessed after BR and BRZ treatment in STs (Supplementary Figures S10, S11). Most *MdBZR* genes were up-regulated by BR, although some were down-regulated (Supplementary Figure S10). Moreover, several genes were induced or repressed at different time points (Supplementary Figure S10). For example, *MdBZR1* showed an 8-, 15-, and 2-fold increase at 30, 60, and 90 min after BR treatment, respectively. However, *MdBZR4* and *MdBZR8* were down-regulated at 30–120 min and *MdBZR5* was down-regulated at 60 and 90 min after BR treatment. The expression of *MdDLT* decreased from 60 to 120 min in response to BR treatment. Furthermore, BR induced a 2- to 20-fold increase in *MdLIC*, *MdILI1-1*, *MdSMOS1-3*, and *MdCSA*. *MdILI1-2* expression was obviously up-regulated by BR at 60, 90, and 120 min, but inhibited at 30 min. *MdRAVL1-1/MdRAV6-1*, *MdRAVL1-2/MdRAV6-2 MdSMOS1-1*, and *MdGSR1* were down-regulated after BR treatment. The expression levels of *MdSMOS1-2* were higher at 30 and 60 min than 0 min, but lower at other times (*p* < 0.05). The responses of the above genes to exogenously applied BRZ were also investigated (Supplementary Figure S11). Results showed that *MdBZR1*, *MdBZR2*, *MdBZR3, MdBZR6, MdBZR7, MdLIC*, *MdILI1-1*, *MdSMOS1-3*, and *MdCSA* were down-regulated by BRZ at most time points. BRZ up-regulated *MdBZR4, MdBZR5, MdBZR8*, *MdDLT*, and *MdGSR1* at three time points, respectively. However, *MdILI1-2* and *MdSMOS1-2* were unaffected by BRZ. *MdRAVL1-1/MdRAV6-1*, *MdRAVL1-2/MdRAV6-2*, and *MdSMOS1-1* showed a 1.5-−4.5-fold increase from 30 to 120 min.

**FIGURE 4:**
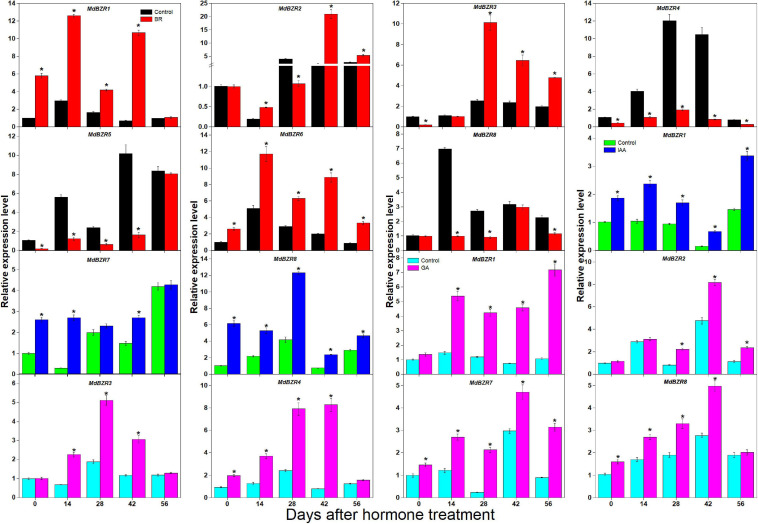
Effects of BR, IAA, and GA on *MdBZR* gene expression. qRT-PCR data are shown relative to day 0. Bars show mean ± standard error (*n* = 3). Asterisks indicate significant differences at 0.05 level (*).

**FIGURE 5:**
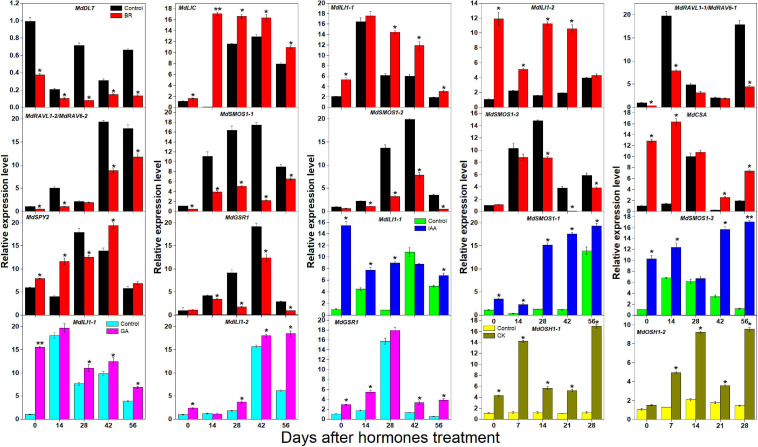
Effects of BR, IAA, and GA on apple BR downstream genes (except for *MdBZRs*). qRT-PCR data are shown relative to 0 day. Bars show mean ± standard error (*n* = 3). *, ** indicate significant differences at 0.05 and 0.01 level, respectively.

BR, IAA, GA, and CK are known to promote apple tree stem growth and development (Zheng et al., 2018). To identify the roles of BR signaling downstream genes in apple tree stem growth and development, their expression profiles were further investigated (Figures 4, 5). *MdBZR1*, *MdBZR2, MdBZR3*, and *MdBZR6* were induced by BR, generally. *MdBZR4*, *MdBZR5*, and *MdBZR8* were separately down-regulated in BR-treated STs. However, the expression pattern of *MdBZR7* was irregular in response to BR treatment (Supplementary Figure S12). *MdBZR1, MdBZR7* and *MdBZR8* were up-regulated by IAA, generally (Figure 4). The other *MdBZR* genes showed irregular expression patterns after IAA treatment (Supplementary Figure S12). GA up-regulated several *MdBZR* genes, including *MdBZR1-4* and *MdBZR7-8.* For example, *MdBZR1* showed a 2–6-fold increase in the GA-treated STs. From days 28 to 56, *MdBZR2* expression was higher (*p* < 0.05) in GA-treated samples than in the controls (Figure 4). GA exposure irregularly influenced *MdBZR5* and *MdBZR6* (Supplementary Figure S12). The expression patterns of other BR signaling downstream genes were also explored after hormone treatments (Figure 5). At most time points, the expression levels of *MdDLT*, *MdLIC, MdILI1-1*, and *MdILI1-2* decreased in the BR-treated plants (Figure 5). The expression levels of *MdRAVL1s/MdRAV6s*, *MdSMOS1s*, *MdGSR1*, and *MdCSA* decreased at 3–5 time points following BR exposure (Figure 5). *MdSPY2* showed higher (*p* < 0.05) expression levels in the treatment group than in the control on days 0, 14, and 42, but showed lower (*p* < 0.05) levels on day 28. *MdSPY1* was only induced by BR on day 0 (Supplementary Figure S12). *MdILI1-1*, *MdSMOS1-1*, and *MdSMOS1-3* were significantly up-regulated at most time points after IAA treatment (Figure 5), but transcription patterns of *MdILI1-2* and *MdSMOS1-2* were not influenced (Supplementary Figure S12). After GA treatment, *MdILI1-1* and *MdILI1-2* increased on days 0, 28, 42, and 56, and *MdGSR1* showed increased expression in GA-treated samples at all-time points, except for day 28 (Figure 5). Additionally, *MdOSH1-1* was up-regulated by CK at five time points and CK induced *MdOSH1-2* from days 7 to 28 (Figure 5).

### Expression Analysis of BR Signaling Downstream Genes in Two Branch Types and Grafting Combinations of Apple Trees

**FIGURE 6:**
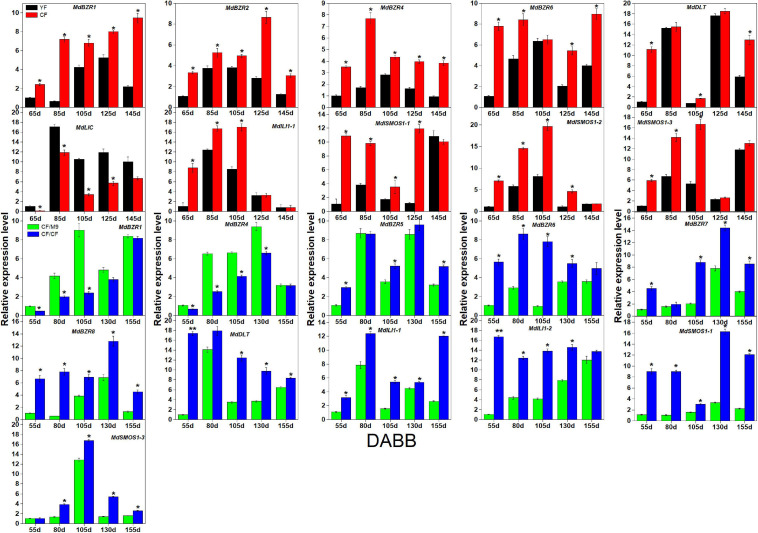
Expression analysis of apple BR downstream genes in YF-CF and CF/M9-CF/CF. DABB: days after bud break. Each value represents mean ± standard error of three biological replicates. Asterisks indicate significant differences at 0.05 level (*) and 0.01 level (**).

As several BR signaling downstream genes (*BZR1-2*, *DLT*, *LIC*, *ILI1*, and *SMOS1*) are involved in stem elongation (Li et al., 2010; Qiao et al., 2017), we analyzed their expression patterns in two branch-type apple trees (YF and CF) (Figure 6). YF exhibited a lower (*p* < 0.05) shoot elongation rate, internode number, and average internode length than CF (Song et al., 2017). *MdBZR1-2*, *MdBZR4, MdBZR6, MdDLT, MdILI1-1*, and *MdSMOS1s* showed higher expression levels in CF than in YF at most time points. The transcriptional levels of *MdLIC* were lower (*p* < 0.05) in CF than in YF at 65, 85, 105, and 125 DABB. Regarding the grafting combinations, CF/CF grows more vigorously than CF/M9 (Zheng et al., 2017). Several BR signaling downstream genes showed different expression patterns in CF/M9 and CF/CF (Figure 6). For example, *MdBZR1* and *MdBZR4* showed lower (*p* < 0.05) levels in CF/CF than in CF/M9 at 3–4 time points. *MdBZR5-8, MdDLT, MdILI1s*, and *MdSMOS1-1* were highly expressed in CF/CF trees at most time points. The expression patterns of the remaining *MdBZRs*, *MdDLT*, *MdLIC*, *MdILI1*, and *MdSMOS1* genes were irregular among YF-CF and CF/M9-CF/CF (Supplementary Figure S12).

### Effects of Stress-Related Treatment on *MdBZR* Gene Expression Profiles

**FIGURE 7:**
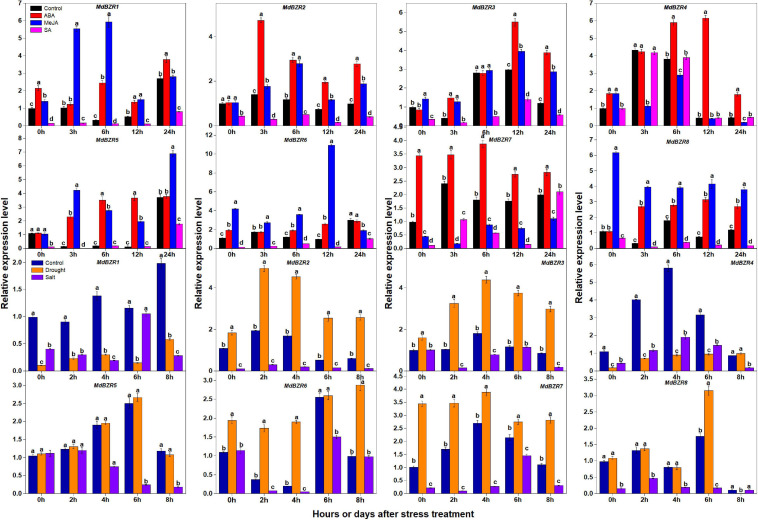
Expression levels of *MdBZRs* after hormone (ABA, MeJA, and SA) and stress (drought and salt) treatments. qRT-PCR data are shown relative to 0 h. Bars show mean ± standard error (*n* = 3). Different lowercase letters indicate significant differences at 0.05 level.

*BZR1* and *BZR2* play important roles in response to stress (Kang et al., 2015; Yang et al., 2016). We investigated the expression patterns of apple *BZR* genes after ABA, MeJA, SA, drought, and salt treatments in YLs (Figure 7). *MdBZR1-3*, *MdBZR5-6*, and *MdBZR8* were generally induced by ABA and MeJA but was down-regulated in SA-treated samples. In ABA-treated samples, *MdBZR4* was up-regulated ∼0. 9-, 0. 45-, 11-, and 2-fold at 0, 6, 12, and 24 h, respectively. In MeJA-treated leaves, the expression of *MdBZR4* was inhibited at 3, 6, and 24 h, but increased at 0 h. *MdBZR4* was not affected by SA exposure. At all-time points, the transcript levels of *MdBZR7* were higher (*p* < 0.05) in ABA-treated leaves than in the controls, but lower in MeJA-treated samples and repressed in SA-treated samples at *p* < 0.05 level.

The expression patterns of *MdBZR1-8* were also identified after drought and salt treatments (Figure 7). The transcript levels of *MdBZR1* were higher (*p* < 0.05) in the control group than in the drought- and salt-treated groups at most time points. In drought-treated leaves, *MdBZR2* and *MdBZR3* generally increased, but their expressions were down-regulated by salt. Drought and salt obviously repressed *MdBZR4* at most time points. The expression of *MdBZR5* was unaffected by drought but was down-regulated by salt on days 4, 6, and 8. The transcript levels of *MdBZR6* and *MdBZR7* in leaves were higher (*p* < 0.05) in the drought-treated group than in the control, whereas salt treatment decreased them. *MdBZR8* expression increased on day 6 but decreased on day 8 in the drought-treated group. In salt-treated trees, *MdBZR8* was low at most time points, except for day 8.

### Cloning and Subcellular Localization of Selected Apple *BZR* Genes

*MdBZR* genes (especially *MdBZR1*) may play important functions in both stem growth and abiotic stress responses. To further clarify their functions, *MdBZR1*, *MdBZR4*, *MdBZR6*, and *MdBZR7* were cloned. Compared with its reference sequence, the cloned *MdBZR1* was missing a 21 and 17 bp sequence at the 5′ end and 3′ end, respectively (Supplementary Figure S13A). There was a 36 bp insertion and one mismatch at the 5′end of the cloned *MdBZR4* compared to its reference sequence (Supplementary Figure S13B). There were sequence deletions and mismatches at the 3′end of the *MdBZR6* clone compared with its reference (Supplementary Figure S13C). Several sequence mismatches and a small insertion were identified in the *MdBZR7* clone (Supplementary Figure S13D). The cloned MdBZR1 and MdBZR4 proteins were shorter and longer, respectively, than their references (Supplementary Figures S14A,B). Like their nucleotide sequences, the protein sequences of cloned and reference *MdBZR6* shared variation at the C-terminal (Supplementary Figure S14C). There were only nine mismatches in the protein sequence between the cloned and reference MdBZR7 (Supplementary Figure S14D). The accuracies of the identified genes in this study were confirmed from the above findings.

**FIGURE 8:**
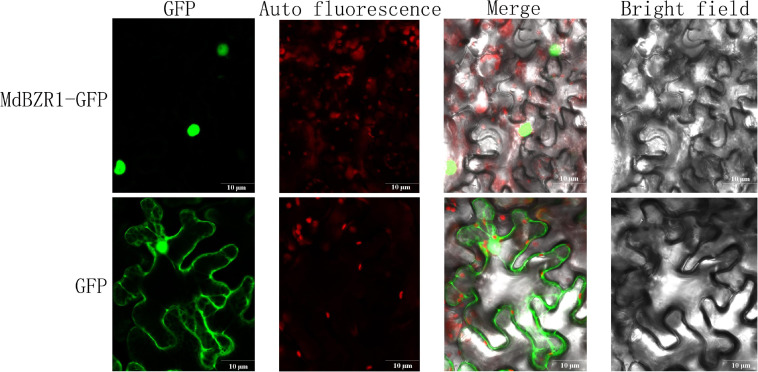
Subcellular localization analysis of *MdBZR1*. Confocal images of transiently transformed tobacco epidermal cells with green fluorescent protein (GFP) or *MdZR1*-GFP.

As TFs, *MdBZRs* were predicted to be located in the nucleus (Supplementary Table S4). To validate the results from bioinformatics analysis and lay a foundation for further identifying *MdBZR1’s* function, 35:*MdBZR1-GFP* and 35:*GFP* were transiently transformed into tobacco leaf. As a positive control, GFP fusion protein was targeted in both the nucleus and cytoplasm. We found that MdBZR1-GFP was characteristically located in the nucleus (Figure 8).

## Discussion

BR influences various important agronomic traits in plants, such as agricultural and environmental adaptations, through downstream components (Xie et al., 2011; Miyaji et al., 2014). The functions of BR downstream components have been revealed in rice, thus demonstrating the feasibility of acquiring desirable traits in the breeding of other crops. However, information regarding corresponding *Arabidopsis* and Rosaceae genes is not available. Here, *Arabidopsis* and nine Rosaceae BR downstream genes were characterized, with information on chromosomal localizations, transmembrane helices, physicochemical properties, synteny analyses, phylogenetic relationships, Ka/Ks ratios, gene/protein structures, promoter analyses, and protein–protein interactions further clarified. Moreover, their expression patterns in various tissues and responses to different growth-related factors as well as stress signals were investigated in apple. These findings should provide rich information on Rosaceae BR downstream genes and support the utilization of these genes to improve the agronomic traits of apple trees.

### Genome-Wide Identification and Characteristics Analysis of BR Downstream Genes

In rice, most BR downstream genes have been found with only one copy, with four *OsBZRs* and two *OsRAVL1/OsRAV6s* reported (Figure 1A). In the current study, we identified several additional BR downstream genes in Rosaceae species (Figure 1B). For example, we identified eight *MdBZR1*/*2*, two *MdILI1*, two *MdOSH1*, two *MdSPY*, and three *MdSMOS1* genes in apple. Six *PcBZR1s* or *PcBZR2s*, two *PcILI1s*, two *PcOSH1s*, two *PcSMOS1s*, four *PcRAVL1s*, or *PcRAV6s*, and three *PcGSR1s* were also identified. It is possible that gene duplications may result in gene expansions (Xu et al., 2012). Here, tandem and segmental duplications were found in almost all Rosaceae BR downstream genes (Figure 1E and Supplementary Figure S3). Whole-genome duplication (WGD) has also been reported in many Rosaceae species during their domestication (Ou et al., 2019; Feng et al., 2020). Therefore, BR downstream gene expansion may have resulted from gene duplication and WGD. In addition, the Ka/Ks values of most duplicated or homologous gene pairs in each Rosaceae species were < 1 (Supplementary Tables S5, S6), indicating that these genes likely expanded under purifying selection. Based on their instability indices, most BR downstream genes were unstable (Figure 1B). Introns play important roles in regulating gene expression (Wang et al., 2005). We found that one Rosaceae *DLT* or four Rosaceae *RAVL1/RAV6* did not contain introns (Figure 1F), potentially highlighting their unique expression patterns.

Orthologous genes in pairs were identified between rice and each Rosaceae species (Supplementary Figure S4). The functions of the Rosaceae BR signaling downstream genes may be predicted according to their corresponding rice genes. For example, *OsBZR1/2* participates in regulating culm length and lamina joint inclination (Bai et al., 2007), *OsGSR1* is involved in controlling BR biosynthesis and plant size (Wang et al., 2009), and *OsOSH1* maintains SAM, with *osh1* mutants being dwarfs (Tsuda et al., 2011). Most BR signaling downstream proteins contained similar characteristic domains, although PaBZR1, PcBZR1, PcBZR2, PaLIC, and PaSMOS1-2 proteins all lacked one domain each (Supplementary Figure S6), which may result in altered function. For example, PaBZR1, PcBZR1, and PcBZR2 proteins all lacked a bHLH DNA-binding motif, which may lead to a loss in their DNA binding capacity. PaBZR1 protein lacked a serine-rich phosphorylation site (Supplementary Figure S6-1), which may result in its non-phosphorylated degradation in the cytoplasm (Yin et al., 2002). PaLIC protein lacked a serine-rich domain (Supplementary Figure S6-4), which could affect its phosphorylation status (Zhang et al., 2012). PaSMOS1-2 protein exhibited a shortened WTNF domain, which is a transcriptional activation domain (Supplementary Figure S6-6), which may reduce its transactivation activity (Hirano et al., 2017). In terms of secondary structures, most of these proteins were similar, except for PmBZR3, RcDLT, and RcRAVL1-1_RcRAV6-1 proteins (Supplementary Figure S7 and Supplementary Table S7), indicating that these three proteins may share different protein spatial configuration. PcBZR3 and PaLIC proteins were different from their homologous proteins in terms of length and subcellular localization (Supplementary Table S4), which may be indicative of their special characters. The parallel biological functions of these homologous genes can be predicted from their similarities in evolutionary relationships, synteny, conserved domains, gene/protein structures, and chemical characteristics. However, several special genes should be studied in detail in the future.

### Potential Functions of Apple BR Downstream Genes Based on Their Expression Profiles

Phytohormones (e.g., IAA, CK, GA, and BR) play important functions at low concentrations in plants. BR downstream genes (except for *OSH1* and *SPY* genes) are not only regulated by BR but also affected by IAA, GA, and CK in several plants (Hu et al., 2004; Fan et al., 2018). However, little information about BR downstream genes is available in apple. Here, we investigated their expression patterns after BR and BRZ treatment (Figures 4, 5 and Supplementary Figures S10, S11). We found that most genes were either induced or repressed by BR within 2 h (Supplementary Figure S10). *MdILI1-2* and *MdSMOS1-2* were generally influenced by BR but not affected by BRZ (Supplementary Figure S11). These results indicate that BR downstream genes may show different roles in BR- and BRZ-mediated apple tree growth. Stem growth of apple trees is promoted by exogenous BR, IAA, and GA (Zheng et al., 2018). To reveal the functions of apple BR downstream genes, their expression patterns were investigated on days 0, 14, 28, 42, and 56 after BR treatment (Figures 4, 5). Results showed that *MdBZR1-3* and *MdBZR6* were up-regulated, *MdBZR4-5* and *MdBZR8* were down-regulated (Figure 4), and *MdBZR7* did not respond to BR treatment (Supplementary Figure S12). These findings suggest that like *Arabidopsis* and rice *BZR1/2* (Yin et al., 2002; Bai et al., 2007), some *MdBZR* genes may have positive roles in BR-mediated plant growth, but several may exhibit opposite functions. The expression levels of *MdDLT*, *MdRAVL1-1/MdRAV6-1*, *MdRAVL1-2/MdRAV6-2*, *MdSMOS1s*, and *MdGSR1* were repressed in BR-treated trees, while that of *MdLIC*, *MdILI1-1*, *MdILI1-2*, *MdCSA*, and *MdSPY2* was induced (Figure 5). Furthermore, *OsDLT*, *OsLIC*, *OsILI1*, and *OsSMOS1* are reported to be involved in controlling rice architecture (Wang et al., 2008; Qiao et al., 2017). Thus, *MdDLT*, *MdLIC*, *MdILI1s*, and *MdSMOS1s* may share similar functions to their rice homologous genes, and *MdRAVL1/MdRAV6*, *MdCSA*, *MdSPY*, and *MdGSR1* genes may participate in stem elongation. Our results also showed that *MdBZR1*, *MdBZR7-8*, *MdILI1-1*, *MdSMOS1-1*, and *MdSMOS1-3* were induced in the IAA-treated samples (Figures 4, 5). We identified an IAA-related *cis*-element (TGA-element) in the *MdBZR1*, *MdBZR7-8*, *MdILI1-1*, *MdSMOS1-1*, and *MdSMOS1-3* genes (Supplementary Figure S8). IAA is known to positively regulate *OsBZR1-2*, *OsILI1*, *OsSMOS1*, and *OsSMOS3* (Zhang et al., 2009; Qiao et al., 2017). In GA-treated STs, the transcription levels of *MdBZR1-4*, *MdBZR7-8*, *MdILI1-1*, *MdILI1-2*, and *MdGSR1* were obviously increased. Furthermore, GA-responsive elements (GARE-motif, P-box, or TATC-box) were identified in the *MdBZR1-4* genes (Supplementary Figure S8). These results suggest that GA and IAA likely influence these genes by binding to these *cis*-elements. CK is reported to increase primary shoot diameter in apple (Zheng et al., 2018). Here, during this process, *MdOSH1-1* and *MdOSH1-2* showed high expression in the CK-treated trees (Figure 5). In rice, *OsOSH1* is positively regulated by CK and is essential for plant size (Sentoku et al., 1999). These findings suggest that *MdOSH1-1* and *MdOSH1-2* may function in stem thickening. All above speculations should be verified by experiment.

Considering the pivotal roles of *BZRs*, *DLT*, *LIC*, *ILI1s*, and *SMOS1s* in stem elongation (Zhang et al., 2009; Qiao et al., 2017), we analyzed their expression patterns in YF-CF and CF/M9-CF/CF (Figure 6). With short nodes and fewer internodes, YF is a spur type of CF (Song et al., 2017). Dwarf rootstock (M9) has been widely used to inhibit apple tree elongation (Zheng et al., 2017). Here, the expression levels of *MdBZR1-2*, *MdBZR4*, *MdBZR6*, *MdDLT*, *MdILI1-1*, and *MdSMOS1s* were higher in CF than in YF at *p* < 0.05, whereas *MdLIC* was strongly expressed in YF (Figure 6). *MdBZR1* and *MdBZR4* were down-regulated but *MdBZR5-8*, *MdDLT*, *MdILI1s*, *MdSMOS1-1*, and *MdSMOS1-3* were up-regulated in CF/CF. These results imply that these genes may have various effects on apple shoot growth, which should be confirmed in the future.

Previous studies have reported that *BZR1-2* plays a role in stress resistance (Yang et al., 2016; Xie et al., 2019). Thus, we investigated the expression patterns of *MdBZR* genes under various stress treatments (Figure 7). ABA, MeJA, and SA are known for their roles in stress responses. We found that ABA induced *MdBZRs* (Figure 7), and ABA-responsive elements (ABREs) were recognized in all *MdBZR* genes (Supplementary Figure S8). AtBZR1 binds to the promoter of *ABI5* and AtBZR2 down-regulates *AtABI3* expression to repress ABA responses (Lopez-Molina et al., 2002; Ryu et al., 2014). In turn, ABA induces *AtBZR1/2* and phosphorylates AtBZR2 protein (Zhang et al., 2009). In maize, *ZmBZRs* are in response to exogenous ABA, functionally (Yu et al., 2018). Furthermore, *BvBZR* genes respond to ABA-mediated processes (Wang et al., 2009). Here, in MeJA-treated leaves, *MdBZR1-3*, *MdBZR5-6*, and *MdBZR8* were strongly expressed, while the expression levels of *MdBZR4* and *MdBZR7* were low. In addition, we identified at least one MeJA-responsive element in *MdBZR1-8* (Supplementary Figure S8), which may explain the possible effects of MeJA on *MdBZR1-8* expression. In sugar beet, certain *BvBZR* genes are up- and down-regulated by MeJA (Wang et al., 2009). In the current study, the expression levels of all *MdBZR* genes, except *MdBZR4*, were low in the SA-treated samples (Figure 7). In the *MdBZR2*, *MdBZR4-5*, and *MdBZR7-8* genes, several SA-related elements (TCA-element and SARE) were identified (Supplementary Figure S8). In *Eucalyptus grandis*, SA down-regulates *EgBZR* genes (Fan et al., 2018). Our results suggest that all *MdBZRs*, like *AtBZR1/2*, may potentially participate in ABA responses, several may positively or negatively regulate MeJA signals, and most may repress SA-induced defense reactions. We also found that drought stress repressed *MdBZR1* and *MdBZR4* expression levels, but up-regulated *MdBZR2-3* and *MdBZR6-7* (Figure 7). Salt treatment generally decreased the transcription levels of *MdBZR1-8* (Figure 7). In *Arabidopsis*, rapeseed, and eucalyptus, *BZR1/*2 genes are induced or inhibited by drought or salt (Fan et al., 2018). Legume *BZR* genes show different expression patterns in response to drought treatment (Li et al., 2018). After salt treatment, expression levels of legume *BZR* and *EgBZR* genes are down-regulated (Li et al., 2018). In our study, several defense and stress elements, including drought-induced motifs, were detected in the *MdBZR* genes (Supplementary Figure S8). These results indicate that *MdBZR* genes play roles in drought and salt stress, like other *BZR* genes. The information on *MdBZRs* involving in stress adaption should be discussed in transgenic plants.

### Diverse Roles of Apple BR Signaling Downstream Genes

**FIGURE 9:**
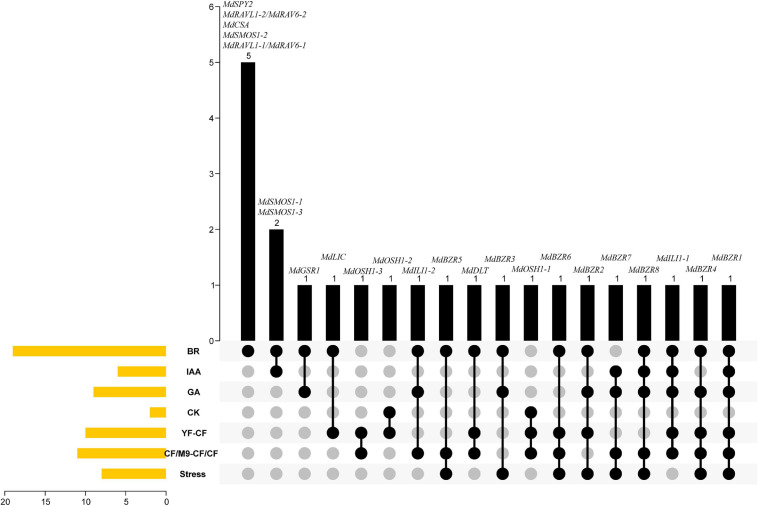
Venn diagram indicating various functions of apple BR downstream genes in growth and stress responses. The left side of figure shows the number of genes responding to BR, IAA, GA, CK, as well as stress, and differently expressed in YF-CF and CF/M9-CF/CF. Black columns display the details of genes that are in the above 1–7 classes.

Based on the above results, a Venn diagram was constructed to investigate the transcriptional responses of apple BR signaling downstream genes to BR, IAA, GA, CK, YF-CF, CF/M9-CF/CF, and stress (Figure 9). *MdSPY2*, *MdRAVL1-1/MdRAV6-1*, *MdRAVL1-2/MdRAV6-2*, *MdSMOS1-2*, and *MdCSA* were specifically controlled by BR. Six apple BR signaling downstream genes (*MdSMOS1-1*, *MdSMOS1-3, MdGSR1*, *MdLIC*, *MdOSH1-2*, and *MdOSH1-3*) were regulated by two factors. For example, *MdSMOS1-1* and *MdSMOS1-3* were regulated by BR and IAA; *MdGSR1* was influenced by BR and GA; *MdLIC* was altered by BR and showed differential expression between YF and CF. *MdILI1-2*, *MdBZR5*, *MdDLT*, *MdBZR3*, and *MdOSH1-1* were altered in response to three stimulations at the same time. *MdBZR2*, *MdBZR6*, and *MdBZR7* were differentially expressed in four groups. *MdBZR4*, *MdBZR8*, and *MdILI1-1* were up- or down-regulated by five treatments, simultaneously. *MdBZR1* was consistently regulated by BR, IAA, GA, and stress treatments, and was also differentially expressed in YF-CF and CF/M9-CF/CF. The responses of apple downstream genes to different clues suggest their various functions, but their functional information should be obtained in a detailed study.

## Conclusion

In this study, a total of 196 BR downstream genes were systematically identified from *Arabidopsis* and nine Rosaceae species. Their chromosomal locations, transmembrane helices, conserved domains, chemical characterizations, phylogenetic tree, gene/protein structures, synteny relationships, Ka/Ks ratios, promoter sequences, protein–protein interactions, and potential functions were analyzed. We found that gene duplication events (mainly segmental duplication and WGD) resulted in BR downstream gene expansion through purifying selection and diverse evolutionary patterns. Their functions were similar to their rice homologous genes. However, several genes (*PaBZR1*, *PcBZR1-3*, *PmBZR3*, *RcBZR4*, *PaLIC*, *PaSMOS1-2*, and all *DLTs* and *RAVL1*/*RAV6s* except for *MdRAVL1*/*MdRAV6*) may be species-specific, as predicted from the instability, transmembrane structure, subcellular localization, gene structure, conserved domain analyses, and responses to exogenous factors. Moreover, many apple BR downstream genes were predicted to control apple tree architecture via various hormones (BR, IAA, GA, and CK) and may play vital functions in shoot elongation of different varieties and grafting combinations. Furthermore, several *MdBZR* genes appeared to respond to stress-related signals. *MdBZR1* was identified as a core gene due to its various roles, and *MdBZR2-8*, *MdILI1-1*, *MdILI1-2*, *MdDLT*, and *MdOSH1-1* should also be noted.

## Data Availability Statement

The original contributions presented in the study are included in the article/Supplementary Material, further inquiries can be directed to the corresponding author/s.

## Author Contributions

JH designed the experiment. LZ, YY, SM, JC, and CY performed the experiments. LZ and YW analyzed the data. LZ drafted the manuscript. JH revised the final version of the manuscript. WW, MS, and XH assisted with revisions to the manuscript. All authors contributed to the article and approved the submitted version.

## Conflict of Interest

The authors declare that the research was conducted in the absence of any commercial or financial relationships that could be construed as a potential conflict of interest.

## Footnotes

1 https://www.arabidopsis.org/

2 http://pfam.xfam.org/

3 http://smart.embl-heidelberg.de/

4 http://string-db.org/

5 http://www.sbg.bio.ic.ac.uk/phyre2/html/page.cgi?id=index

6 https://npsa-prabi.ibcp.fr/cgi-bin/npsa_automat.pl?page=npsa_sopma.html

7 http://www.cbs.dtu.dk/services/TMHMM/

8 http://web.expasy.org/protparam/

9 http://www.cambia.org

## Supplementary Material

Supplementary Figure 1 Chromosomal location of BR downstream genes in nine Rosaceae species.

Supplementary Figure 2 Transmembrane topology analysis of BR downstream proteins. Red peaks indicate predicted transmembrane helices.

Supplementary Figure 3 Synteny analysis of BR downstream genes in each Rosaceae species.

Supplementary Figure 4 Synteny analysis among BR downstream genes in *Oryza sativa* and each Rosaceae species.

Supplementary Figure 5 Structure of BR downstream genes in nine Rosaceae species.

Supplementary Figure 6 Alignment of multiple BR downstream proteins in *Arabidopsis*, rice, and nine Rosaceae species.

Supplementary Figure 7 Predicted secondary structures of BR downstream proteins.

Supplementary Figure 8 Promoter analysis of apple BR downstream genes.

Supplementary Figure 9 Protein-protein interaction analysis of apple BR downstream proteins.

Supplementary Figure 10 Response of BR downstream genes to exogenous BR treatment over 2 h. Each value represents mean ± standard error of three biological replicates. Means followed by small letters are significantly different at 0.05 level (^∗^).

Supplementary Figure 11 Response of BR downstream genes to exogenous BRZ treatment over 2 h. Each value represents mean ± standard error of three biological replicates. Means followed by small letters are significantly different at 0.05 level (^∗^).

Supplementary Figure 12 Irregular expression patterns of BR downstream genes in response to different treatments. Each value represents mean ± standard error of three biological replicates. ^∗^ indicates significant differences at 0.05.

Supplementary Figure 13 Nucleotide sequence alignment between cloned and reference *MdBZR* genes. **(A)** *MdBZR1* gene; **(B)** *MdBZR4* gene; **(C)** *MdBZR6* gene; **(D)** *MdBZR7* gene.

Supplementary Figure 14 Protein sequence alignment between cloned and reference *MdBZR* genes. **(A)** MdBZR1 protein; **(B)** MdBZR4 protein; **(C)** MdBZR6 protein; **(D)** MdBZR7 protein.

Supplementary Table 1 The roles of BR signaling downstream genes in rice.

Supplementary Table 2 Summary of plant materials and treatments.

Supplementary Table 3 Primer sequences for qRT-PCR, gene cloning, and subcellular localization analyses.

Supplementary Table 4 Details regarding BR downstream genes in *Arabidopsis*, rice, and nine Rosaceae species.

Supplementary Table 5 Ka, Ks, and Ka/Ks ratios among corresponding orthologous BR downstream genes in each Rosaceae species.

Supplementary Table 6 Ka, Ks, and Ka/Ks ratios among corresponding orthologous BR downstream genes between *Oryza sativa* and each Rosaceae species.

Supplementary Table 7 Secondary structure analysis of BR downstream proteins.

Supplementary Table 8 GO and KEGG analyses of interactive proteins identified in Supplementary Figure 9.

## References

[B1] Bai M. Y. Zhang L. Y. Gampala S. S. Zhu S. W. Song W. Y. Chong K. (2007). Functions of OsBZR1 and 14-3-3 proteins in brassinosteroid signaling in rice. *Proc. Natl. Acad. Sci. U. S. A.* 104 13839–13844. 10.1073/pnas.0706386104 17699623 PMC1959469

[B2] Chen C. Chen H. Zhang Y. Thomas H. R. Frank M. H. He Y. (2020). TBtools: an integrative toolkit developed for interactive analyses of big biological data. *Mol. Plant* 13 1194–1202. 10.1016/j.molp.2020.06.009 32585190

[B3] Chen H. Zuo X. Shao H. Fan S. Ma J. Zhang D. (2018). Genome-wide analysis of carotenoid cleavage oxygenase genes and their responses to various phytohormones and abiotic stresses in apple (*Malus domestica*). *Plant Physiol. Biochem.* 123 81–93. 10.1016/j.plaphy.2017.12.001 29223850

[B4] Fan C. Guo G. Yan H. Qiu Z. Liu Q. Zeng B. (2018). Characterization of Brassinazole resistant (BZR) gene family and stress induced expression in *Eucalyptus grandis*. *Physiol. Mol. Biol. Plants* 24 821–831. 10.1007/s12298-018-0543-2 30150857 PMC6103948

[B5] Feng C. Wang J. Harris A. J. Folta K. M. Zhao M. Kang M. (2020). Tracing the diploid ancestry of the cultivated octoploid strawberry. *Mol. Biol. Evol.* 38 478–485. 10.1093/molbev/msaa238 32941604 PMC7826170

[B6] Feng Y. Sun Q. Zhang G. Wu T. Zhang X. Xu X. (2019). Genome-wide identification and characterization of ABC transporters in nine rosaceae species identifying MdABCG28 as a possible cytokinin transporter linked to dwarfing. *Int. J. Mol. Sci.* 20:5783. 10.3390/ijms20225783 31744249 PMC6887749

[B7] Gambino G. Perrone I. Gribaudo I. (2008). A rapid and effective method for RNA extraction from different tissues of grapevine and other woody plants. *Phytochem. Anal.* 19 520–525. 10.1002/pca.1078 18618437

[B8] Hirano K. Yoshida H. Aya K. Kawamura M. Hayashi M. Hobo T. (2017). SMALL ORGAN SIZE 1 and SMALL ORGAN SIZE 2/DWARF and LOW-TILLERING form a complex to integrate auxin and brassinosteroid signaling in rice. *Mol. Plant* 10 590–604. 10.1016/j.molp.2016.12.013 28069545

[B9] Hu Y. X. Wang Y. X. Liu X. F. Li J. Y. (2004). *Arabidopsis* RAV1 is down-regulated by brassinosteroid and may act as a negative regulator during plant development. *Cell Res.* 14 8–15. 10.1038/sj.cr.7290197 15040885

[B10] Kang S. Yang F. Li L. Chen H. Chen S. Zhang J. (2015). The *Arabidopsis* transcription factor BRASSINOSTEROID INSENSITIVE1-ETHYL METHANESULFONATE-SUPPRESSOR1 is a direct substrate of MITOGEN-ACTIVATED PROTEIN KINASE6 and regulates immunity. *Plant Physiol.* 167 1076–1086. 10.1104/pp.114.250985 25609555 PMC4348755

[B11] Li C. Tan D. X. Liang D. Chang C. Jia D. Ma F. (2015). Melatonin mediates the regulation of ABA metabolism, free-radical scavenging, and stomatal behaviour in two Malus species under drought stress. *J. Exp. Bot.* 66 669–680. 10.1093/jxb/eru476 25481689

[B12] Li W. Wu J. Weng S. Zhang Y. Zhang D. Shi C. (2010). Identification and characterization of dwarf 62, a loss-of-function mutation in DLT/OsGRAS-32 affecting gibberellin metabolism in rice. *Planta* 232 1383–1396. 10.1007/s00425-010-1263-1 20830595

[B13] Li Y. He L. Li J. Chen J. Liu C. (2018). Genome-wide identification, characterization, and expression profiling of the legume BZR transcription factor gene family. *Front. Plant Sci.* 9:1332. 10.3389/fpls.2018.01332 30283468 PMC6156370

[B14] Livak K. J. Schmittgen T. D. (2001). Analysis of relative gene expression data using real-time quantitative PCR and the 2(-Delta Delta C(T)) method. *Methods* 25 402–408. 10.1006/meth.2001.1262 11846609

[B15] Lopez-Molina L. Mongrand S. McLachlin D. T. Chait B. T. Chua N. H. (2002). ABI5 acts downstream of ABI3 to execute an ABA-dependent growth arrest during germination. *Plant J.* 32 317–328. 12410810 10.1046/j.1365-313x.2002.01430.x

[B16] Ma B. Luo Y. Jia L. Qi X. Zeng Q. Xiang Z. (2014). Genome-wide identification and expression analyses of cytochrome P450 genes in mulberry (*Morus notabilis*). *J. Integr. Plant Biol.* 56 887–901. 10.1111/jipb.12141 24304637

[B17] Miyaji T. Yamagami A. Kume N. Sakuta M. Osada H. Asami T. (2014). Brassinosteroid-related transcription factor BIL1/BZR1 increases plant resistance to insect feeding. *Biosci. Biotechnol. Biochem.* 78 960–968. 10.1080/09168451.2014.910093 25036120

[B18] Ohnishi T. Szatmari A. M. Watanabe B. Fujita S. Bancos S. Koncz C. (2006). C-23 hydroxylation by *Arabidopsis* CYP90C1 and CYP90D1 reveals a novel shortcut in brassinosteroid biosynthesis. *Plant Cell.* 18 3275–3288. 10.1105/tpc.106.045443 17138693 PMC1693957

[B19] Ou C. Wang F. Wang J. Li S. Zhang Y. Fang M. (2019). A de novo genome assembly of the dwarfing pear rootstock Zhongai 1. *Sci. Data* 6:281. 10.1038/s41597-019-0291-3 31767847 PMC6877535

[B20] Poppenberger B. Berthiller F. Lucyshyn D. Sieberer T. Schuhmacher R. Krska R. (2003). Detoxification of the *Fusarium* mycotoxin deoxynivalenol by a UDP-glucosyltransferase from *Arabidopsis thaliana*. *J. Biol. Chem.* 278 47905–47914. 10.1074/jbc.M307552200 12970342

[B21] Qiao S. Sun S. Wang L. Wu Z. Li C. Li X. (2017). The RLA1/SMOS1 transcription factor functions with OsBZR1 to regulate brassinosteroid signaling and rice architecture. *Plant Cell* 29 292–309. 10.1105/tpc.16.00611 28100707 PMC5354187

[B22] Ryu H. Cho H. Bae W. Hwang I. (2014). Control of early seedling development by BES1/TPL/HDA19-mediated epigenetic regulation of ABI3. *Nat. Commun.* 5:4138. 10.1038/ncomms5138 24938150

[B23] Salazar-Henao J. E. Lehner R. Betegon-Putze I. Vilarrasa-Blasi J. Cano-Delgado A. I. (2016). BES1 regulates the localization of the brassinosteroid receptor BRL3 within the provascular tissue of the *Arabidopsis* primary root. *J. Exp. Bot.* 67 4951–4961. 10.1093/jxb/erw258 27511026 PMC5014150

[B24] Sentoku N. Sato Y. Kurata N. Ito Y. Kitano H. Matsuoka M. (1999). Regional expression of the rice KN1-type homeobox gene family during embryo, shoot, and flower development. *Plant Cell* 11 1651–1664. 10.1105/tpc.11.9.1651 10488233 PMC144314

[B25] Shimada A. Ueguchi-Tanaka M. Sakamoto T. Fujioka S. Takatsuto S. Yoshida S. (2006). The rice SPINDLY gene functions as a negative regulator of gibberellin signaling by controlling the suppressive function of the DELLA protein. SLR1, and modulating brassinosteroid synthesis. *Plant J.* 48 390–402. 10.1111/j.1365-313X.2006.02875.x 17052323

[B26] Singh A. P. Savaldi-Goldstein S. (2015). Growth control: brassinosteroid activity gets context. *J. Exp. Bot.* 66 1123–1132. 10.1093/jxb/erv026 25673814

[B27] Song C. Zhang D. Zheng L. Zhang J. Zhang B. Luo W. (2017). MiRNA and degradome sequencing reveal miRNA and their target genes that may mediate shoot growth in spur type mutant “Yanfu 6”. *Front. Plant Sci.* 8:441. 10.3389/fpls.2017.00441 28424721 PMC5371658

[B28] Tong H. Chu C. (2018). Functional specificities of brassinosteroid and potential utilization for crop improvement. *Trends Plant Sci.* 23 1016–1028. 10.1016/j.tplants.2018.08.007 30220494

[B29] Tong H. Jin Y. Liu W. Li F. Fang J. Yin Y. (2009). DWARF and LOW-TILLERING, a new member of the GRAS family, plays positive roles in brassinosteroid signaling in rice. *Plant J.* 58 803–816. 10.1111/j.1365-313X.2009.03825.x 19220793

[B30] Tong H. Xiao Y. Liu D. Gao S. Liu L. Yin Y. (2014). Brassinosteroid regulates cell elongation by modulating gibberellin metabolism in rice. *Plant Cell* 26 4376–4393. 10.1105/tpc.114.132092 25371548 PMC4277228

[B31] Tsuda K. Ito Y. Sato Y. Kurata N. (2011). Positive autoregulation of a KNOX gene is essential for shoot apical meristem maintenance in rice. *Plant Cell* 23 4368–4381. 10.1105/tpc.111.090050 22207572 PMC3269871

[B32] Tsuda K. Kurata N. Ohyanagi H. Hake S. (2014). Genome-wide study of KNOX regulatory network reveals brassinosteroid catabolic genes important for shoot meristem function in rice. *Plant Cell* 26 3488–3500. 10.1105/tpc.114.129122 25194027 PMC4213158

[B33] Vriet C. Russinova E. Reuzeau C. (2013). From squalene to brassinolide: the steroid metabolic and signaling pathways across the plant kingdom. *Mol. Plant* 6 1738–1757. 10.1093/mp/sst096 23761349

[B34] Wang L. Guo K. Li Y. Tu Y. Hu H. Wang B. (2010). Expression profiling and integrative analysis of the CESA/CSL superfamily in rice. *BMC Plant Biol.* 10:282. 10.1186/1471-2229-10-282 21167079 PMC3022907

[B35] Wang L. Wang Z. Xu Y. Joo S. H. Kim S. K. Xue Z. (2009). OsGSR1 is involved in crosstalk between gibberellins and brassinosteroids in rice. *Plant J.* 57 498–510. 10.1111/j.1365-313X.2008.03707.x 18980660

[B36] Wang L. Xu Y. Zhang C. Ma Q. Joo S. H. Kim S. K. (2008). OsLIC, a novel CCCH-Type zinc finger protein with transcription activation, mediates rice architecture via brassinosteroids signaling. *PLoS One* 3:e3521. 10.1371/journal.pone.0003521 18953406 PMC2567845

[B37] Wang W. Sun Y. Q. Li G. L. Zhang S. Y. (2019). Genome-wide identification, characterization, and expression patterns of the BZR transcription factor family in sugar beet (*Beta vulgaris* L.). *BMC Plant Biol.* 19:191. 10.1186/s12870-019-1783-1 31072335 PMC6506937

[B38] Wang X. Zhao X. Zhu J. Wu W. (2005). Genome-wide investigation of intron length polymorphisms and their potential as molecular markers in rice (Oryza sativa L.). *DNA Res.* 12 417–427. 10.1093/dnares/dsi019 16769698

[B39] Wang Z. Y. Bai M. Y. Oh E. Zhu J. Y. (2012). Brassinosteroid signaling network and regulation of photomorphogenesis. *Annu. Rev. Genet.* 46 701–724. 10.1146/annurev-genet-102209-163450 23020777

[B40] Wei F. Coe E. Nelson W. Bharti A. K. Engler F. Butler E. (2007). Physical and genetic structure of the maize genome reflects its complex evolutionary history. *PLoS Genet.* 3:e123. 10.1371/journal.pgen.0030123 17658954 PMC1934398

[B41] Xie L. Yang C. Wang X. (2011). Brassinosteroids can regulate cellulose biosynthesis by controlling the expression of CESA genes in *Arabidopsis*. *J. Exp. Bot.* 62 4495–4506. 10.1093/jxb/err164 21617247 PMC3170551

[B42] Xie Z. Nolan T. Jiang H. Tang B. Zhang M. Li Z. (2019). The AP2/ERF transcription factor TINY modulates brassinosteroid-regulated plant growth and drought responses in arabidopsis. *Plant Cell* 31 1788–1806. 10.1105/tpc.18.00918 31126980 PMC6713308

[B43] Xu G. Guo C. Shan H. Kong H. (2012). Divergence of duplicate genes in exon-intron structure. *Proc. Natl. Acad. Sci. U. S. A.* 109 1187–1192. 10.1073/pnas.1109047109 22232673 PMC3268293

[B44] Yang S. Zhang X. Yue J. X. Tian D. Chen J. Q. (2008). Recent duplications dominate NBS-encoding gene expansion in two woody species. *Mol. Genet. Genomics* 280 187–198. 10.1007/s00438-008-0355-0 18563445

[B45] Yang X. Bai Y. Shang J. Xin R. Tang W. (2016). The antagonistic regulation of abscisic acid-inhibited root growth by brassinosteroids is partially mediated via direct suppression of ABSCISIC ACID INSENSITIVE 5 expression by BRASSINAZOLE RESISTANT 1. *Plant Cell Environ.* 39 1994–2003. 10.1111/pce.12763 27149247

[B46] Yin Y. Vafeados D. Tao Y. Yoshida S. Asami T. Chory J. (2005). A new class of transcription factors mediates brassinosteroid-regulated gene expression in *Arabidopsis*. *Cell* 120 249–259. 10.1016/j.cell.2004.11.044 15680330

[B47] Yin Y. Wang Z. Y. Mora-Garcia S. Li J. Yoshida S. Asami T. (2002). BES1 accumulates in the nucleus in response to brassinosteroids to regulate gene expression and promote stem elongation. *Cell* 109 181–191. 10.1016/s0092-8674(02)00721-3 12007405

[B48] Yoo S. D. Cho Y. H. Sheen J. (2007). *Arabidopsis* mesophyll protoplasts: a versatile cell system for transient gene expression analysis. *Nat. Protoc.* 2 1565–1572. 10.1038/nprot.2007.199 17585298

[B49] Yu H. Feng W. Sun F. Zhang Y. Y. Qu J. T. Liu B. (2018). Cloning and characterization of BES1/BZR1 transcription factor genes in maize. *Plant Growth Regul.* 86 235–249. 10.1007/s10725-018-0424-2

[B50] Zhang C. Xu Y. Guo S. Zhu J. Huan Q. Liu H. (2012). Dynamics of brassinosteroid response modulated by negative regulator LIC in rice. *PLoS Genet.* 8:e1002686. 10.1371/journal.pgen.1002686 22570626 PMC3343102

[B51] Zhang L. Y. Bai M. Y. Wu J. Zhu J. Y. Wang H. Zhang Z. (2009). Antagonistic HLH/bHLH transcription factors mediate brassinosteroid regulation of cell elongation and plant development in rice and *Arabidopsis*. *Plant Cell* 21 3767–3780. 10.1105/tpc.109.070441 20009022 PMC2814508

[B52] Zhang X. Sun J. Cao X. Song X. (2015). Epigenetic mutation of RAV6 affects leaf angle and seed size in rice. *Plant Physiol.* 169 2118–2128. 10.1104/pp.15.00836 26351308 PMC4634063

[B53] Zheng L. Ma J. Song C. An N. Zhang D. Zhao C. (2017). Genome-wide identification and expression profiling analysis of brassinolide signal transduction genes regulating apple tree architecture. *Acta Physiol. Plant.* 39:177. 10.1007/s11738-017-2479-5

[B54] Zheng L. Yang Y. Gao C. Ma J. Shah K. Zhang D. (2019). Transcriptome analysis reveals new insights into MdBAK1-mediated plant growth in *Malus domestica*. *J. Agric. Food Chem.* 10.1021/acs.jafc.9b02467 31373492

[B55] Zheng L. Zhao C. Mao J. Song C. Ma J. Zhang D. (2018). Genome-wide identification and expression analysis of brassinosteroid biosynthesis and metabolism genes regulating apple tree shoot and lateral root growth. *J. Plant Physiol.* 231 68–85. 10.1016/j.jplph.2018.09.002 30223145

[B56] Zhu X. Liang W. Cui X. Chen M. Yin C. Luo Z. (2015). Brassinosteroids promote development of rice pollen grains and seeds by triggering expression of carbon starved anther, a MYB domain protein. *Plant J.* 82 570–581. 10.1111/tpj.12820 25754973

