# Supplementary material for: Genome-Wide Identification of Brassinosteroid Signaling Downstream Genes in Nine Rosaceae Species and Analyses of Their Roles in Stem Growth and Stress Response in Apple
Source: Front Genet. 2021 Mar 18;12:640271. doi: 10.3389/fgene.2021.640271 (PMC8012692; doi:10.3389/fgene.2021.640271)

**Supplemental Figure 1. Chromosomal location of BR downstream genes in nine rosaceae species**

**Supplemental Figure 1-1 Chromosomal location of BR downstream genes in *Malus domestica***


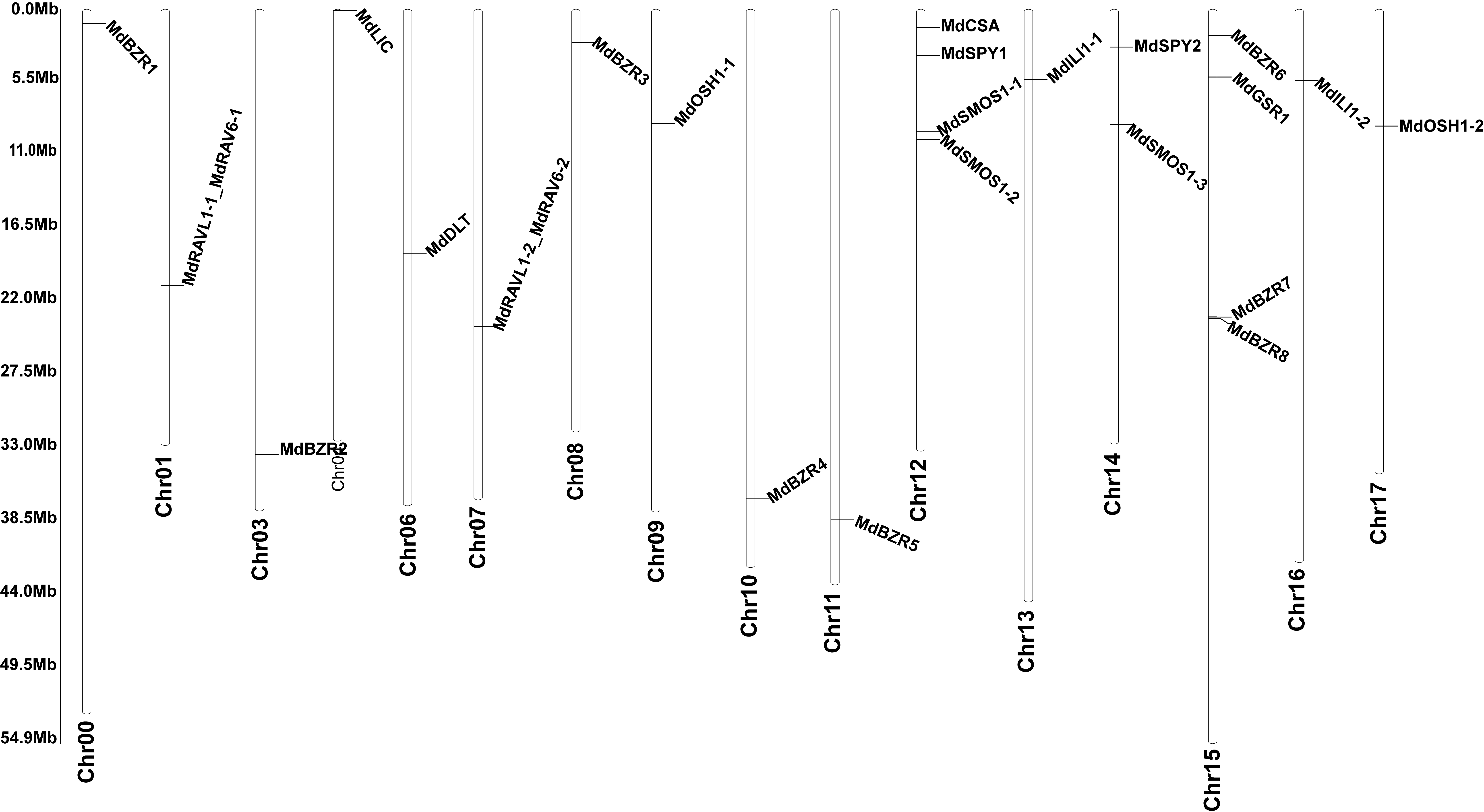


**Supplemental Figure 1-2 Chromosomal location of BR downstream genes in *Fragaria vesca***


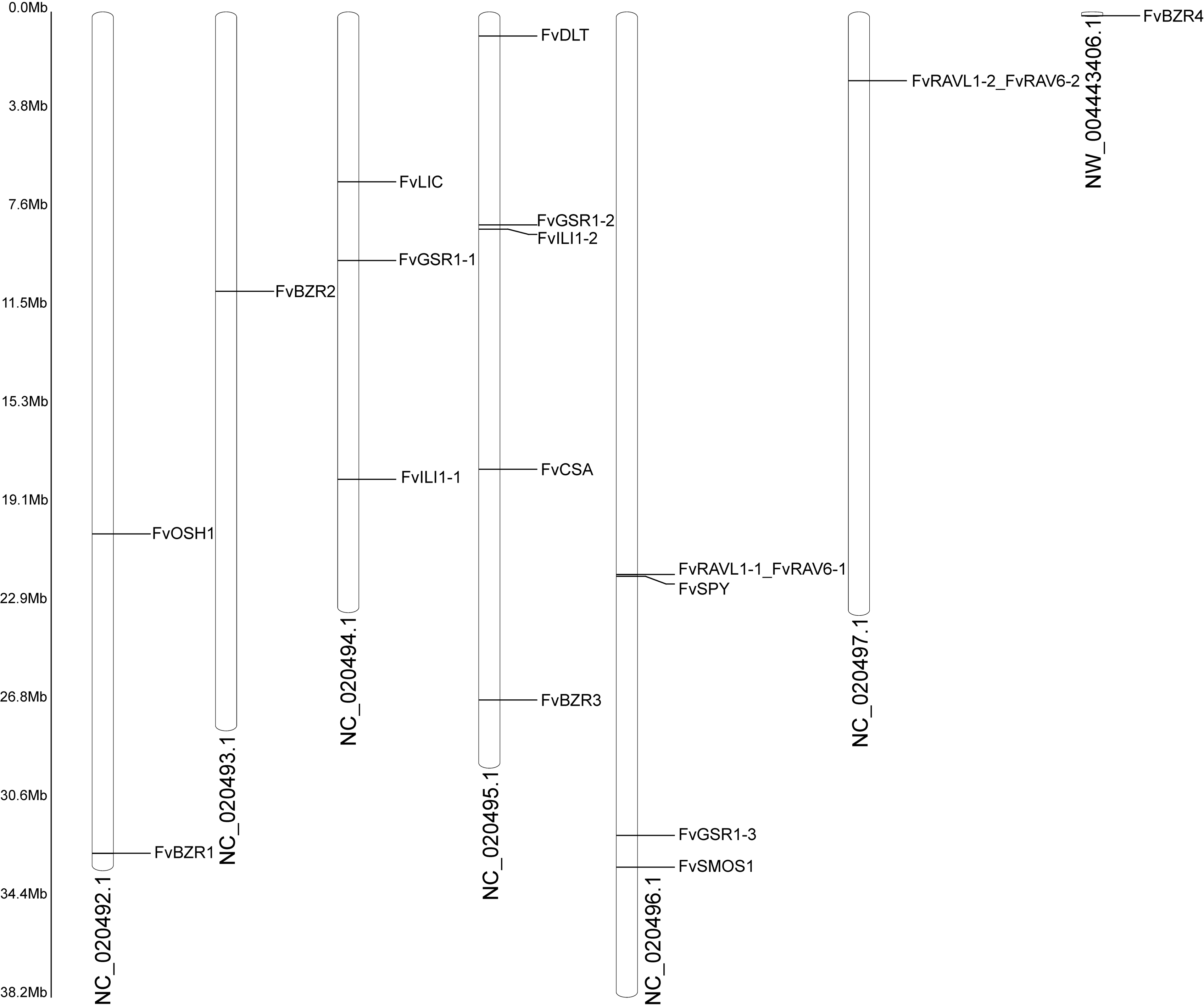


**Supplemental Figure 1-3 Chromosomal location of BR downstream genes in *Rubus occidentalis***


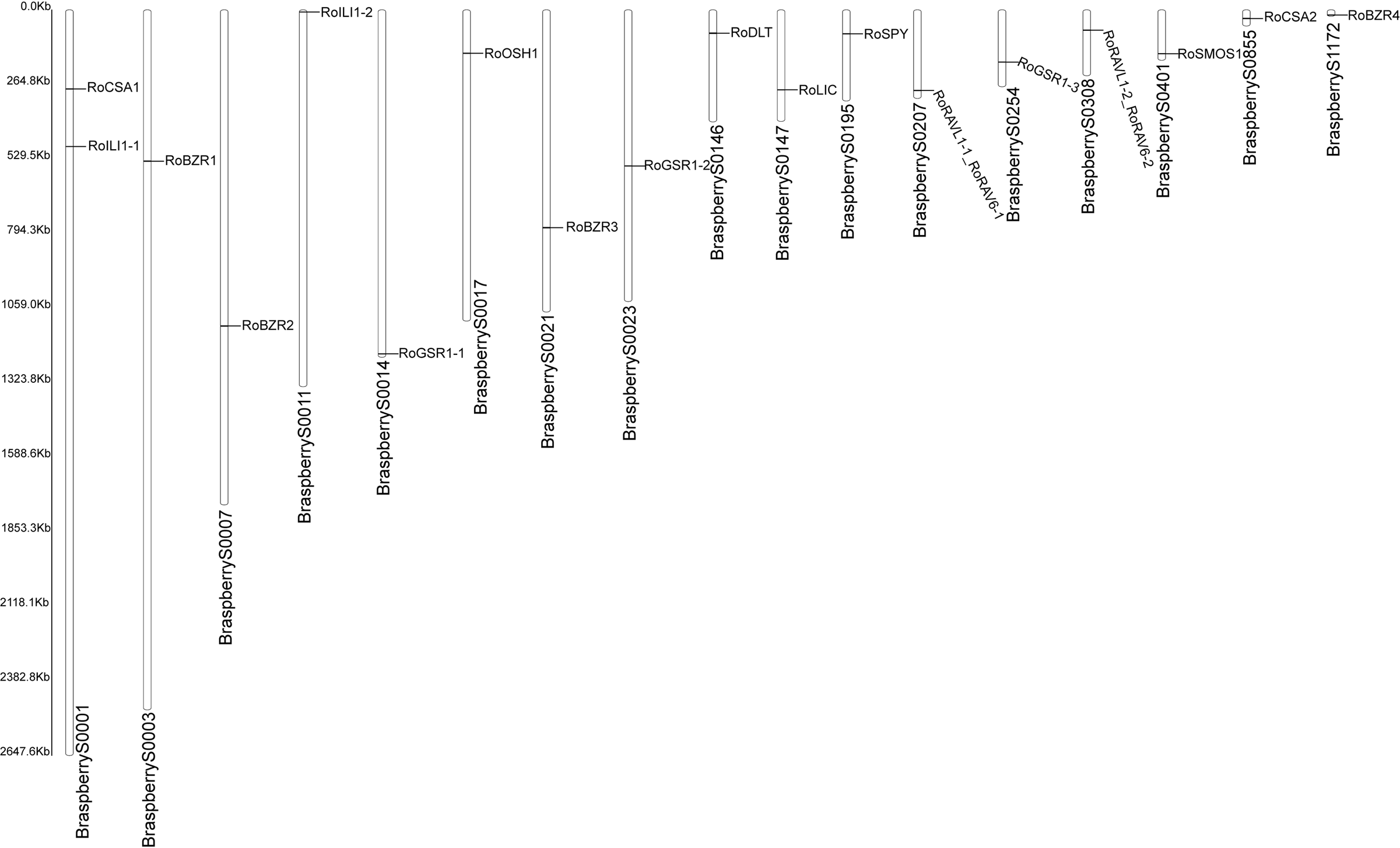


**Supplemental Figure 1-4 Chromosomal location of BR downstream genes in *Pyrus communis***


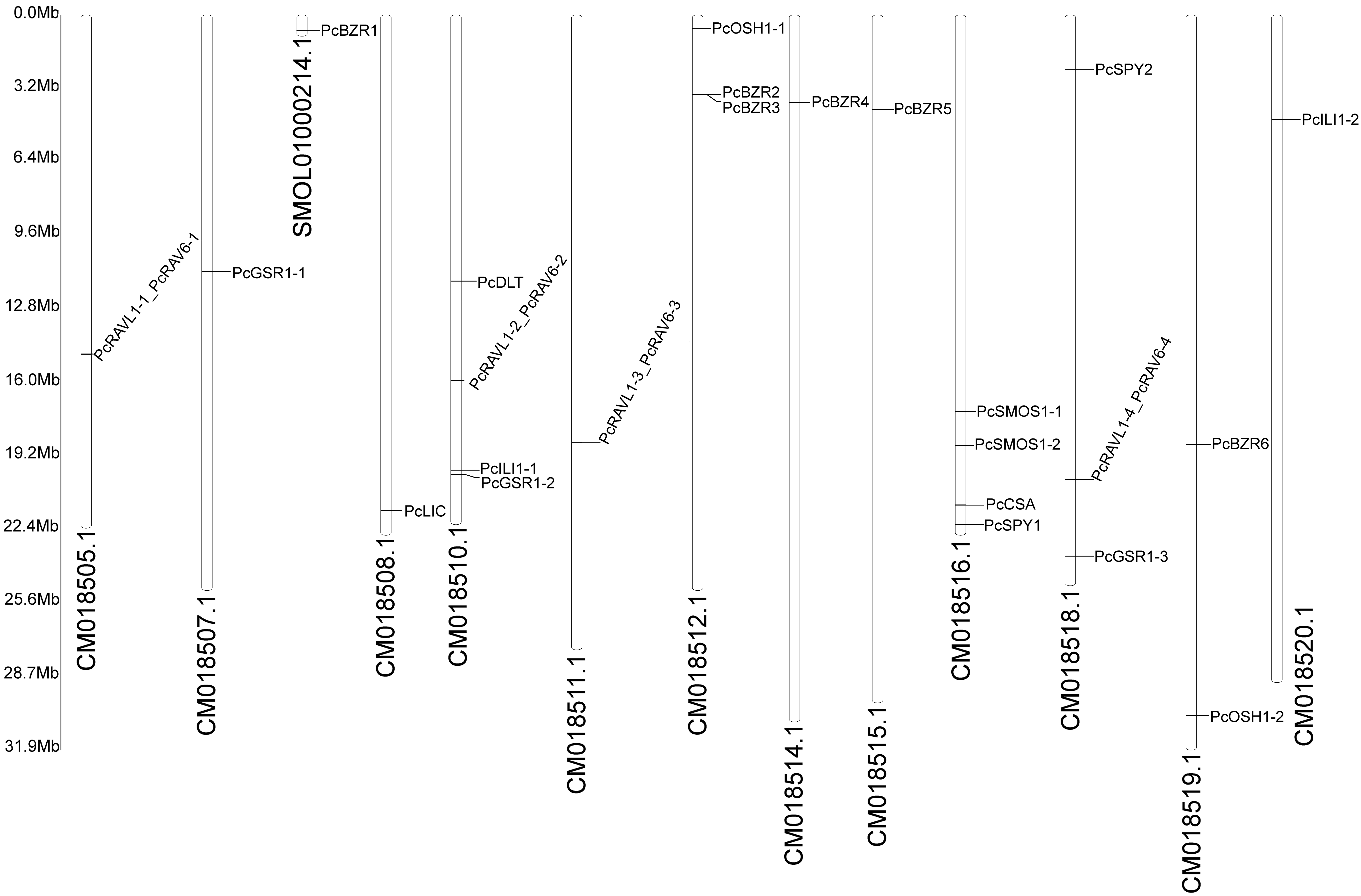


**Supplemental Figure 1-5 Chromosomal location of BR downstream genes in *Prunus persica***


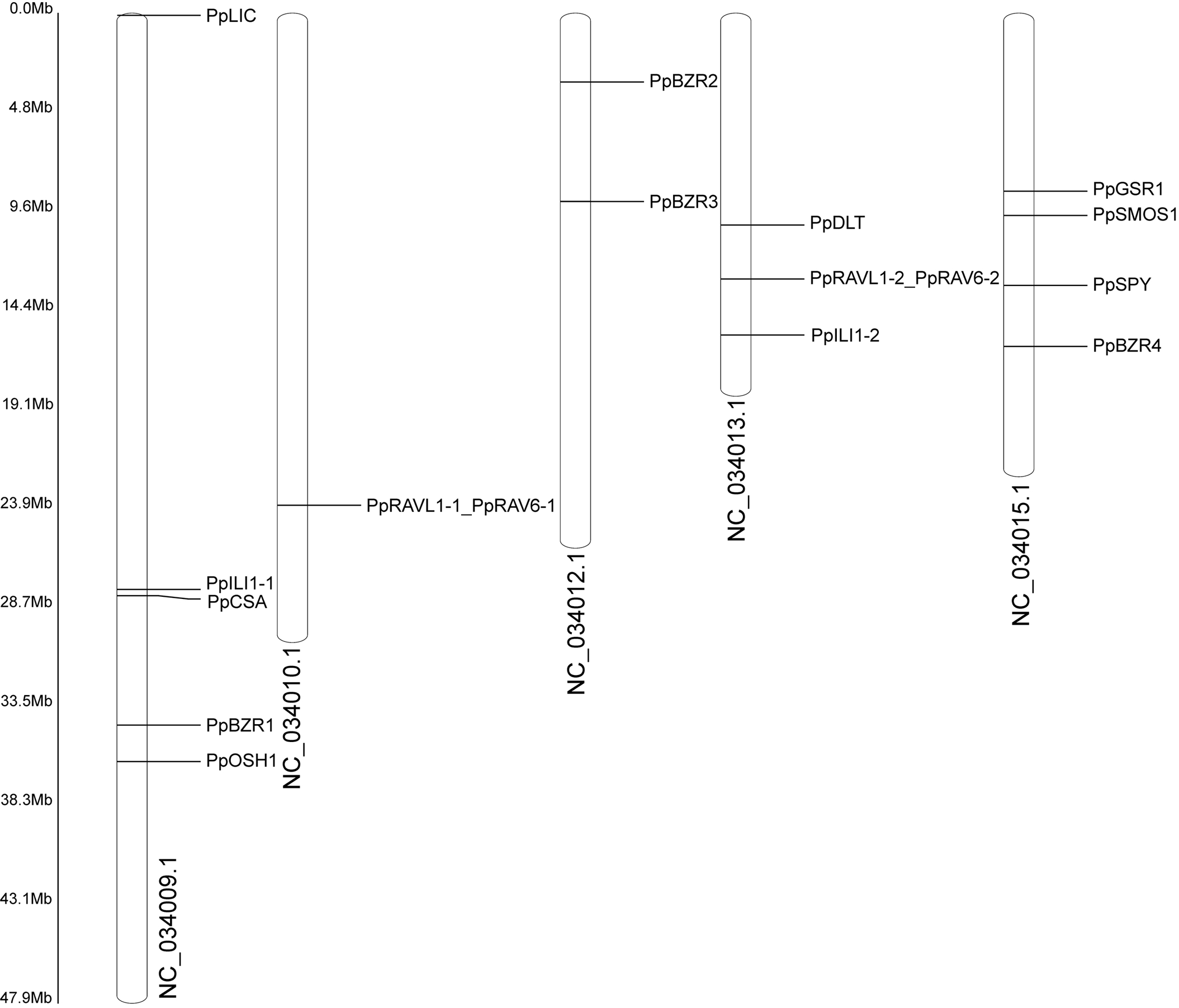


**Supplemental Figure 1-6 Chromosomal location of BR downstream genes in *Prunus avium***


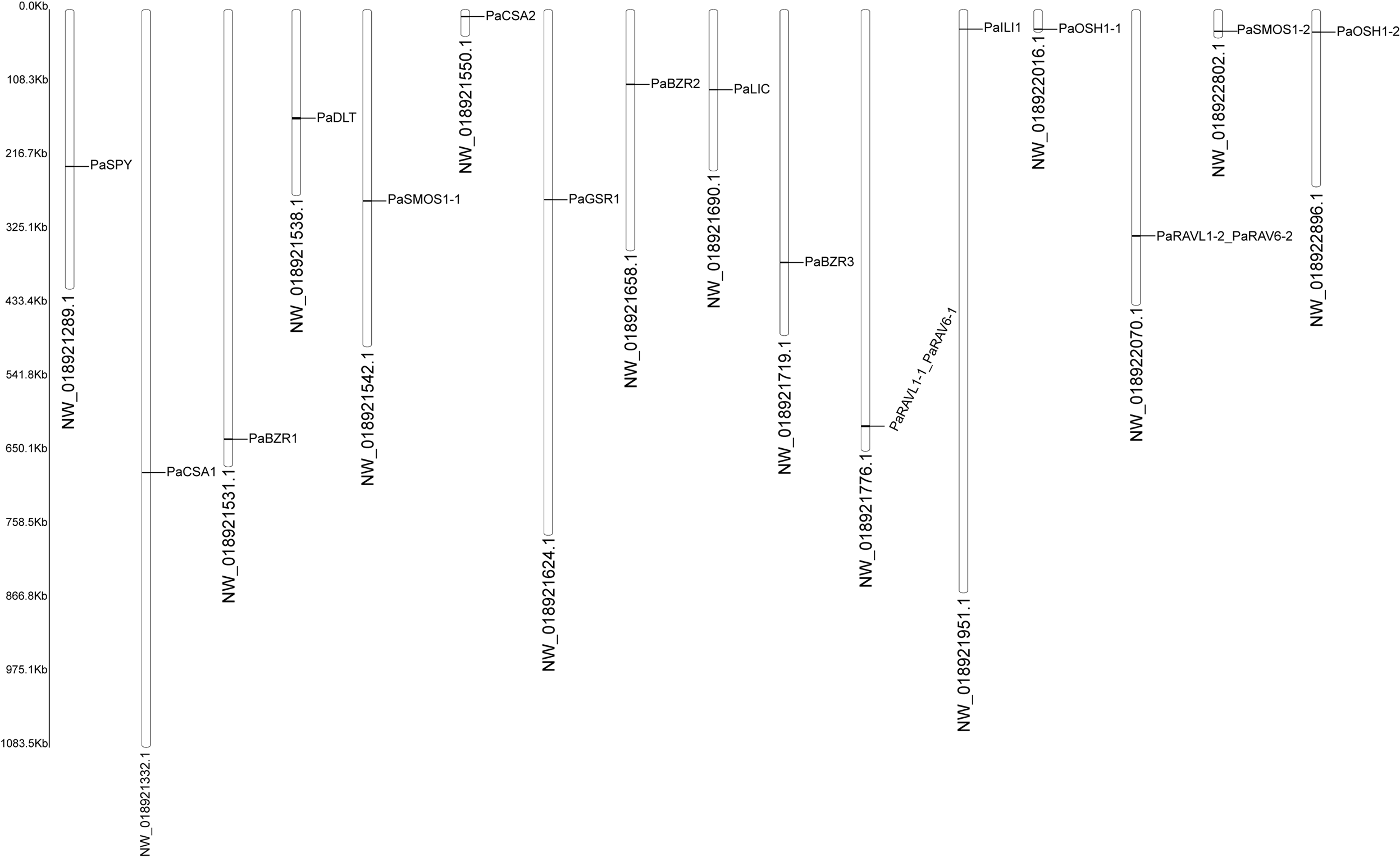


**Supplemental Figure 1-7 Chromosomal location of BR downstream genes in *Prunus dulcis***


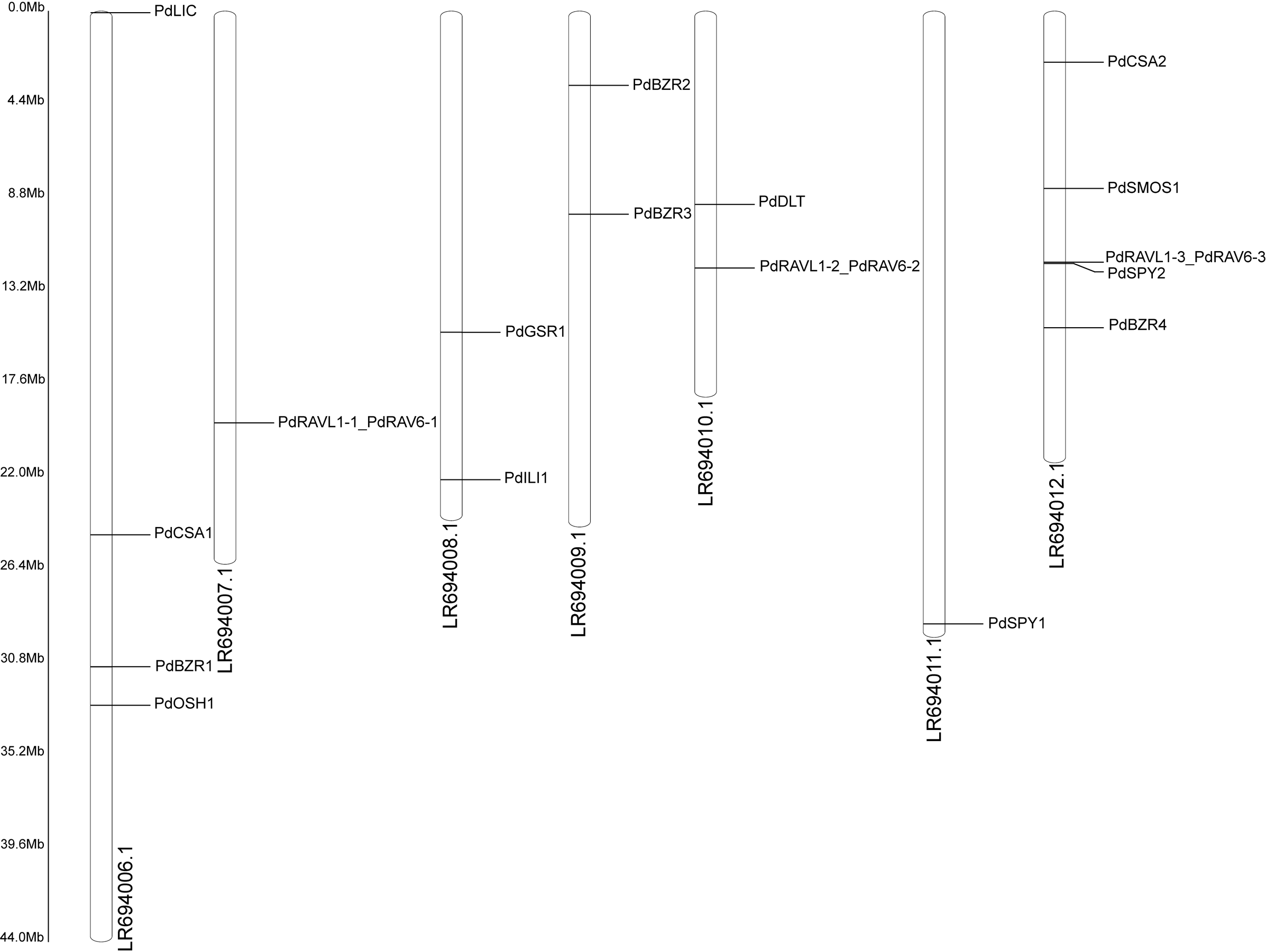


**Supplemental Figure 1-8 Chromosomal location of BR downstream genes in *Rosa chinensis***


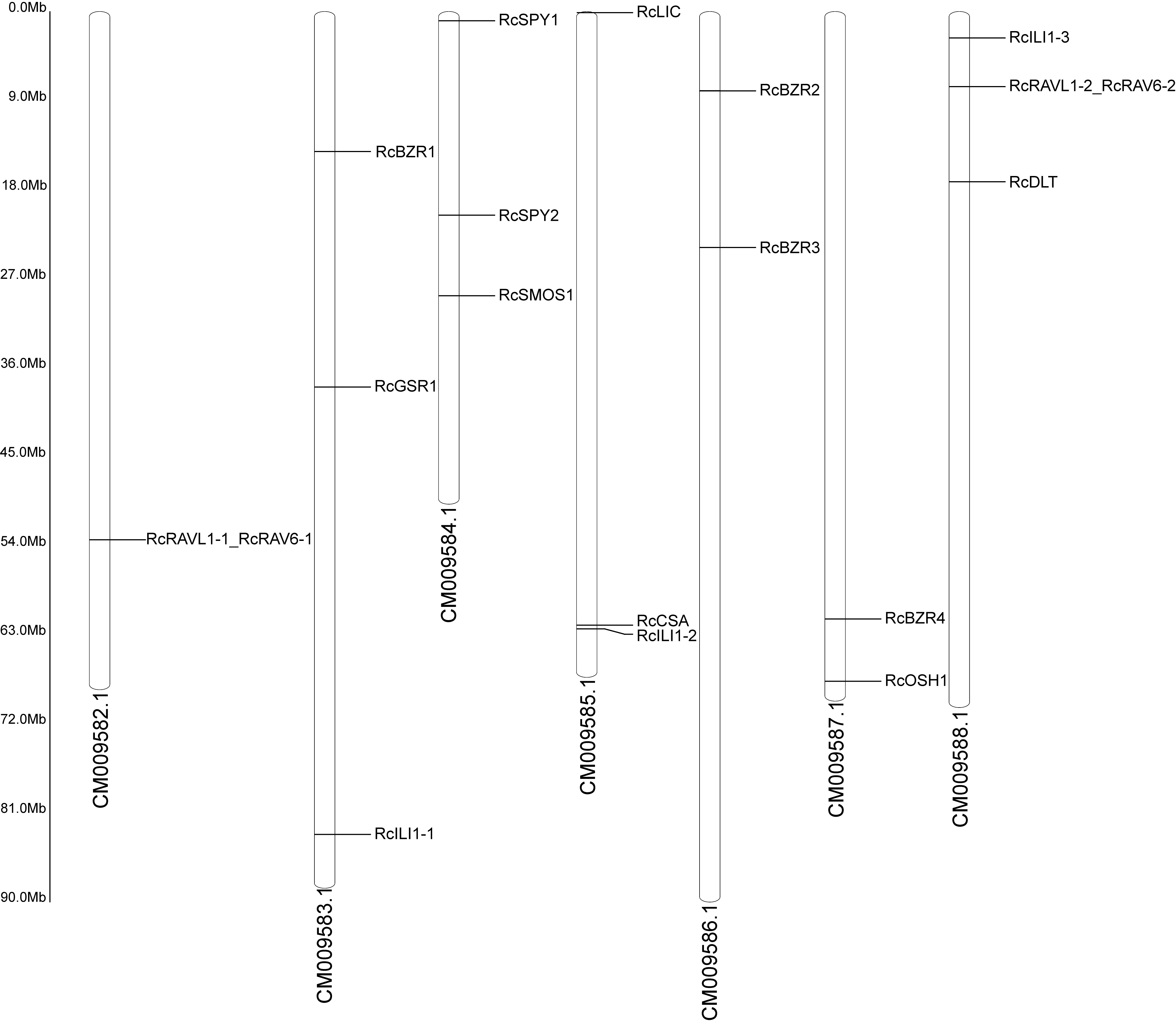


**Supplemental Figure 1-9 Chromosomal location of BR downstream genes in *Prunus mume***


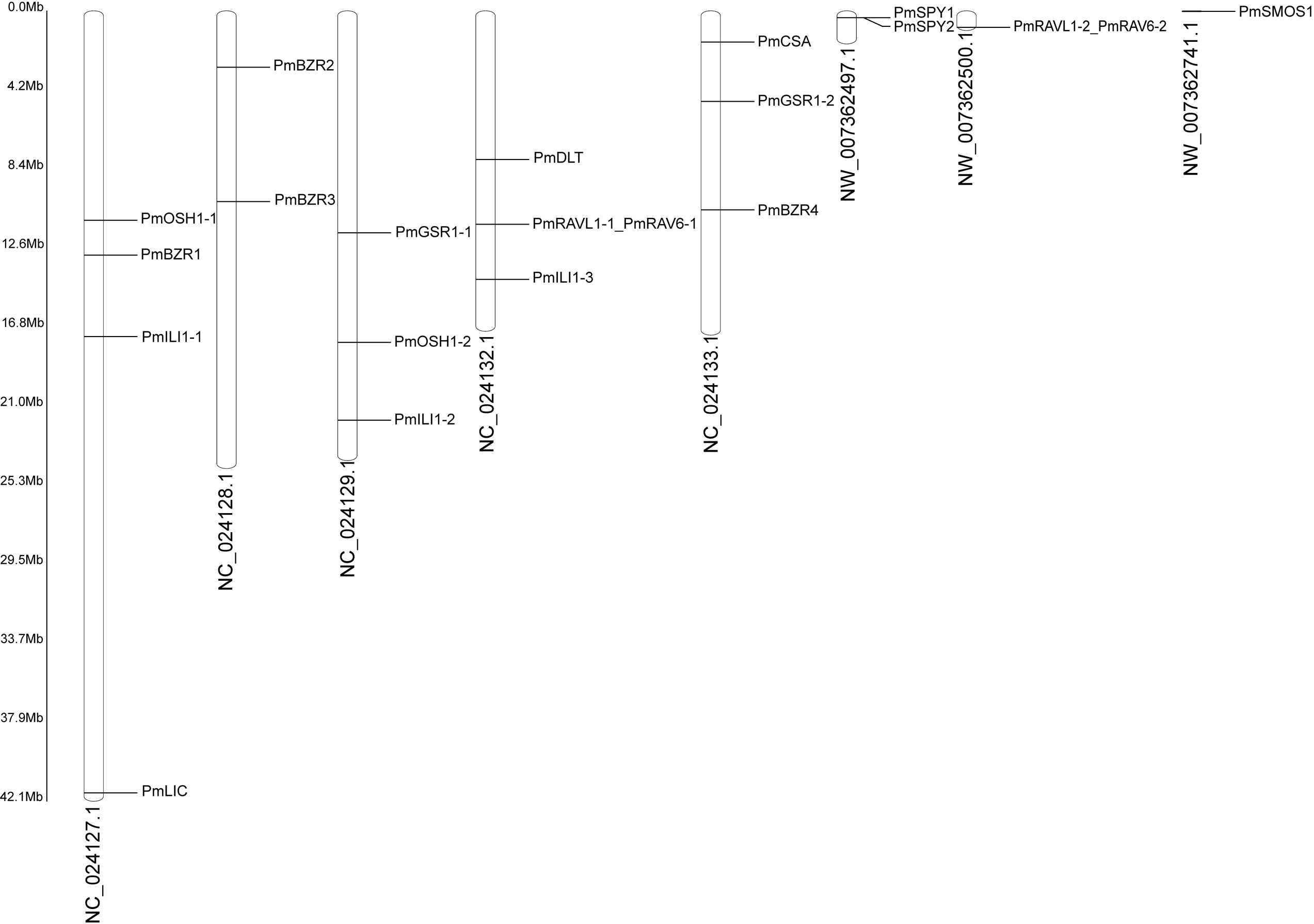

Supplement: Supplementary Figure 1 — Chromosomal location of BR downstream genes in nine Rosaceae species. [file Data_Sheet_1.docx]
