# Supplementary material for: Genome-Wide Identification of Brassinosteroid Signaling Downstream Genes in Nine Rosaceae Species and Analyses of Their Roles in Stem Growth and Stress Response in Apple
Source: Front Genet. 2021 Mar 18;12:640271. doi: 10.3389/fgene.2021.640271 (PMC8012692; doi:10.3389/fgene.2021.640271)

**Supplemental Figure 2 Transmembrane topology analysis of BR downstream proteins**

The red peaks indicate the predicted transmembrane helice.

**Supplemental Figure 2-1 Transmembrane topology analysis of BZRs proteins**


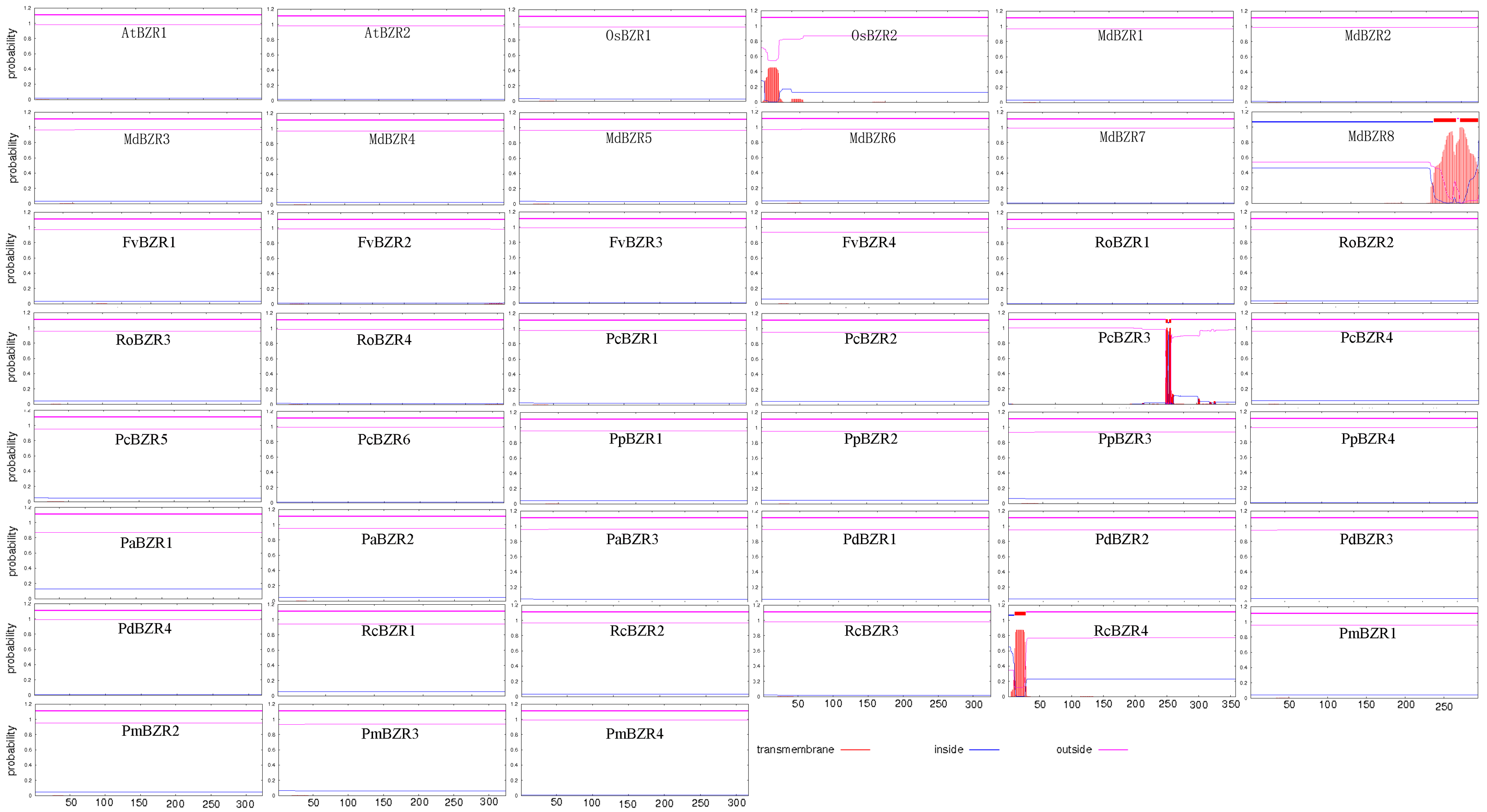


**Supplemental Figure 2-2 Transmembrane topology analysis of DLT proteins**


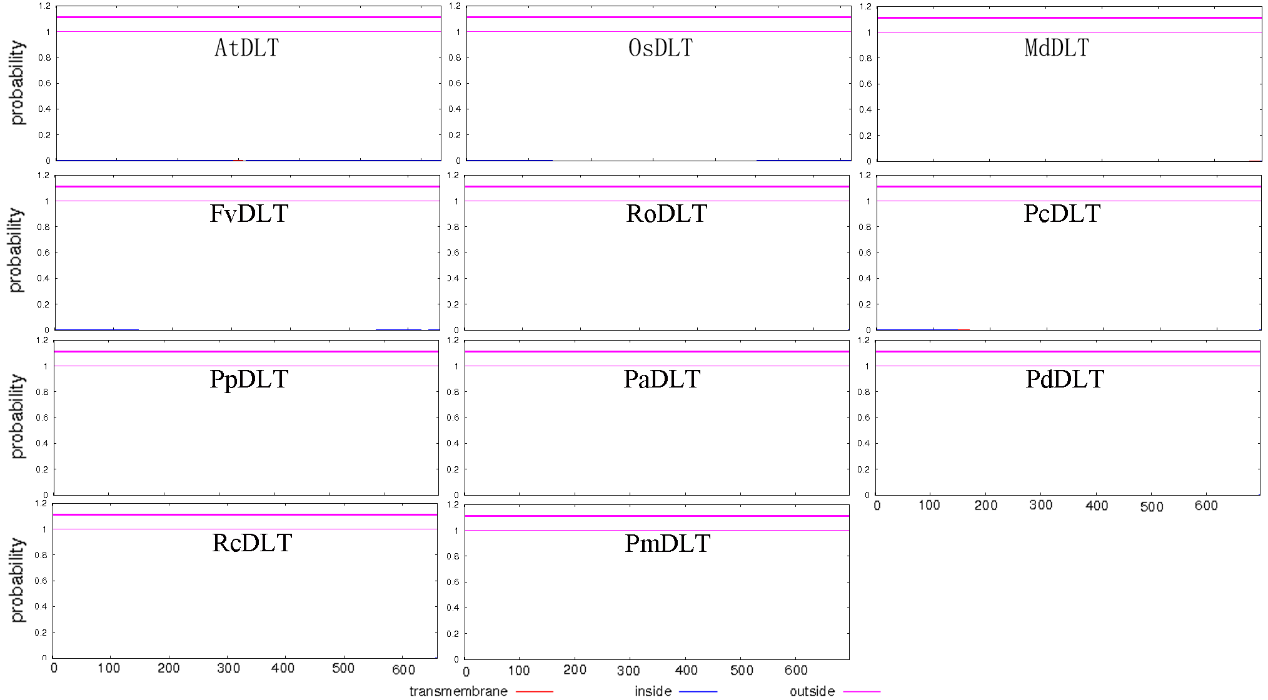


**Supplemental Figure 2-3 Transmembrane topology analysis of LIC proteins**


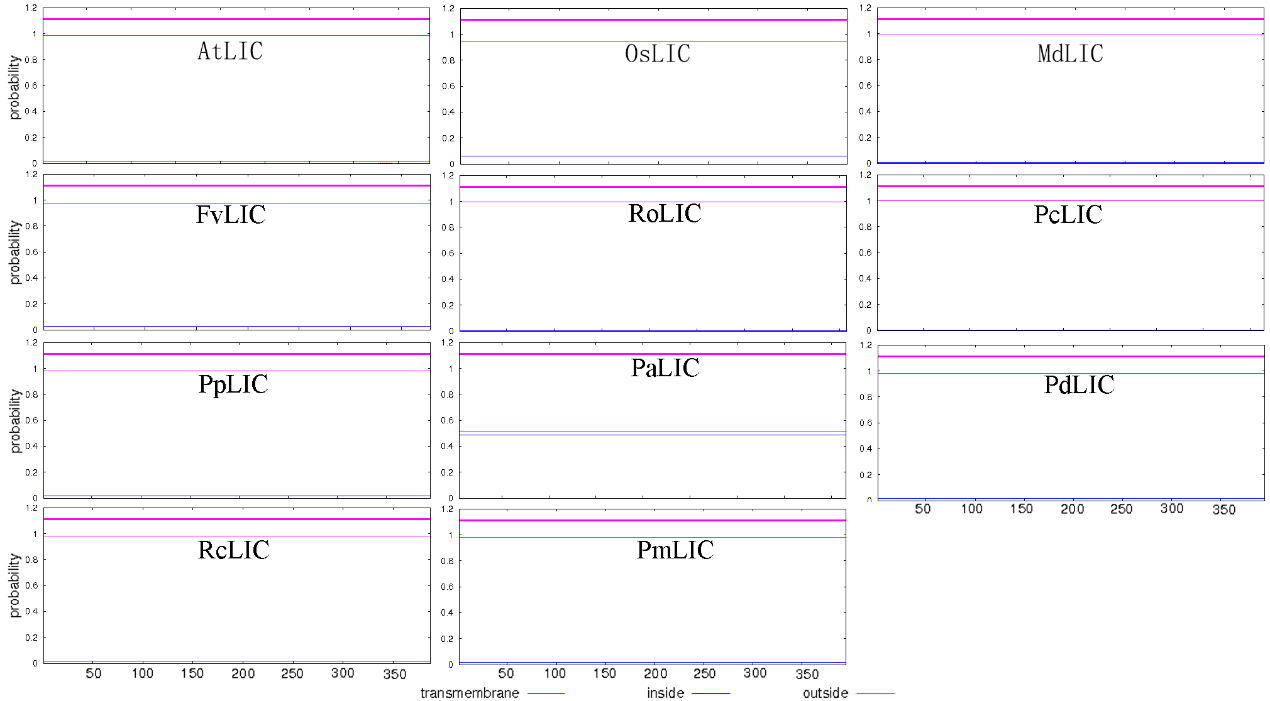


**Supplemental Figure 2-4 Transmembrane topology analysis of ILI1 proteins**


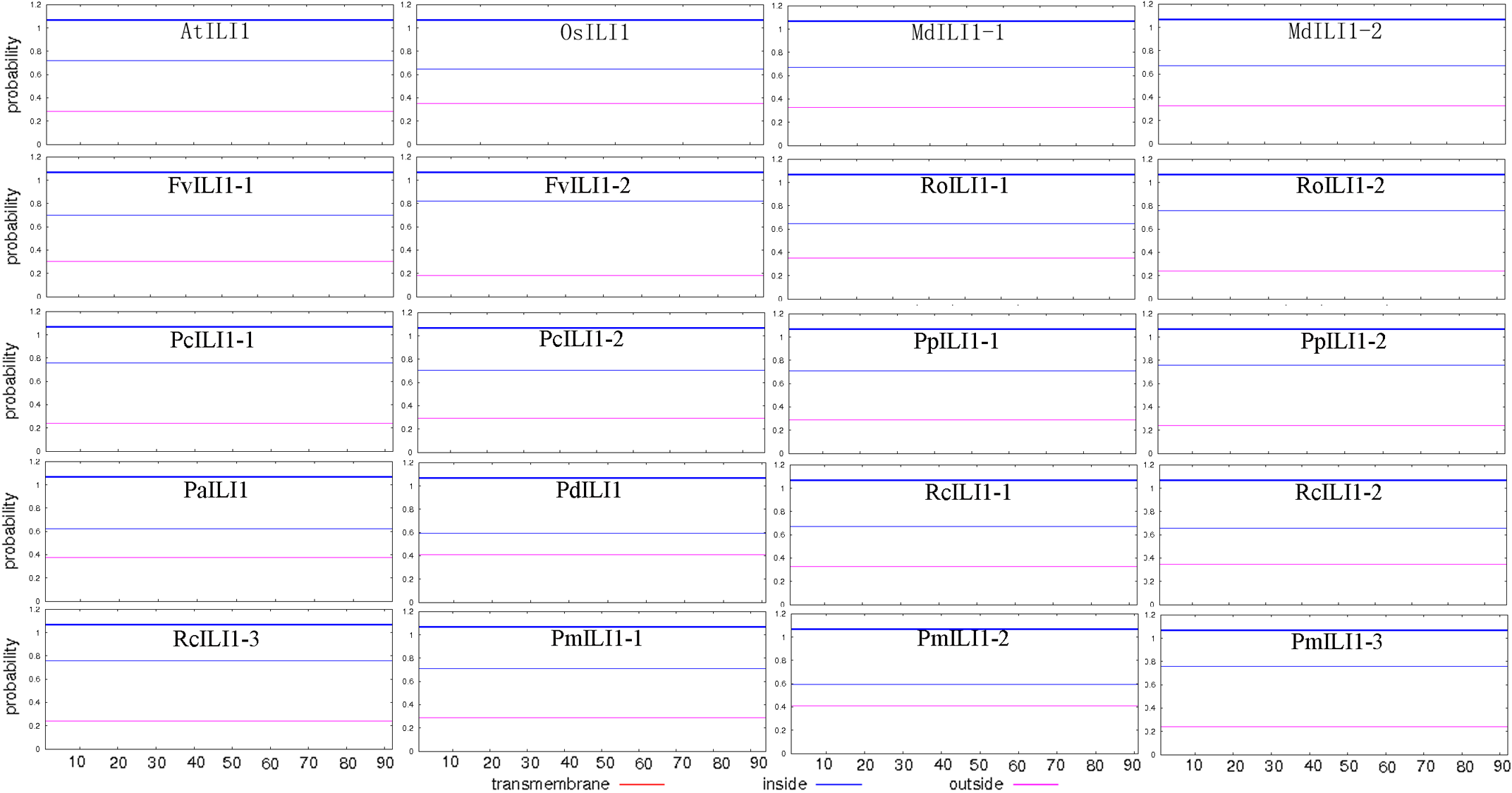


**Supplemental Figure 2-5 Transmembrane topology analysis of OSH1 proteins**


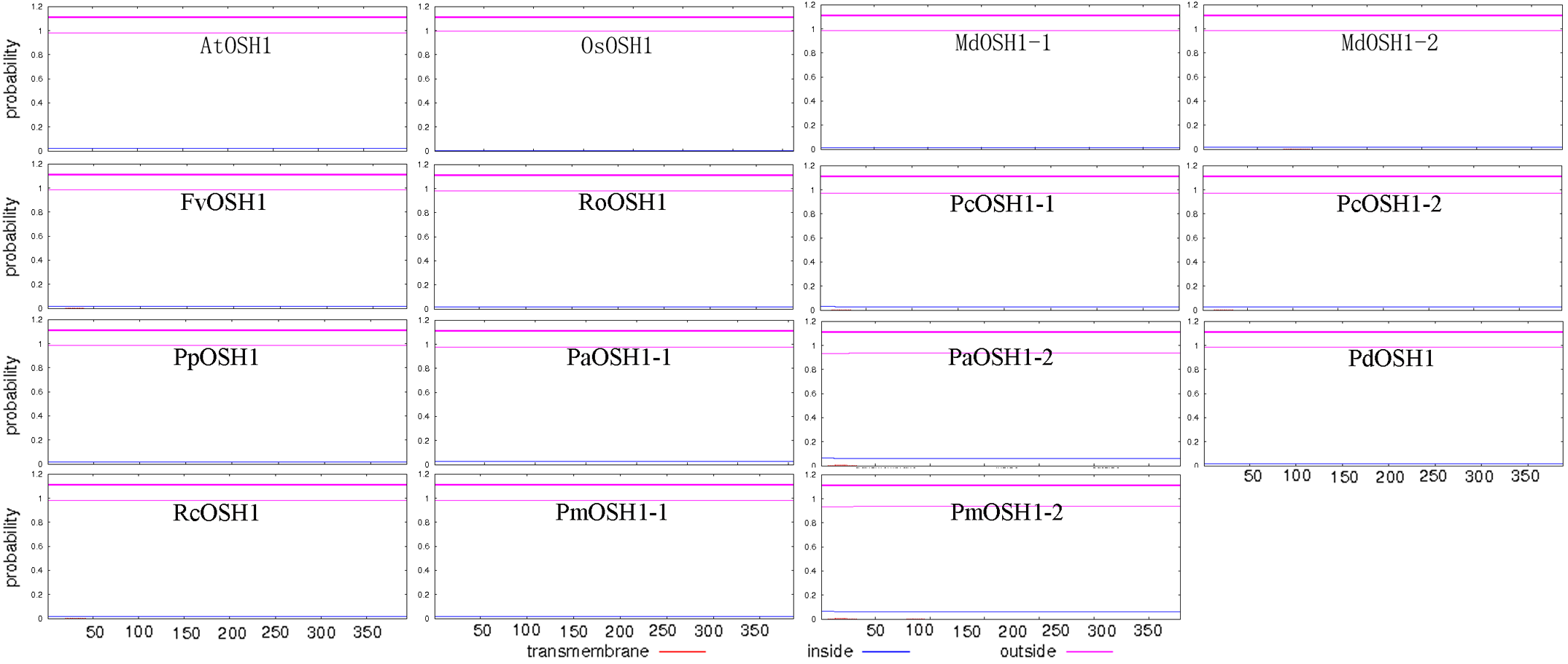


**Supplemental Figure 2-6 Transmembrane topology analysis of RAVL1/RAV6 proteins**


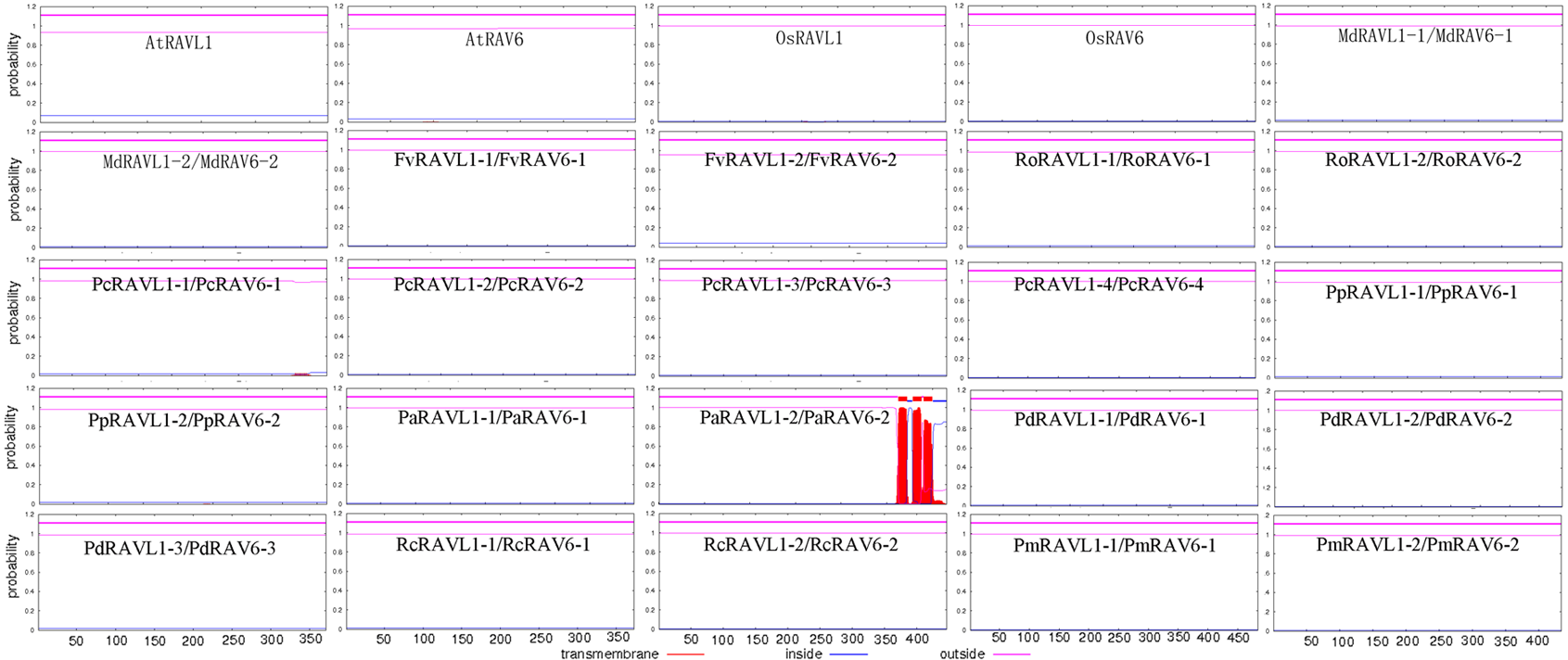


**Supplemental Figure 2-7 Transmembrane topology analysis of SMOS1 proteins**


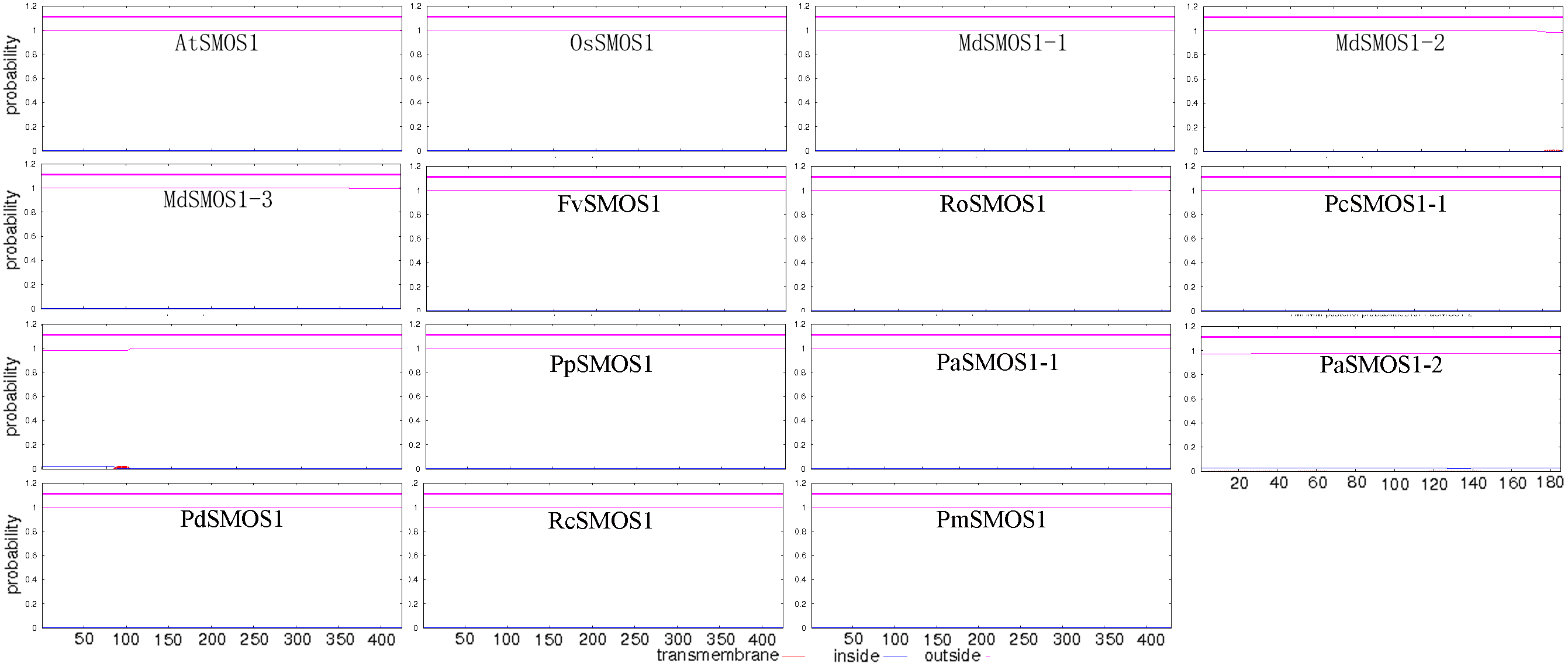


**Supplemental Figure 2-8 Transmembrane topology analysis of CSA proteins**


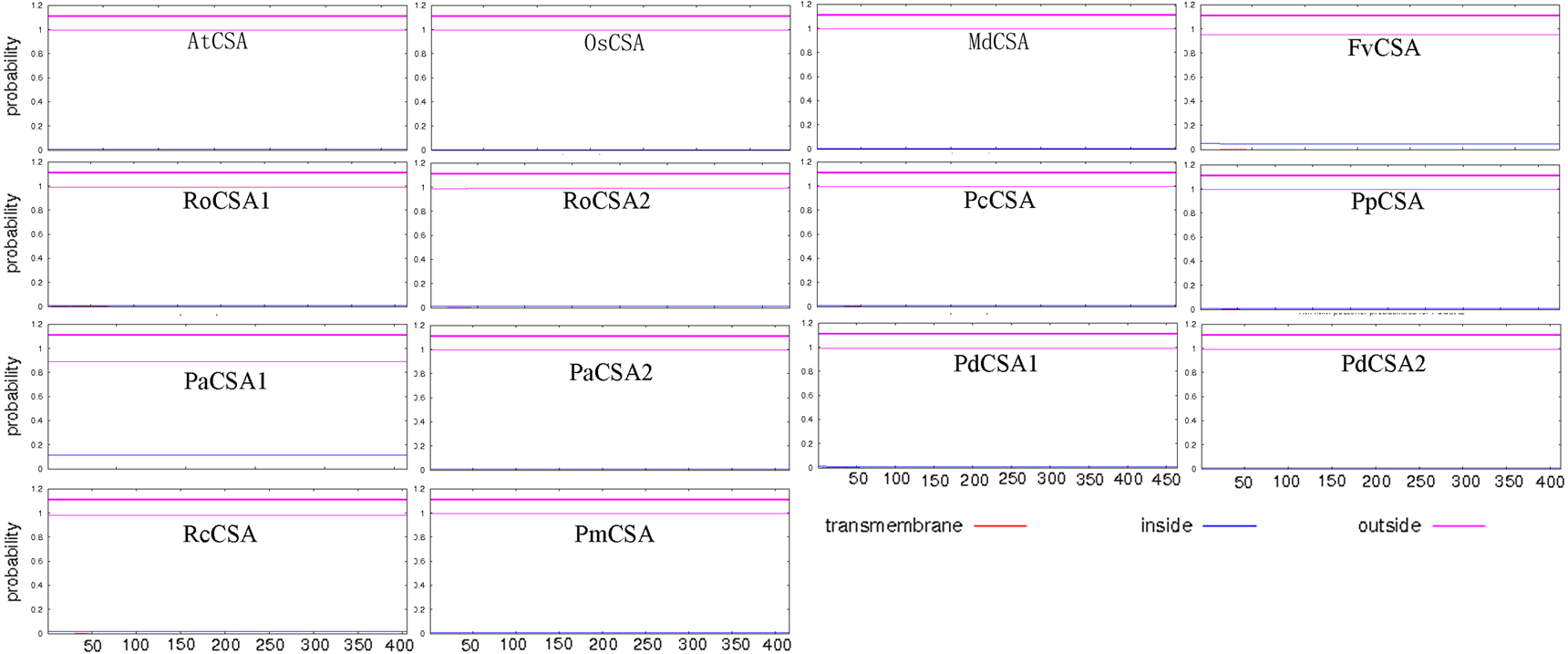


**Supplemental Figure 2-9 Transmembrane topology analysis of SPY proteins**


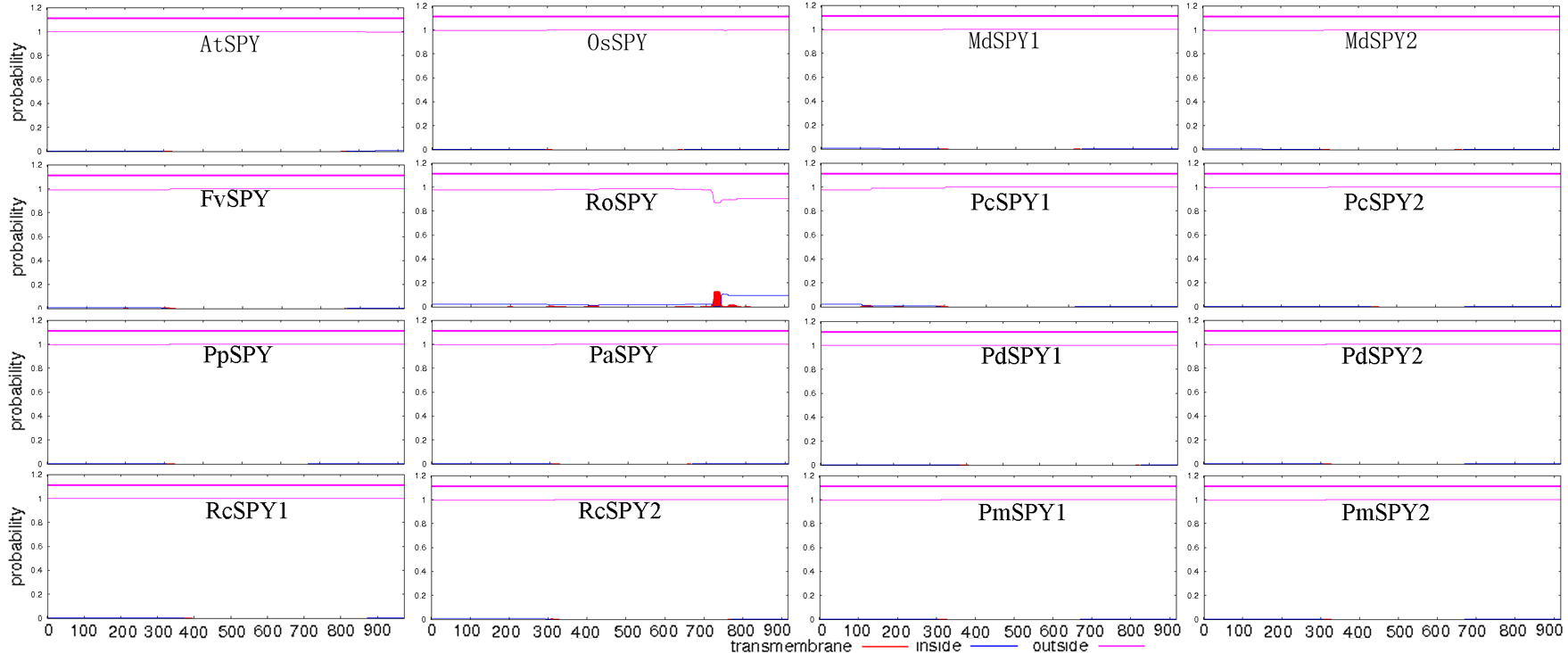


**Supplemental Figure 2-10 Transmembrane topology analysis of GSR1 proteins**


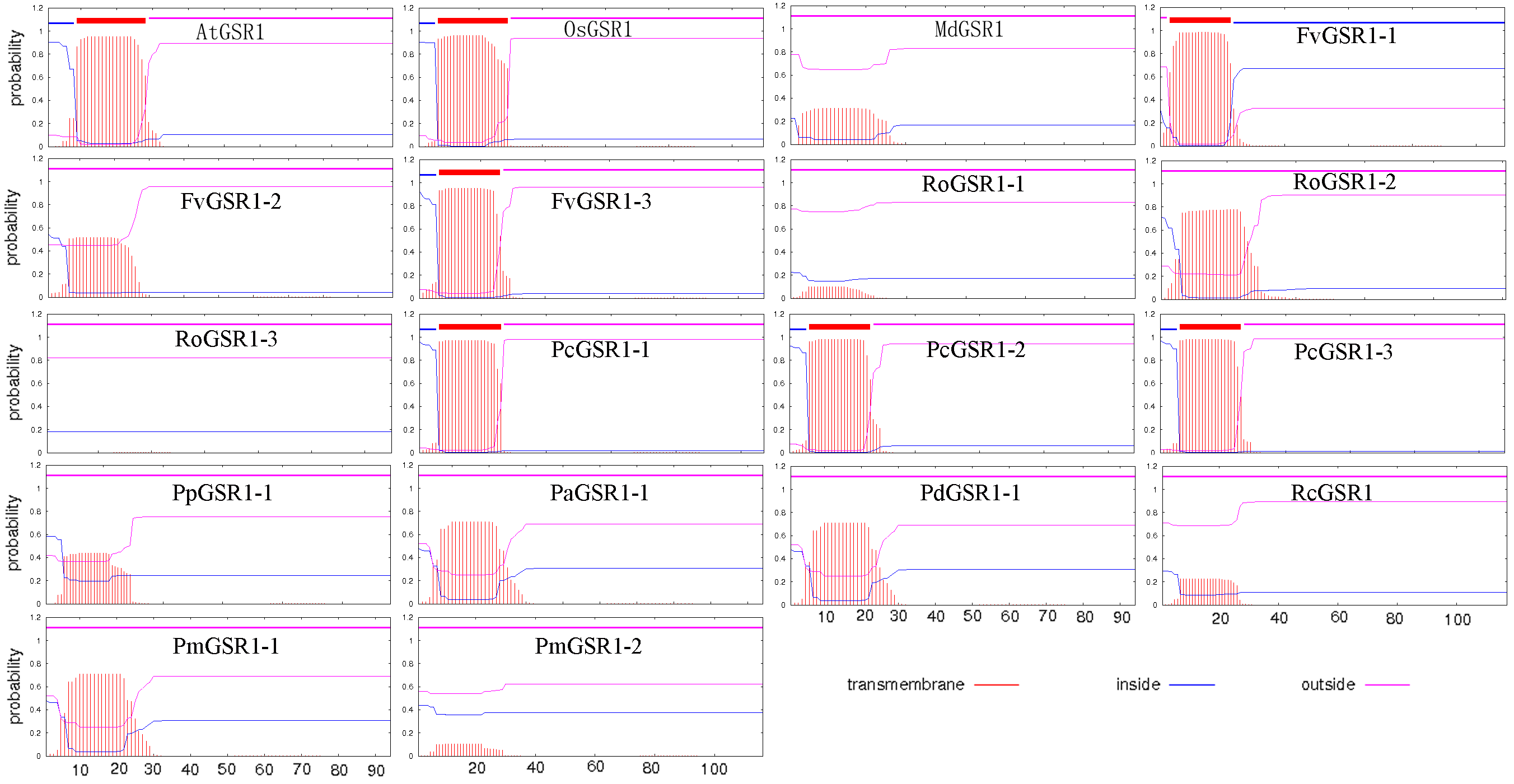

Supplement: Supplementary Figure 2 — Transmembrane topology analysis of BR downstream proteins. Red peaks indicate predicted transmembrane helices. [file Data_Sheet_2.docx]
