# Supplementary material for: Genome-Wide Identification of Brassinosteroid Signaling Downstream Genes in Nine Rosaceae Species and Analyses of Their Roles in Stem Growth and Stress Response in Apple
Source: Front Genet. 2021 Mar 18;12:640271. doi: 10.3389/fgene.2021.640271 (PMC8012692; doi:10.3389/fgene.2021.640271)

**Supplemental Figure 3. Synteny analysis of BR downstream genes in each rosaceae specie**

**Supplemental Figure 3-1 Synteny analysis of BR downstream genes in *Malus domestica***


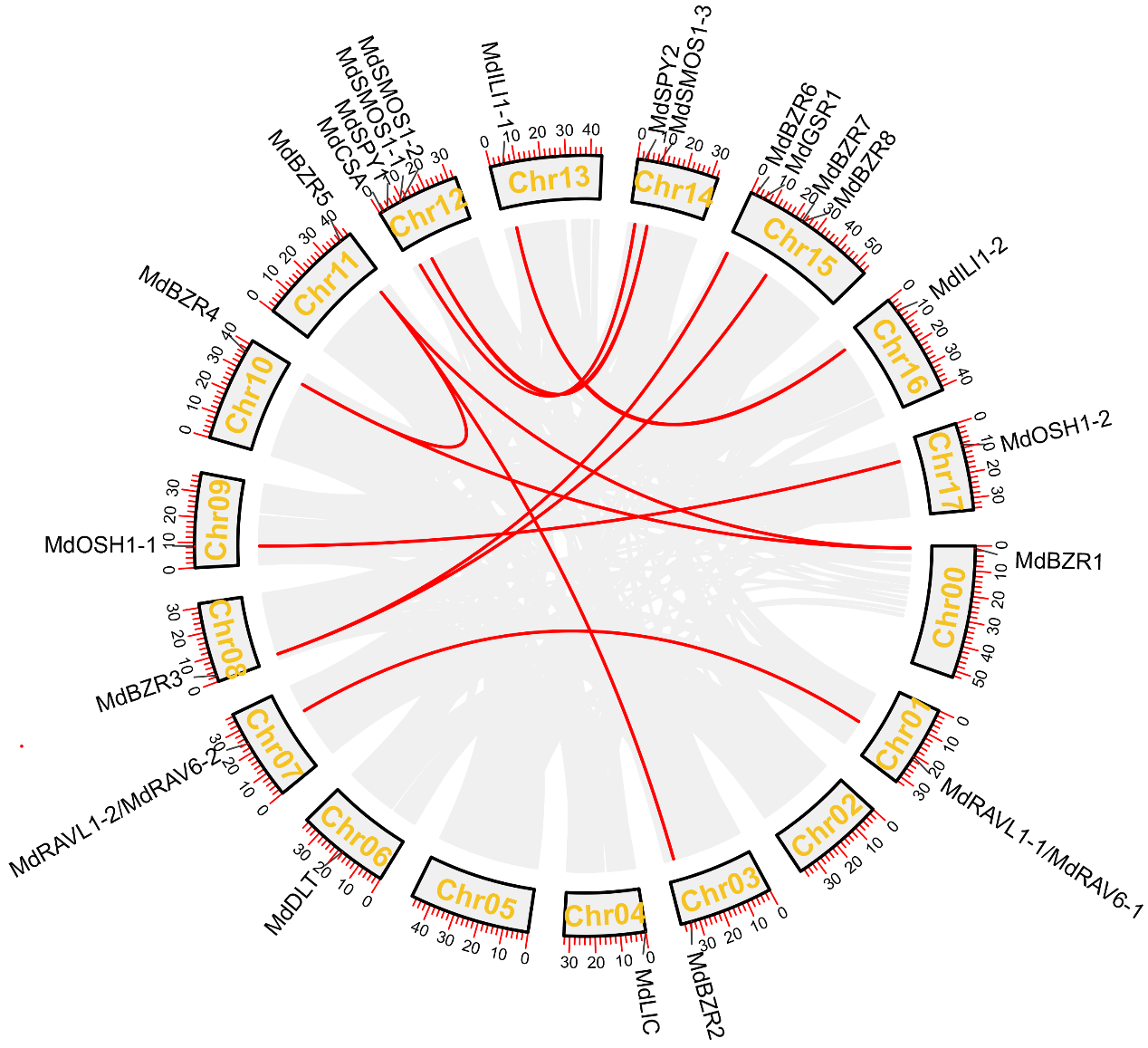


**Supplemental Figure 3-2 Synteny analysis of BR downstream genes in *Fragaria vesca***


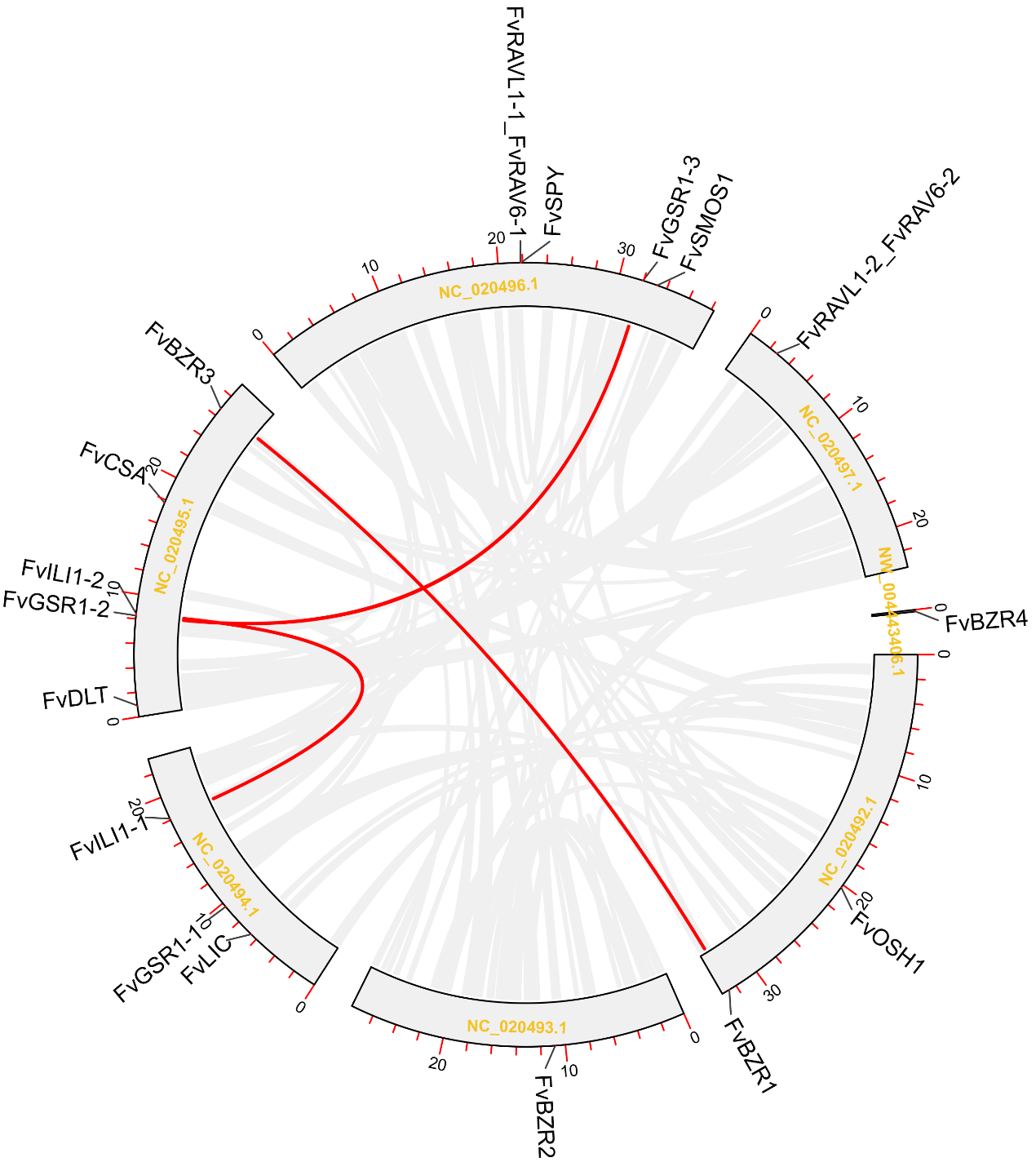


**Supplemental Figure 3-3 Synteny analysis of BR downstream genes in *Rubus occidentalis***


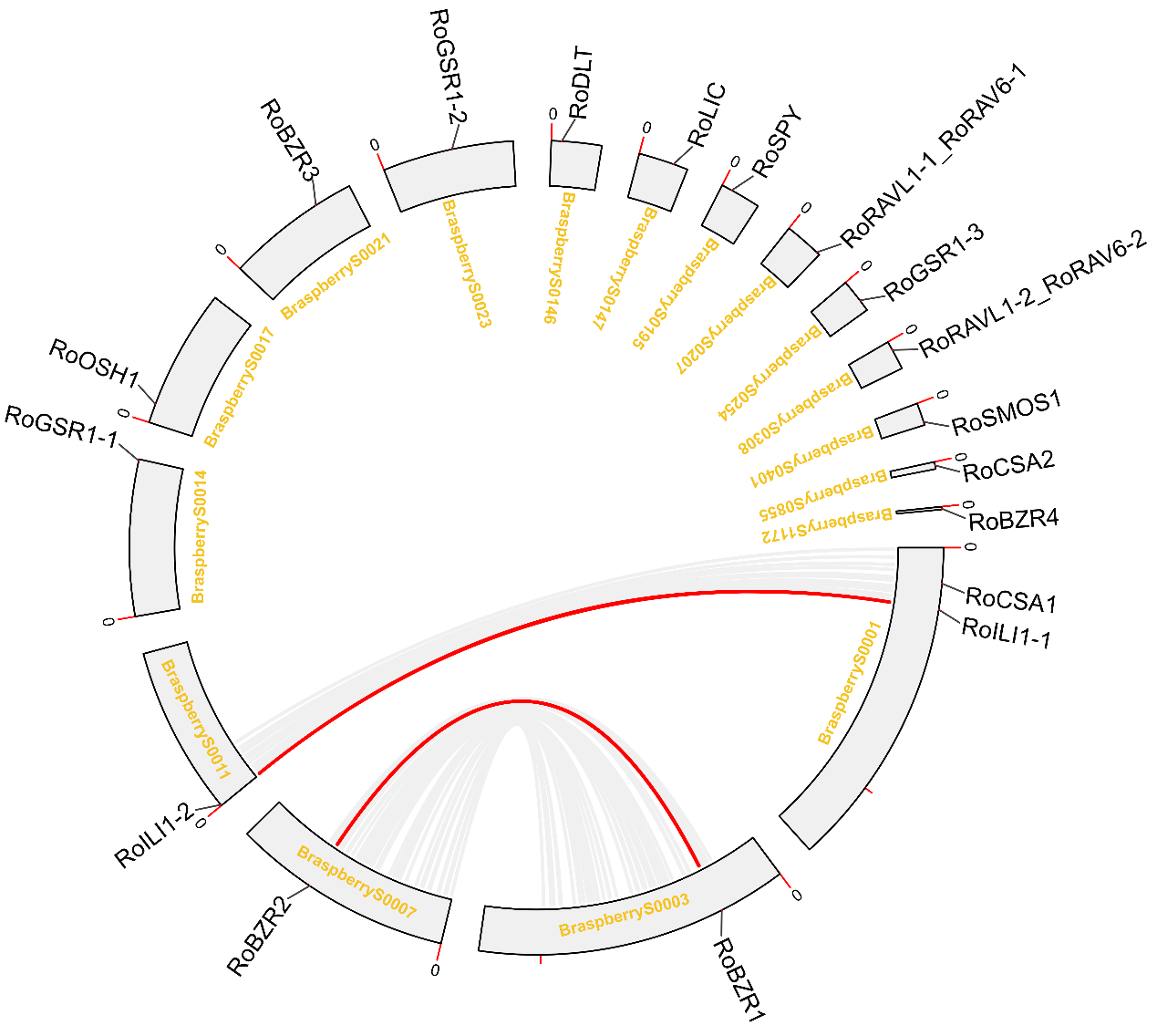


**Supplemental Figure 3-4 Synteny analysis of BR downstream genes in *Pyrus communis***


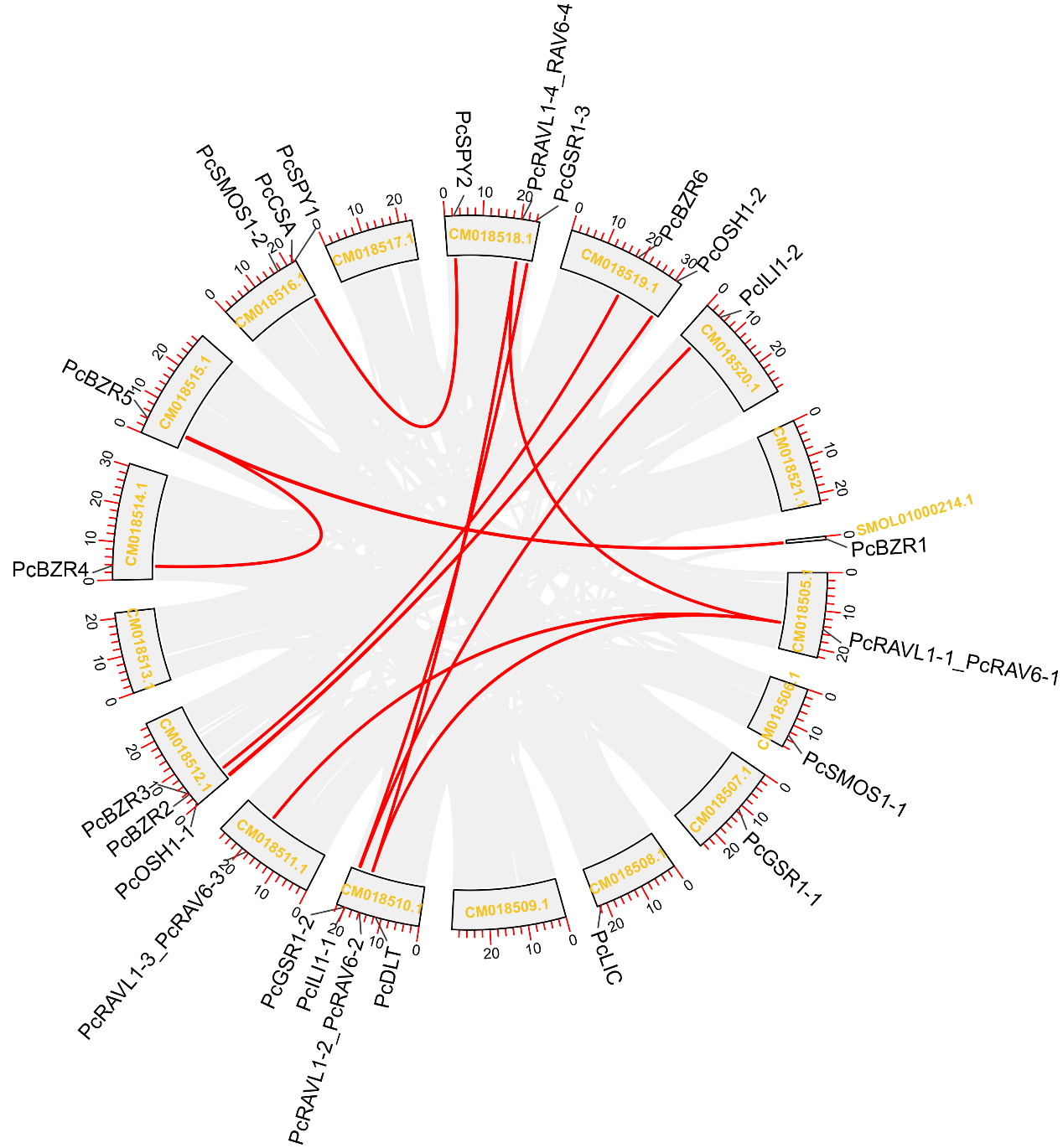


**Supplemental Figure 3-5 Synteny analysis of BR downstream genes in *Prunus persica***


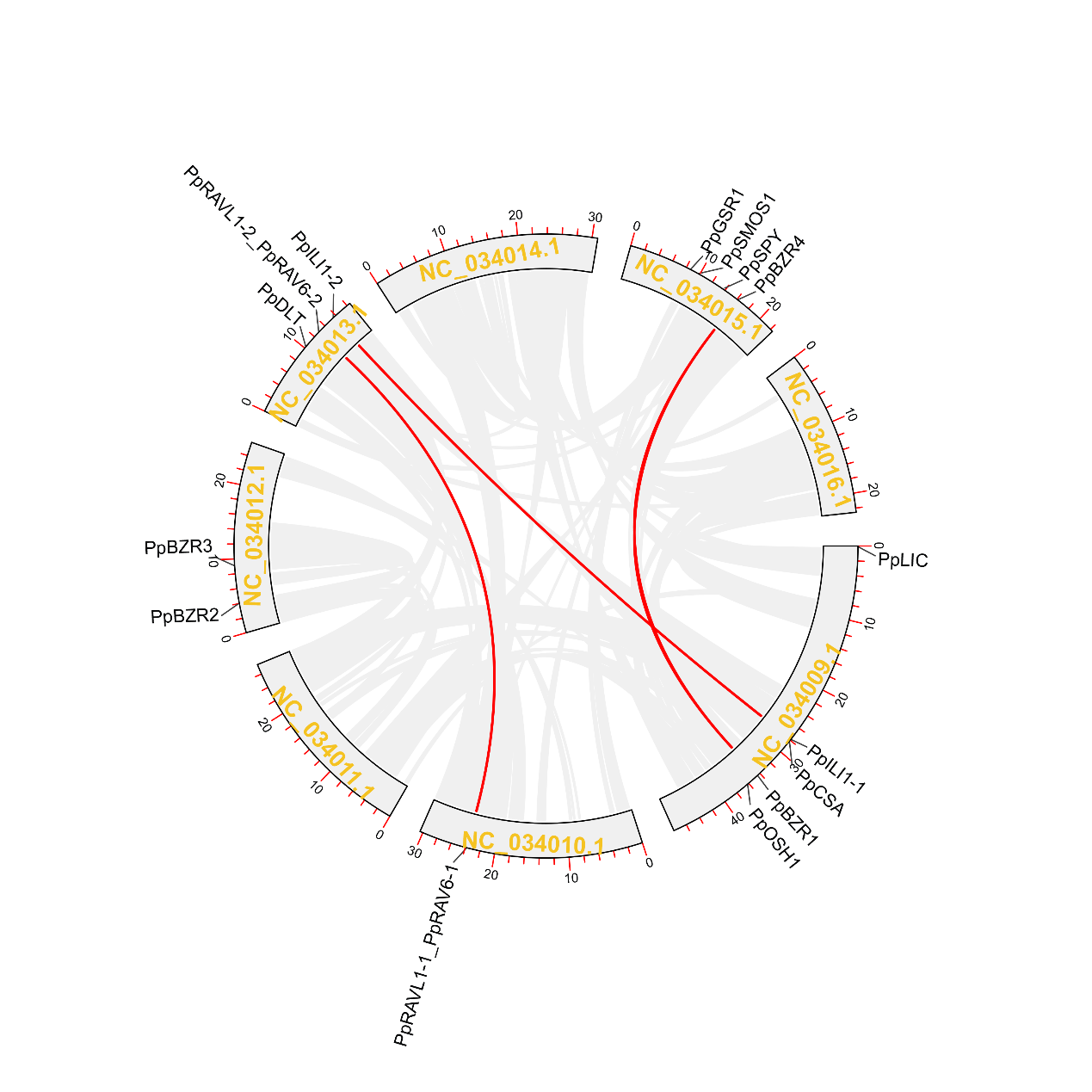


**Supplemental Figure 3-6 Synteny analysis of BR downstream genes in *Prunus avium***


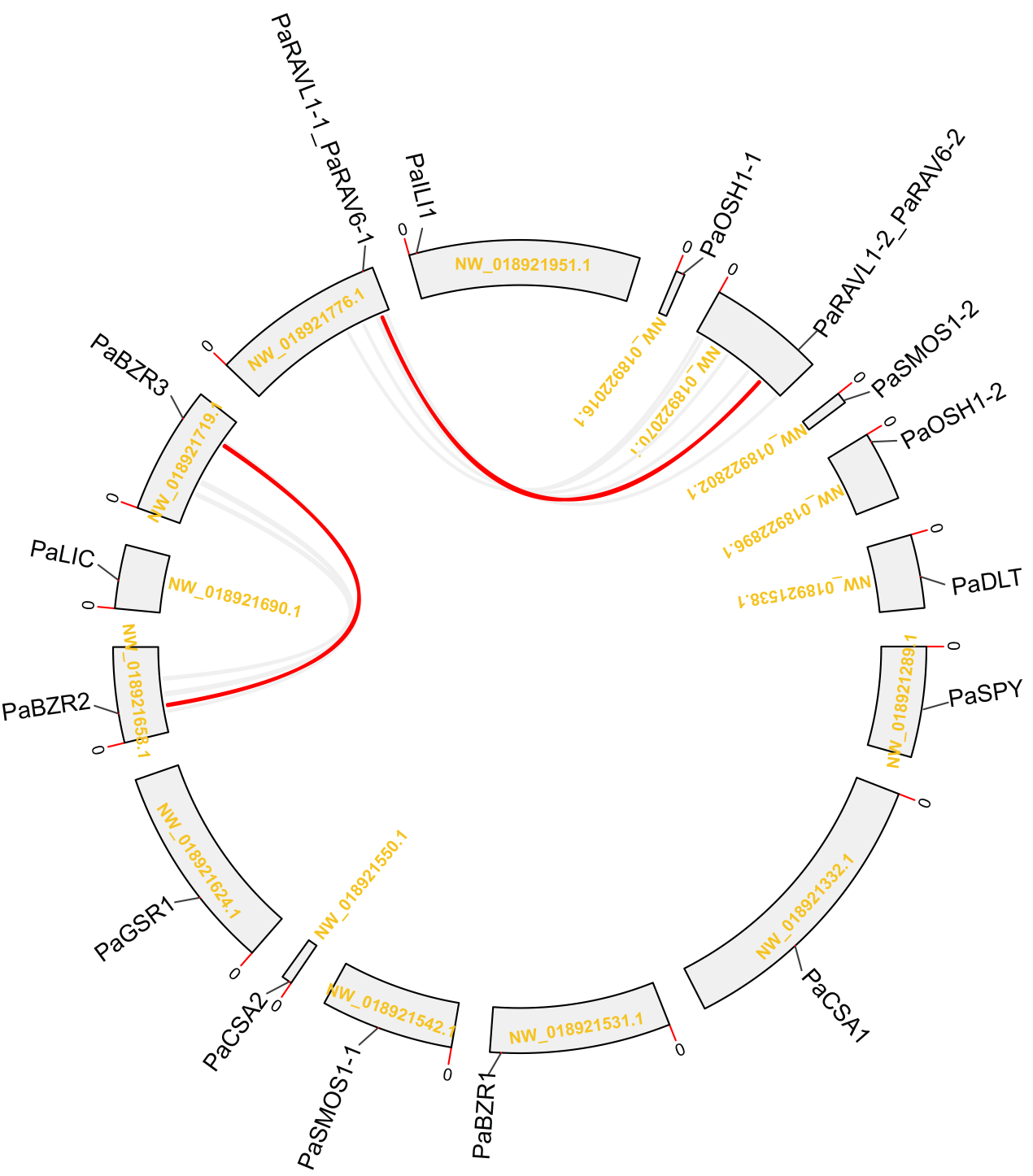


**Supplemental Figure 3-7 Synteny analysis of BR downstream genes in *Prunus dulcis***


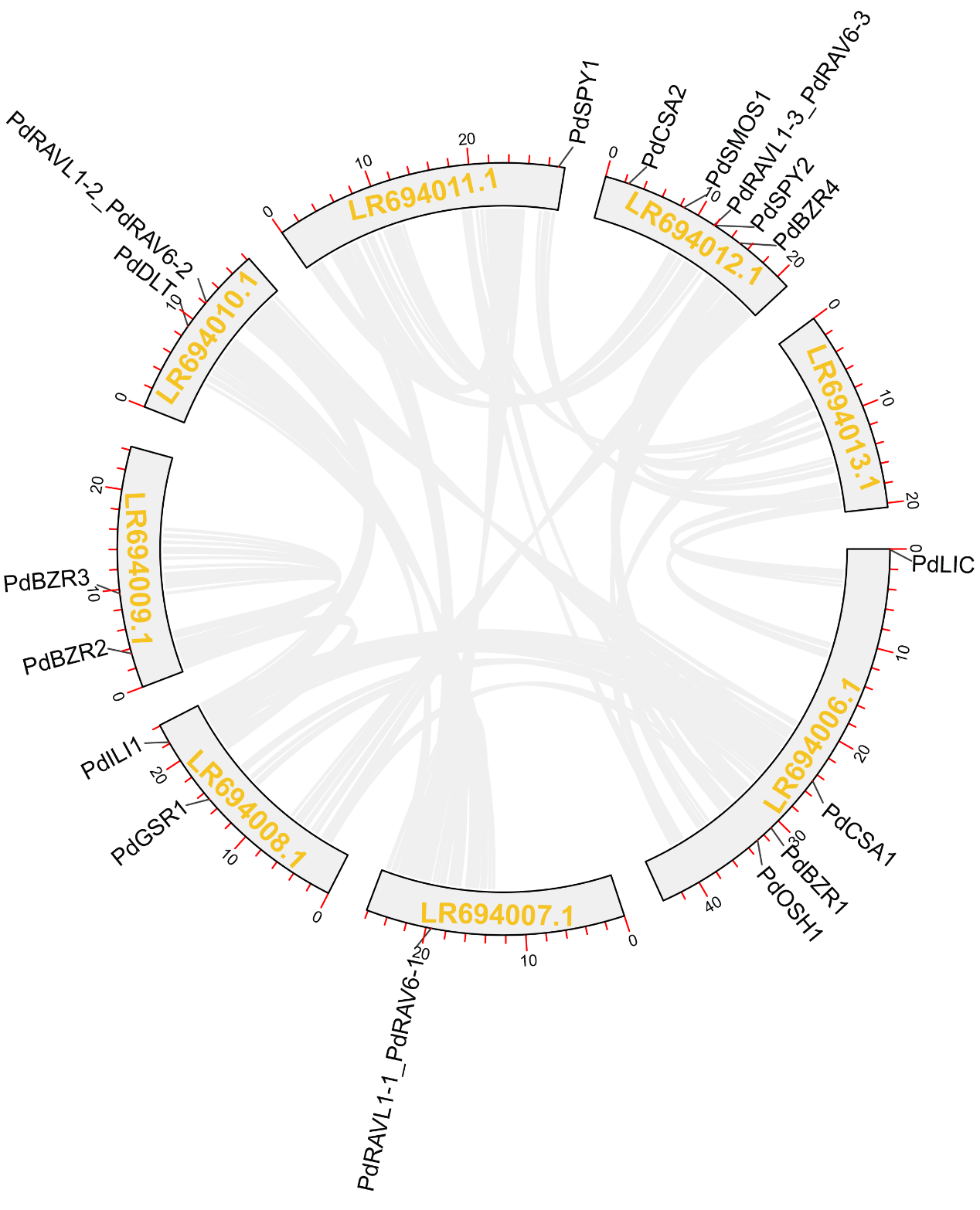


**Supplemental Figure 3-8 Synteny analysis of BR downstream genes in *Rosa chinensis***


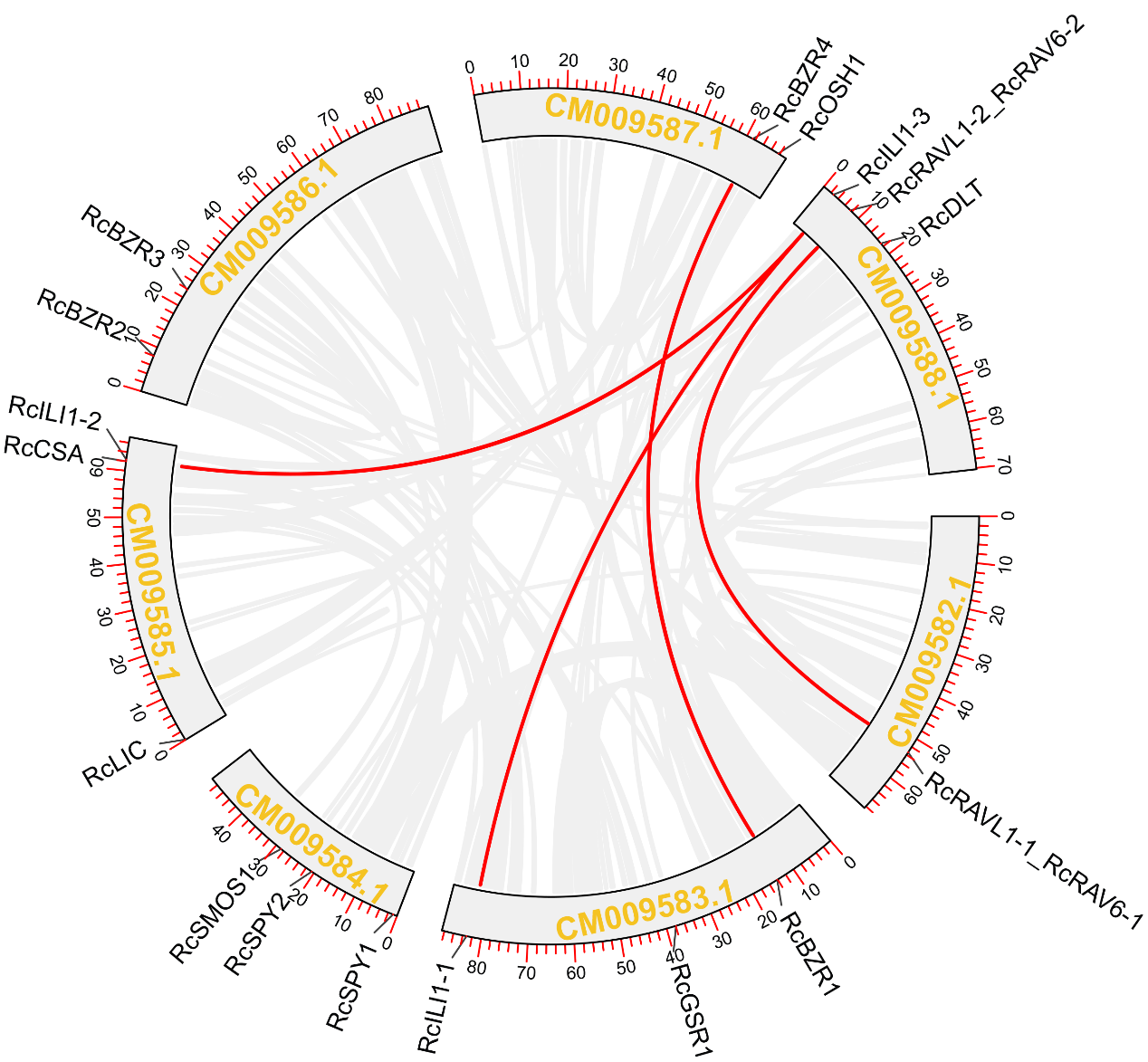


**Supplemental Figure 3-9 Synteny analysis of BR downstream genes in *Prunus mume***


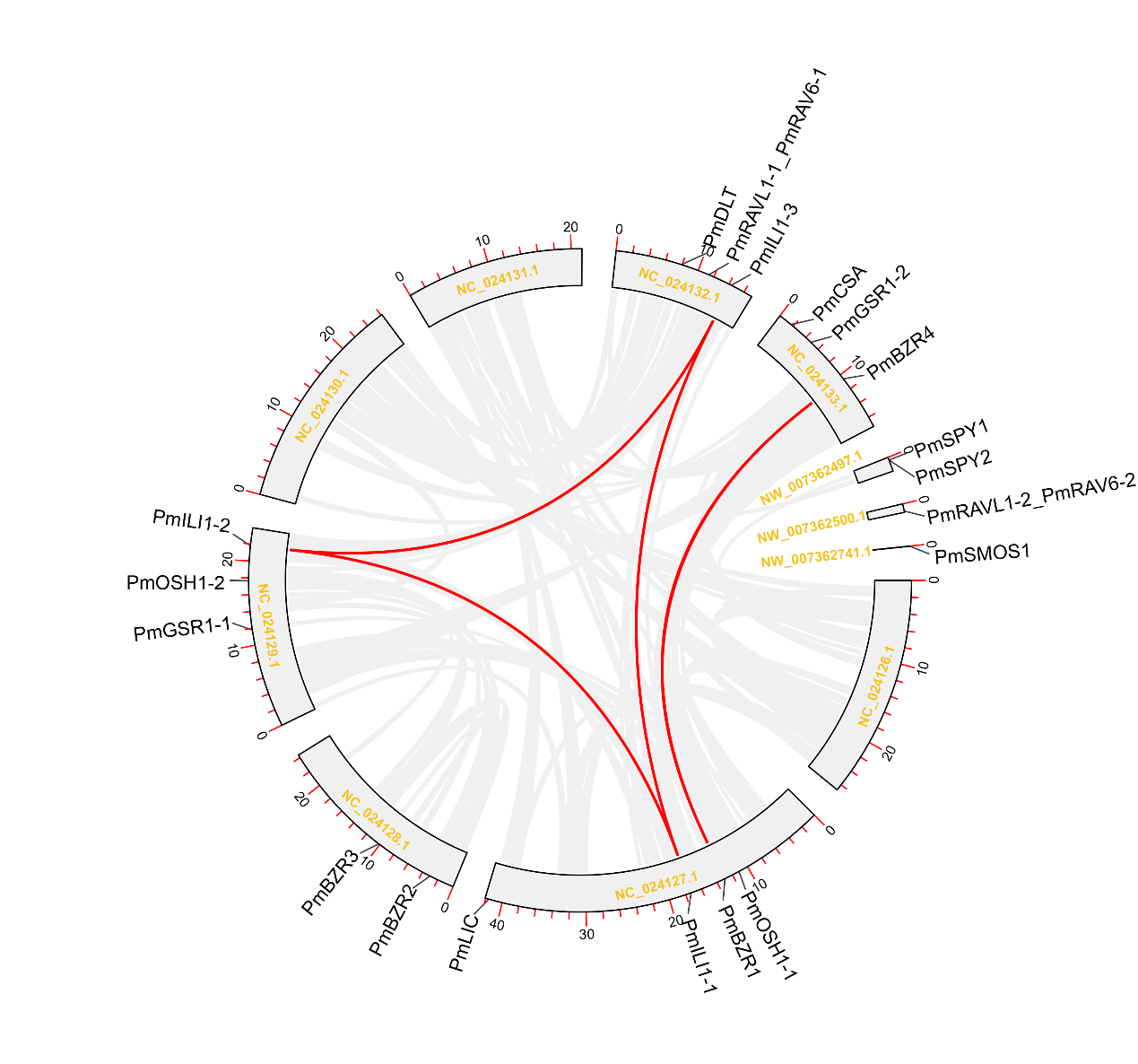

Supplement: Supplementary Figure 3 — Synteny analysis of BR downstream genes in each Rosaceae species. [file Data_Sheet_3.docx]
