# Supplementary material for: Genome-Wide Identification of Brassinosteroid Signaling Downstream Genes in Nine Rosaceae Species and Analyses of Their Roles in Stem Growth and Stress Response in Apple
Source: Front Genet. 2021 Mar 18;12:640271. doi: 10.3389/fgene.2021.640271 (PMC8012692; doi:10.3389/fgene.2021.640271)

**Supplemental Figure 4. Analysis of the synteny among the BR downstream genes of *Oryza sativa* and nine rosaceae species**

**Supplemental Figure 4-1. Analysis of the synteny among the BR downstream genes of *Oryza sativa* and *Malus domestica***


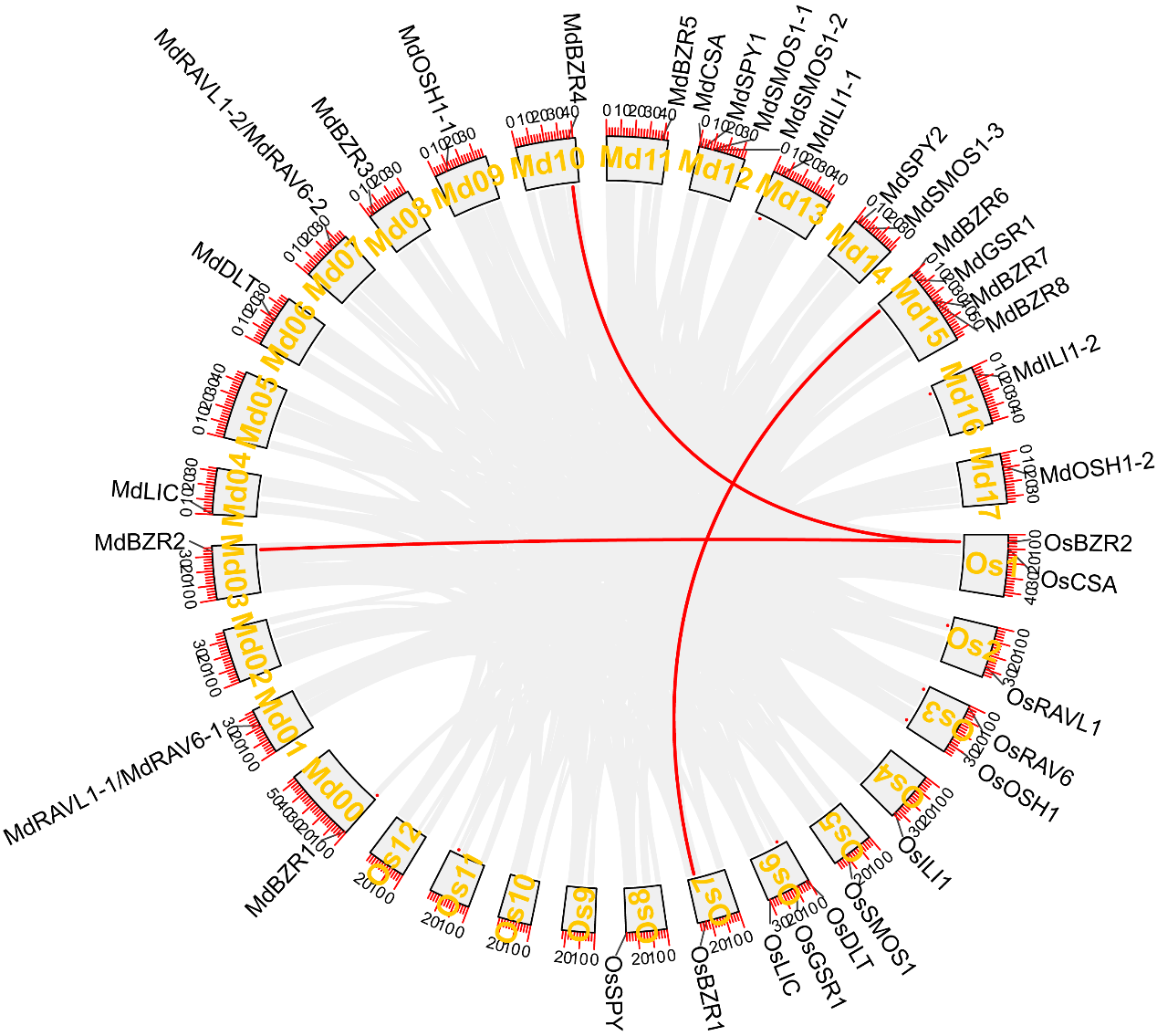


**Supplemental Figure 4-2. Analysis of the synteny among the BR downstream genes of *Oryza sativa* and *Fragaria vesca***


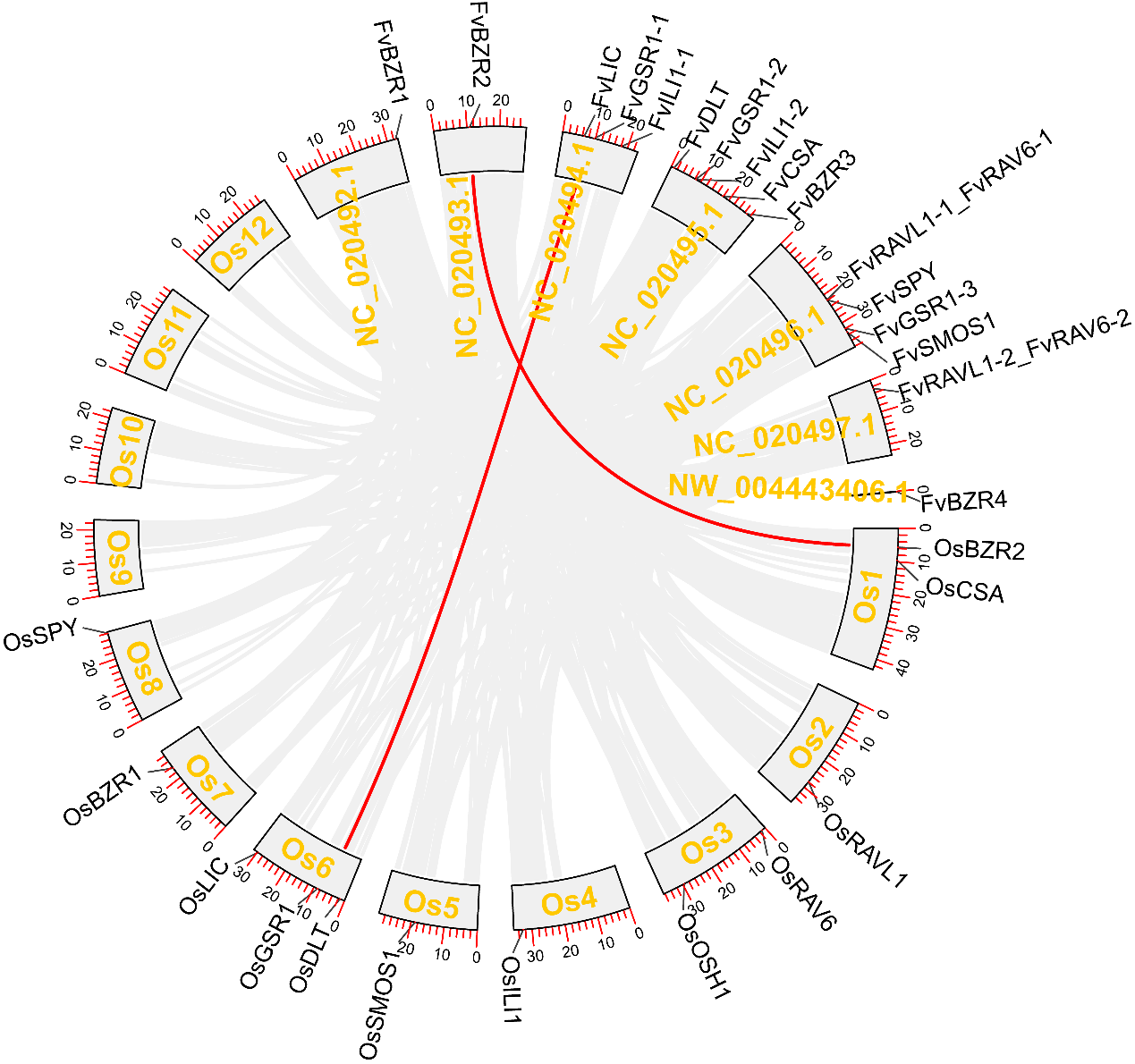


**Supplemental Figure 4-3. Analysis of the synteny among the BR downstream genes of *Oryza sativa* and *Rubus occidentalis***


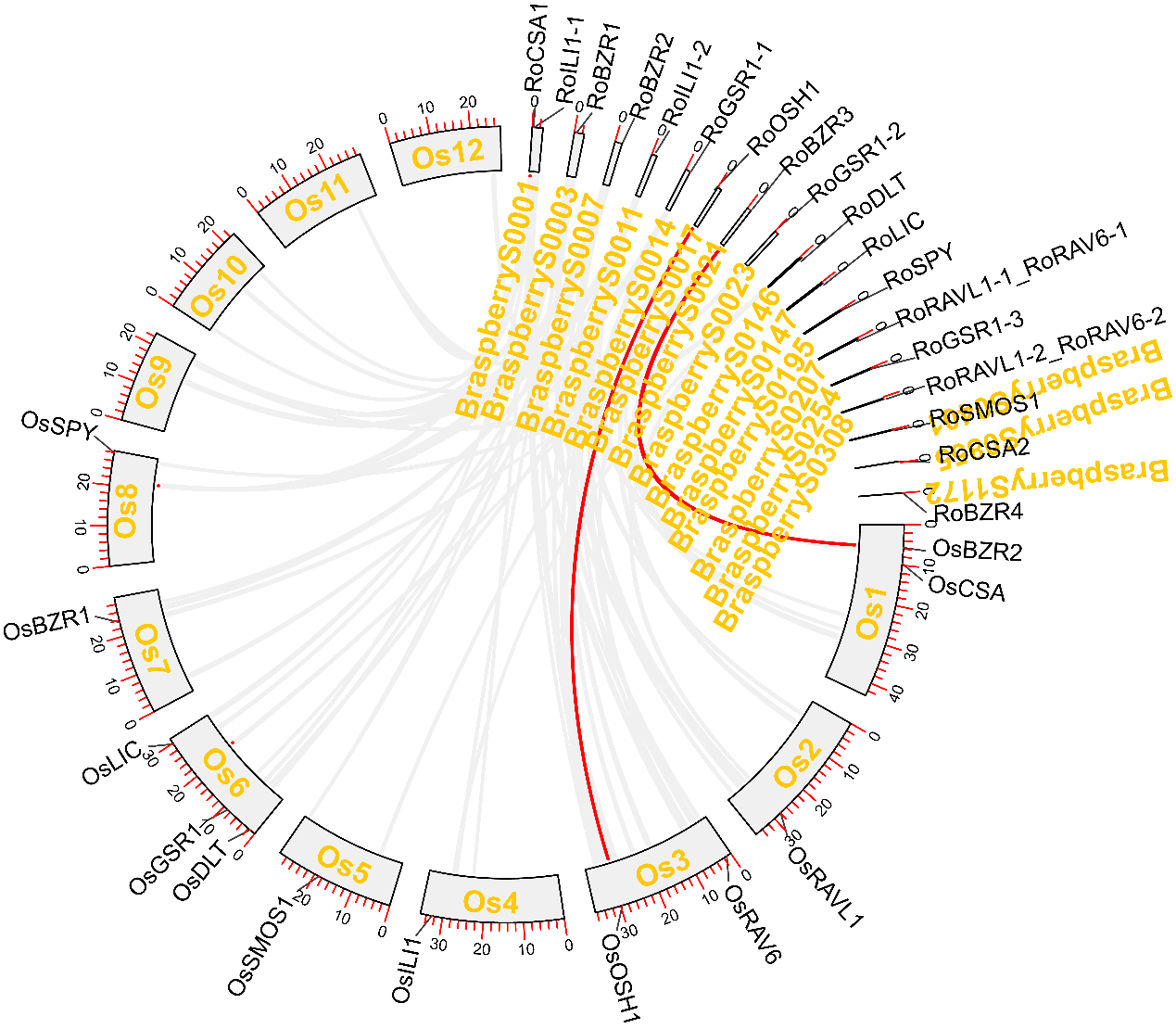


**Supplemental Figure 4-4. Analysis of the synteny among the BR downstream genes of *Oryza sativa* and *Pyrus communis***


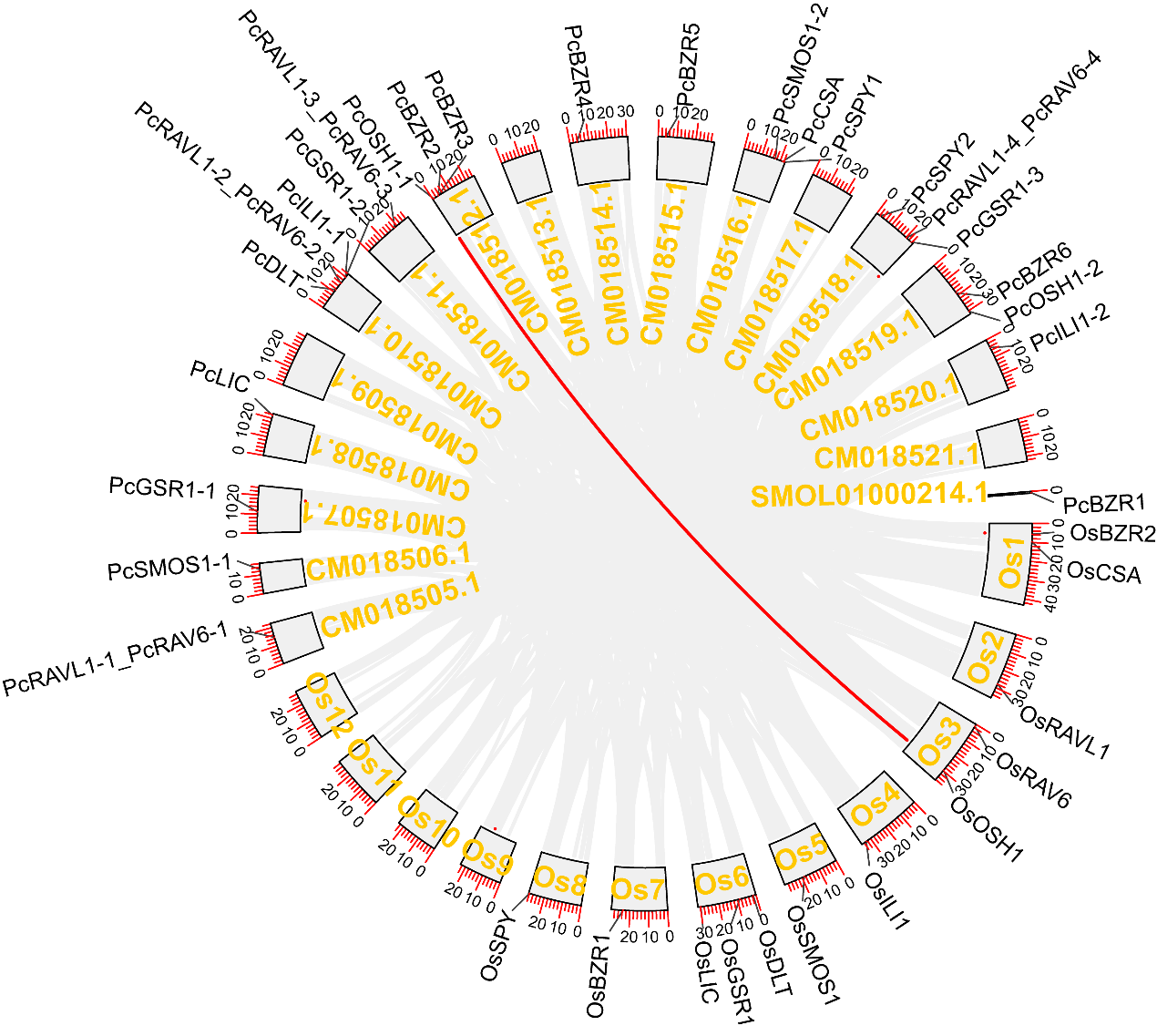


**Supplemental Figure 4-5. Analysis of the synteny among the BR downstream genes of *Oryza sativa* and *Prunus persica***


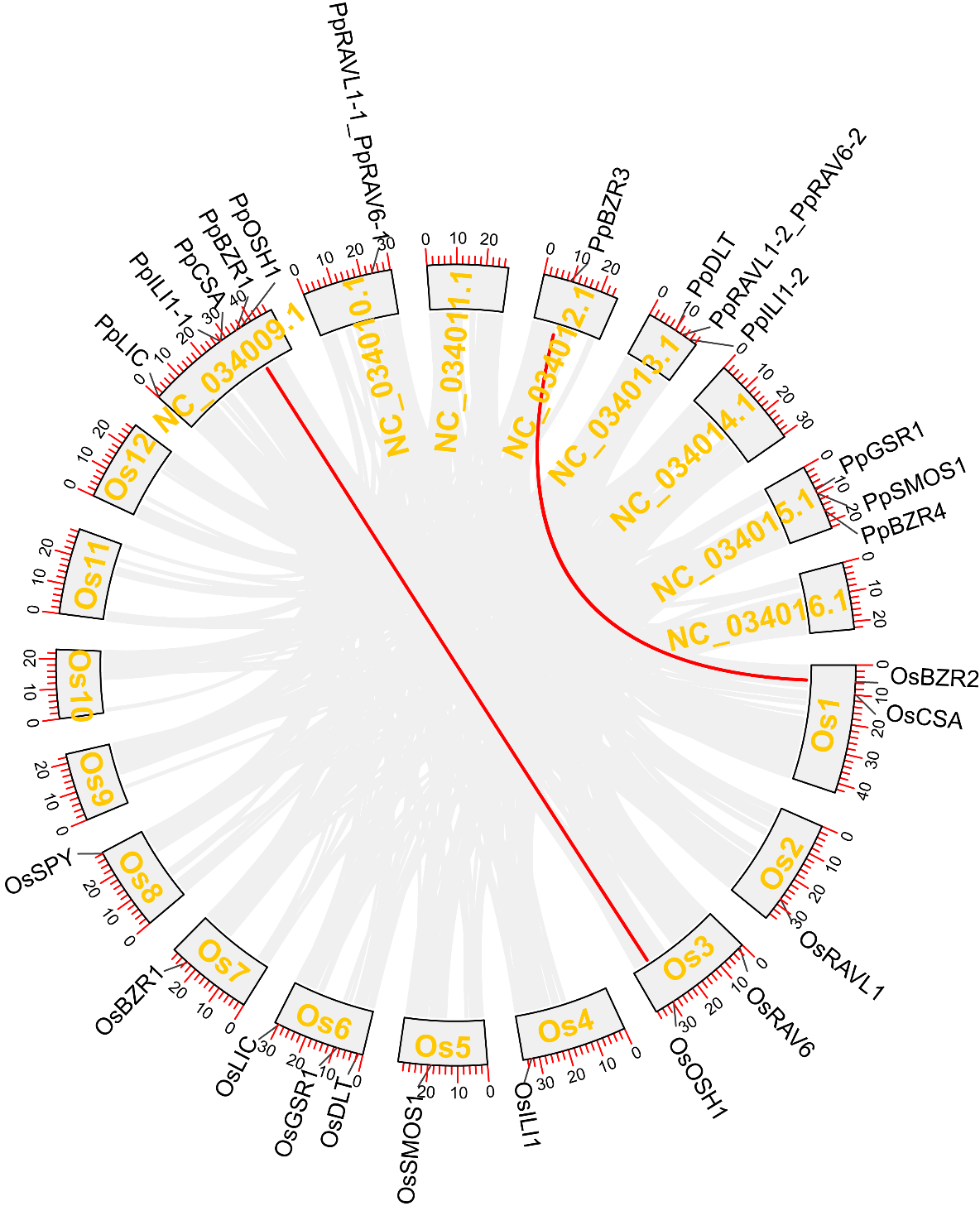


**Supplemental Figure 4-6. Analysis of the synteny among the BR downstream genes of *Oryza sativa* and *Prunus avium***


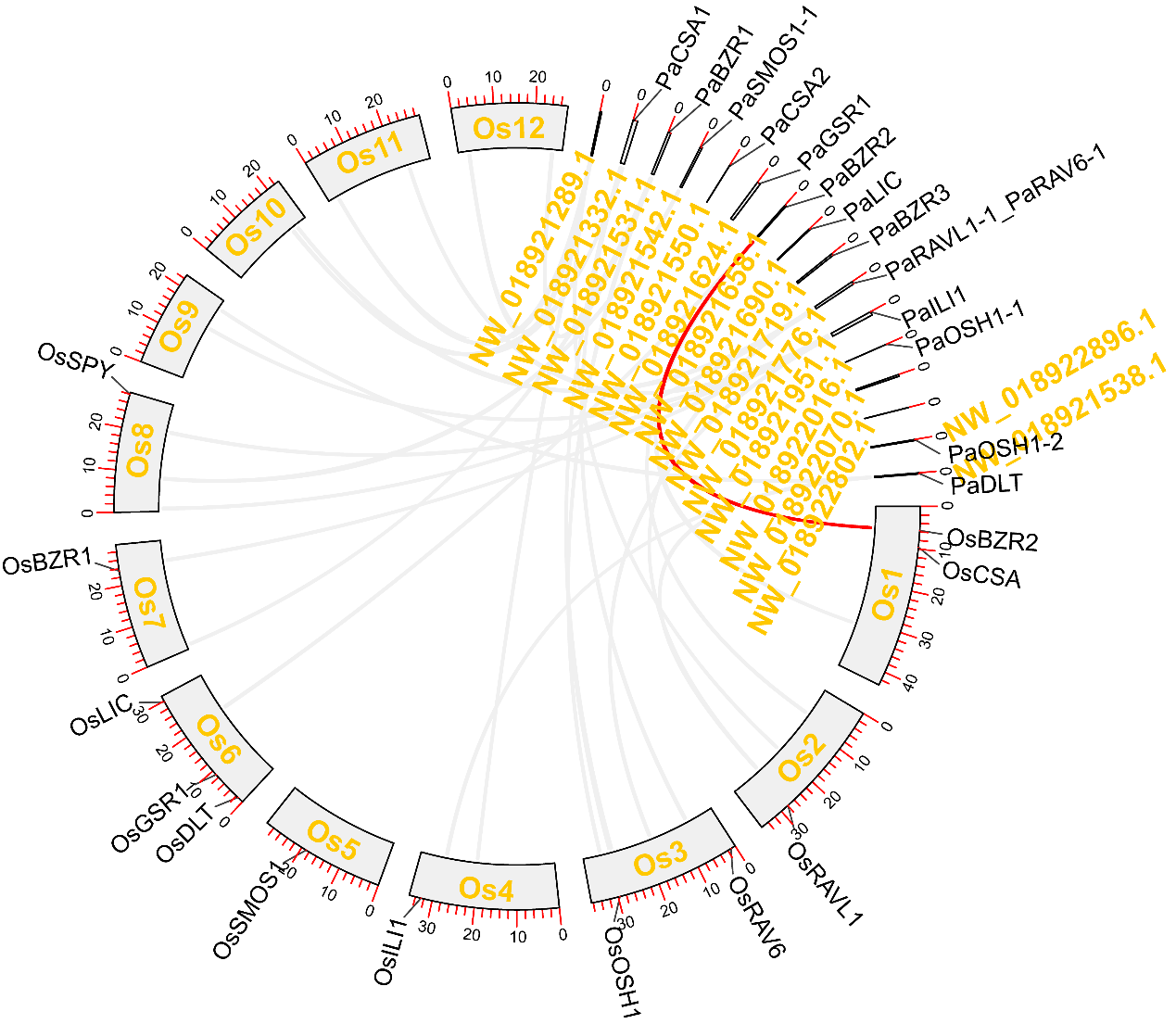


**Supplemental Figure 4-7. Analysis of the synteny among the BR downstream genes of *Oryza sativa* and *Prunus dulcis***


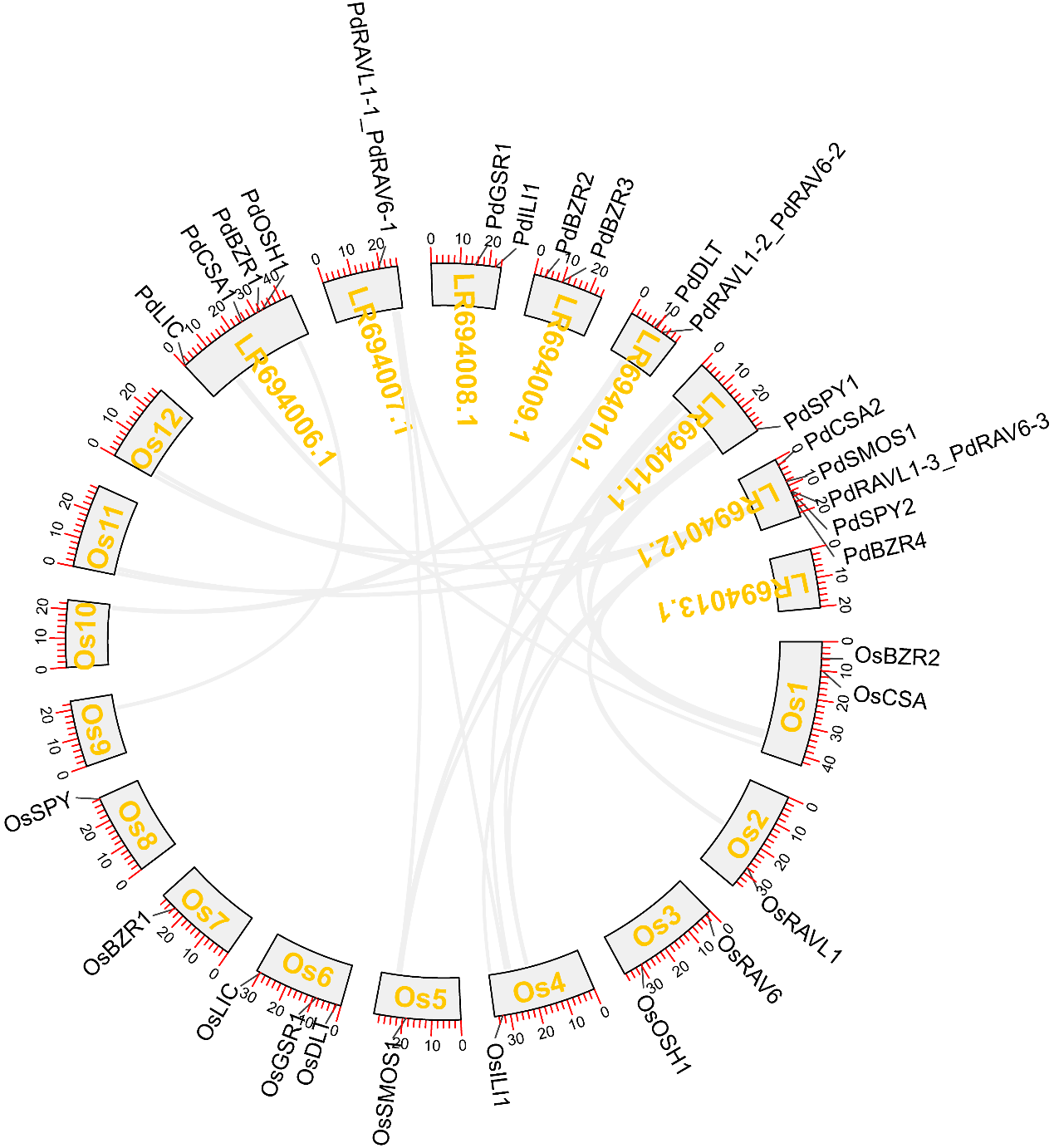


**Supplemental Figure 4-8. Analysis of the synteny among the BR downstream genes of *Oryza sativa* and *Rosa chinensis***


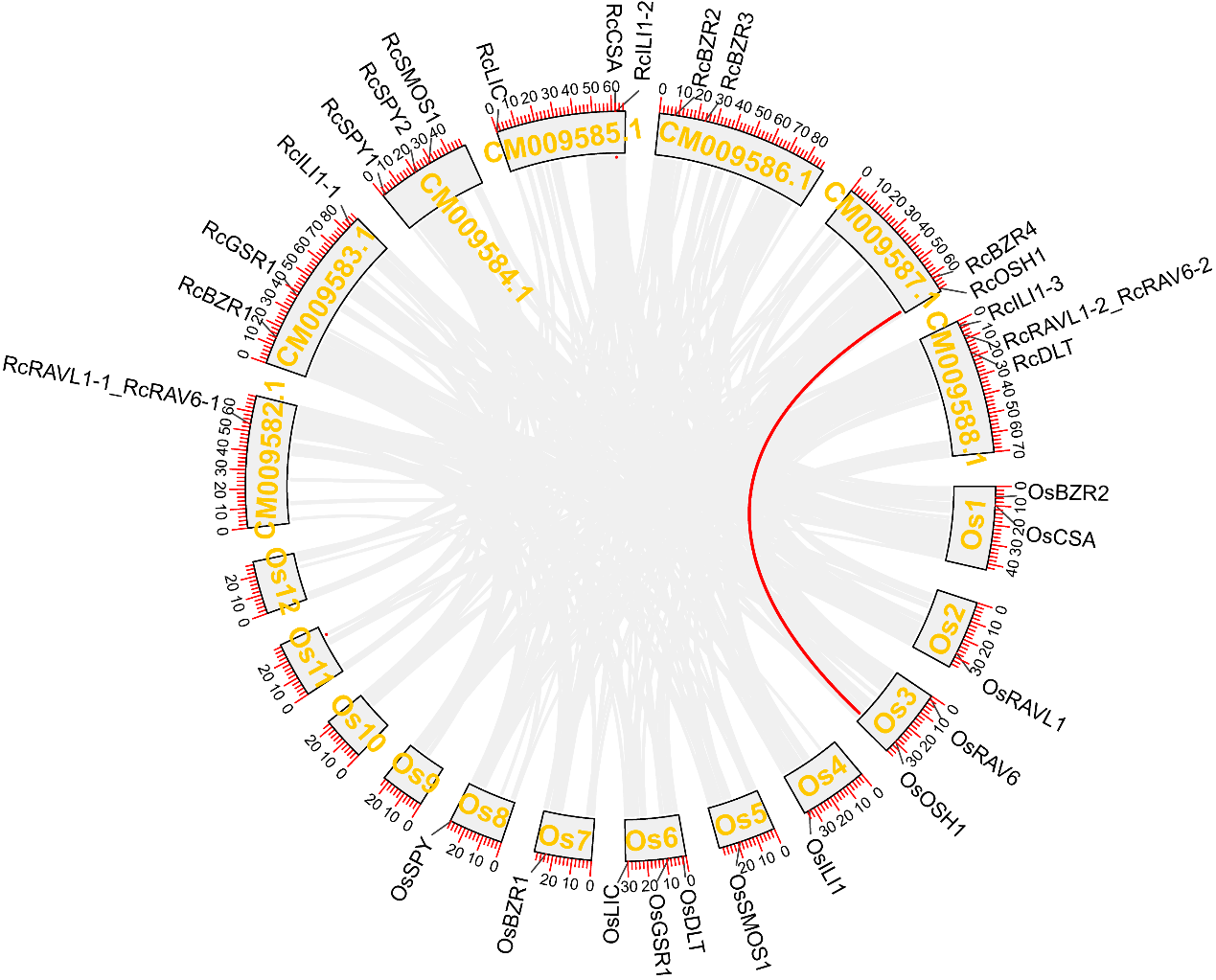


**Supplemental Figure 4-9. Analysis of the synteny among the BR downstream genes of *Oryza sativa* and *Prunus mume***


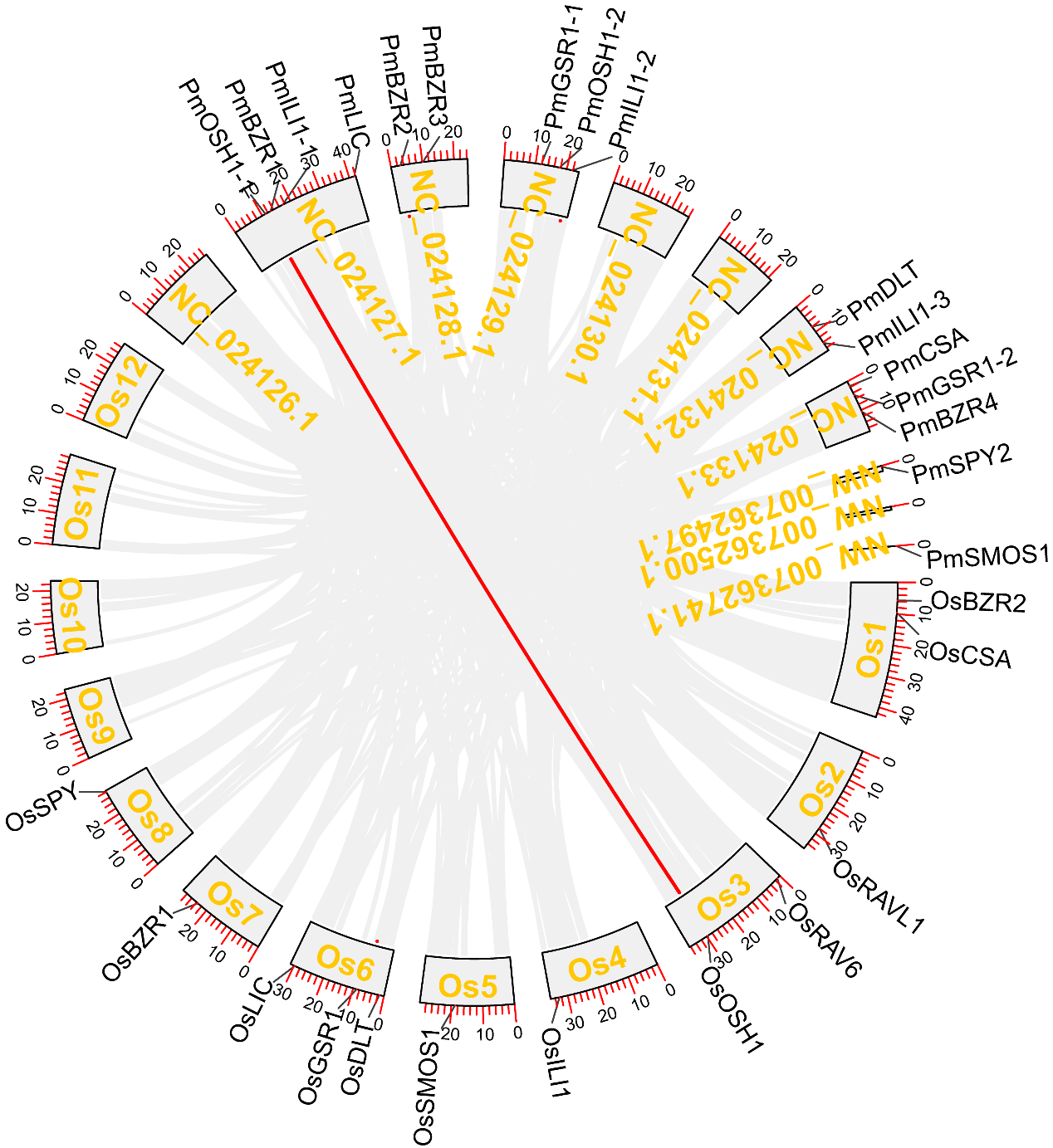

Supplement: Supplementary Figure 4 — Synteny analysis among BR downstream genes in Oryza sativa and each Rosaceae species. [file Data_Sheet_4.docx]
