# Supplementary material for: Genome-Wide Identification of Brassinosteroid Signaling Downstream Genes in Nine Rosaceae Species and Analyses of Their Roles in Stem Growth and Stress Response in Apple
Source: Front Genet. 2021 Mar 18;12:640271. doi: 10.3389/fgene.2021.640271 (PMC8012692; doi:10.3389/fgene.2021.640271)

**Supplemental Figure 5. The structure of BR downstream genes in nine rosaceae species**

**Supplemental Figure 5-1 The structure of BR downstream genes in *Malus domestica***


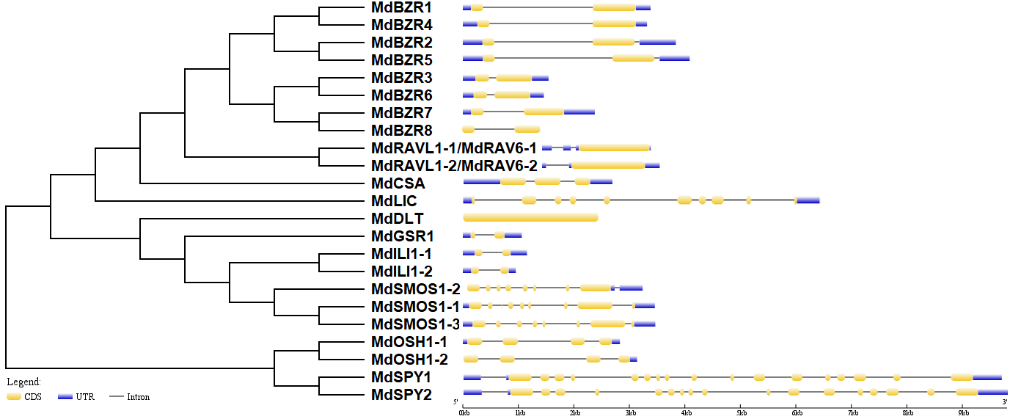


**Supplemental Figure 5-2 The structure of BR downstream genes in *Fragaria vesca***


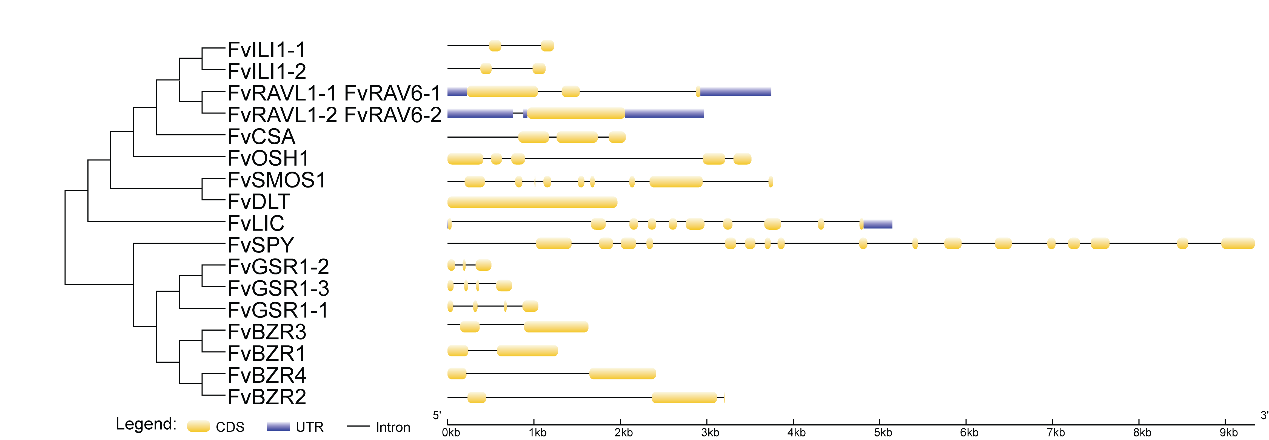


**Supplemental Figure 5-3 The structure of BR downstream genes in *Rubus occidentalis***


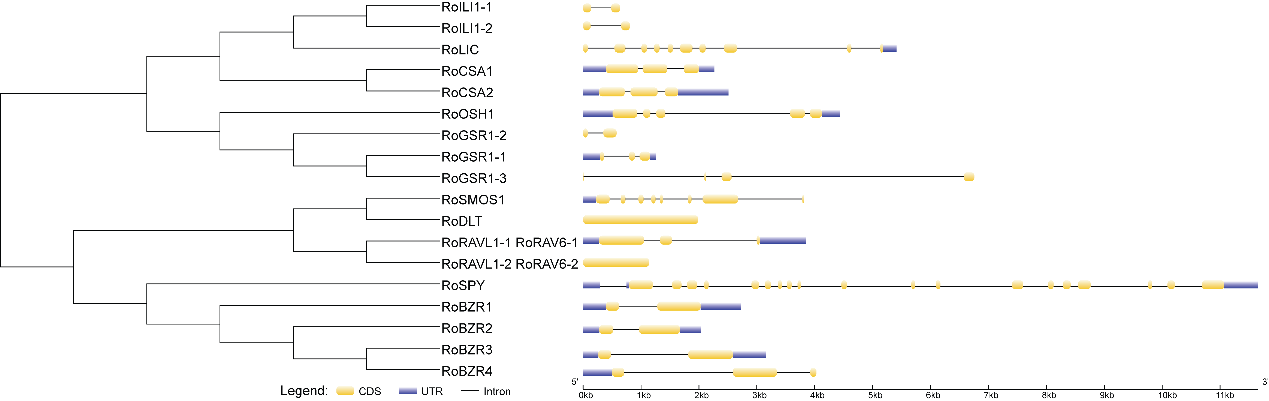


**Supplemental Figure 5-4 The structure of BR downstream genes in *Pyrus communis***
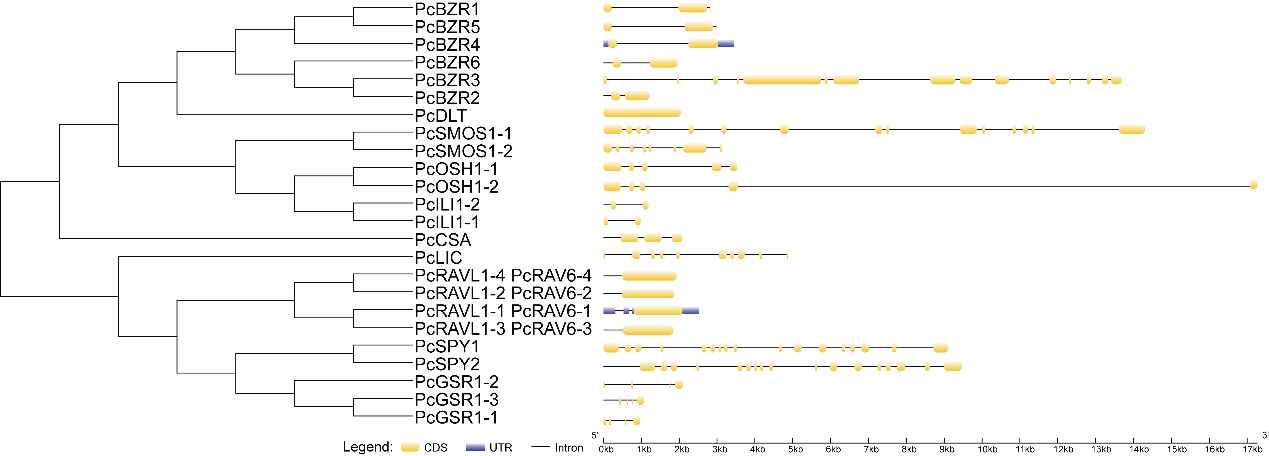


**Supplemental Figure 5-5 The structure of BR downstream genes in *Prunus persica***
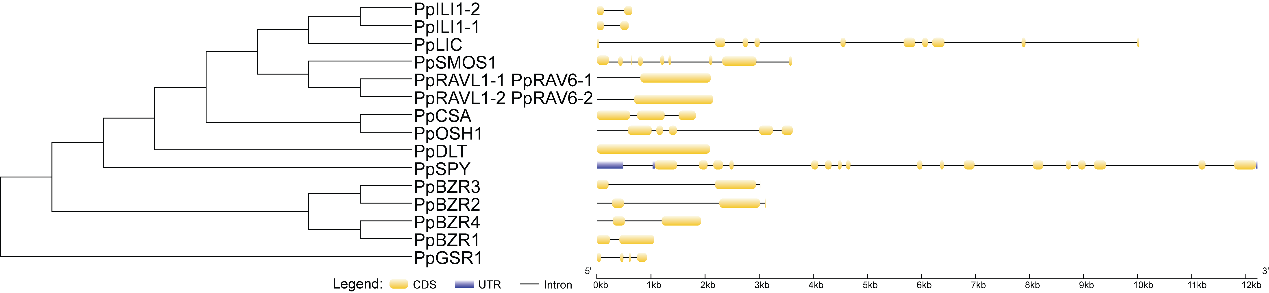


**Supplemental Figure 5-6 The structure of BR downstream genes in *Prunus avium***
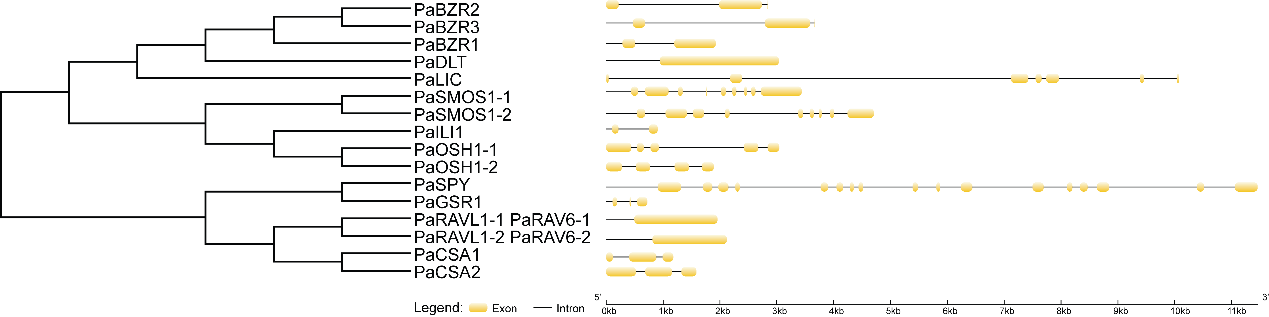


**Supplemental Figure 5-7 The structure of BR downstream genes in *Prunus dulcis***
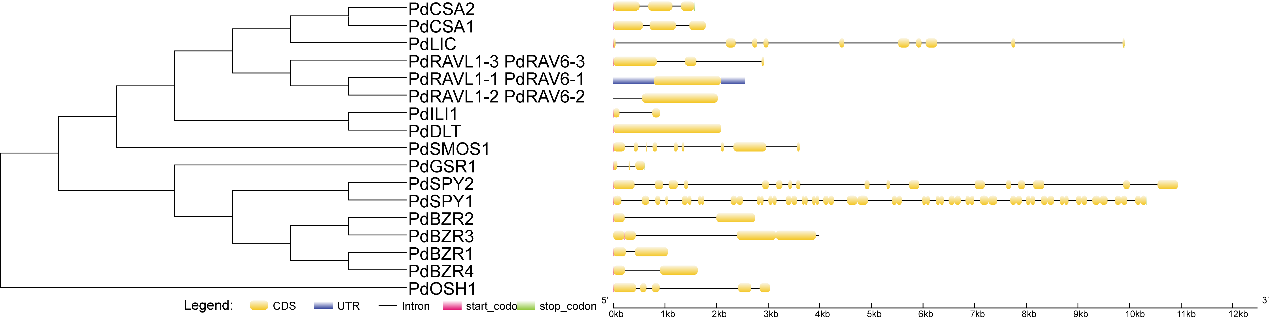


**Supplemental Figure 5-8 The structure of BR downstream genes in *Rosa chinensis***
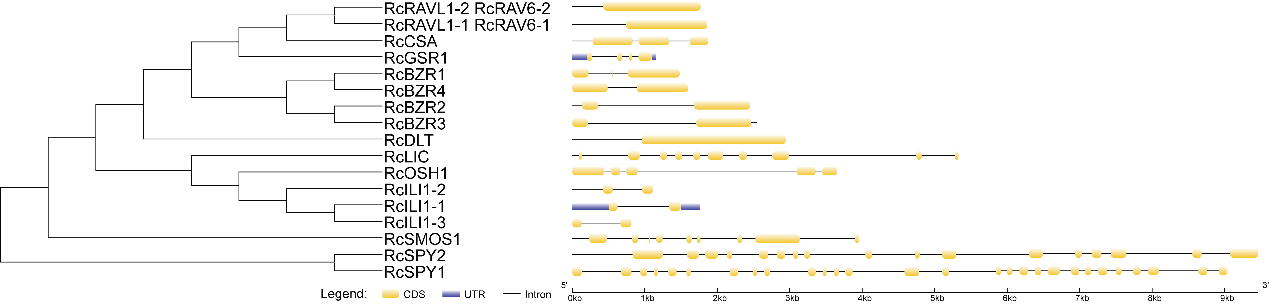


**Supplemental Figure 5-9 The structure of BR downstream genes in *Prunus mume***
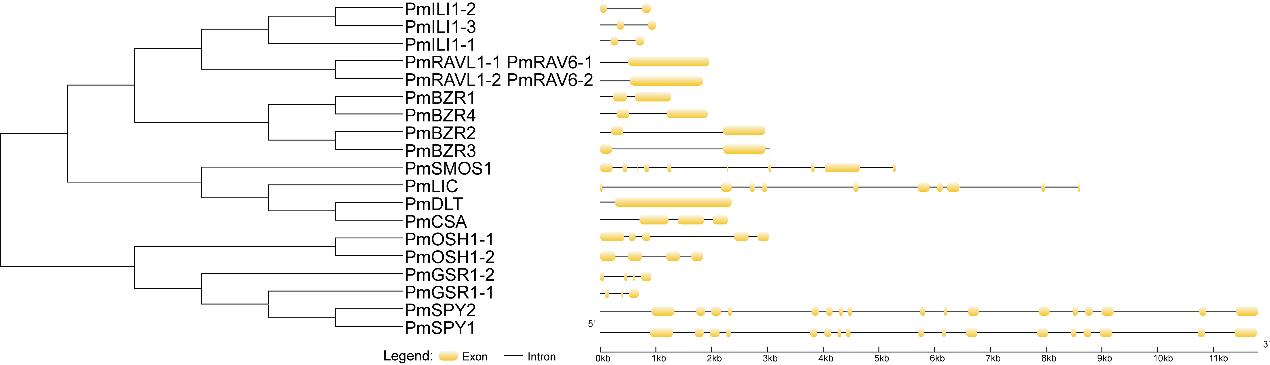

Supplement: Supplementary Figure 5 — Structure of BR downstream genes in nine Rosaceae species. [file Data_Sheet_5.docx]
