# Supplementary material for: Genome-Wide Identification of Brassinosteroid Signaling Downstream Genes in Nine Rosaceae Species and Analyses of Their Roles in Stem Growth and Stress Response in Apple
Source: Front Genet. 2021 Mar 18;12:640271. doi: 10.3389/fgene.2021.640271 (PMC8012692; doi:10.3389/fgene.2021.640271)

**Supplemental Figure 6 Alignment of multiple BR downstream protein in *Arabidopsis*, rice, nine rosaceae species**

**Supplemental Figure 6-1 Alignment of multiple BZR proteins.**

Conserved domains of BZRs are indicated with colored rectangles (purple, NLS; black, bHLH; light green, serine rich phosphorylation sites; blue, PEST sequences; and pink, C-terminal domain). Conserved amino acid residues of Basic, Helix1, Loop and Helix2 motifs are indicated with red lines.


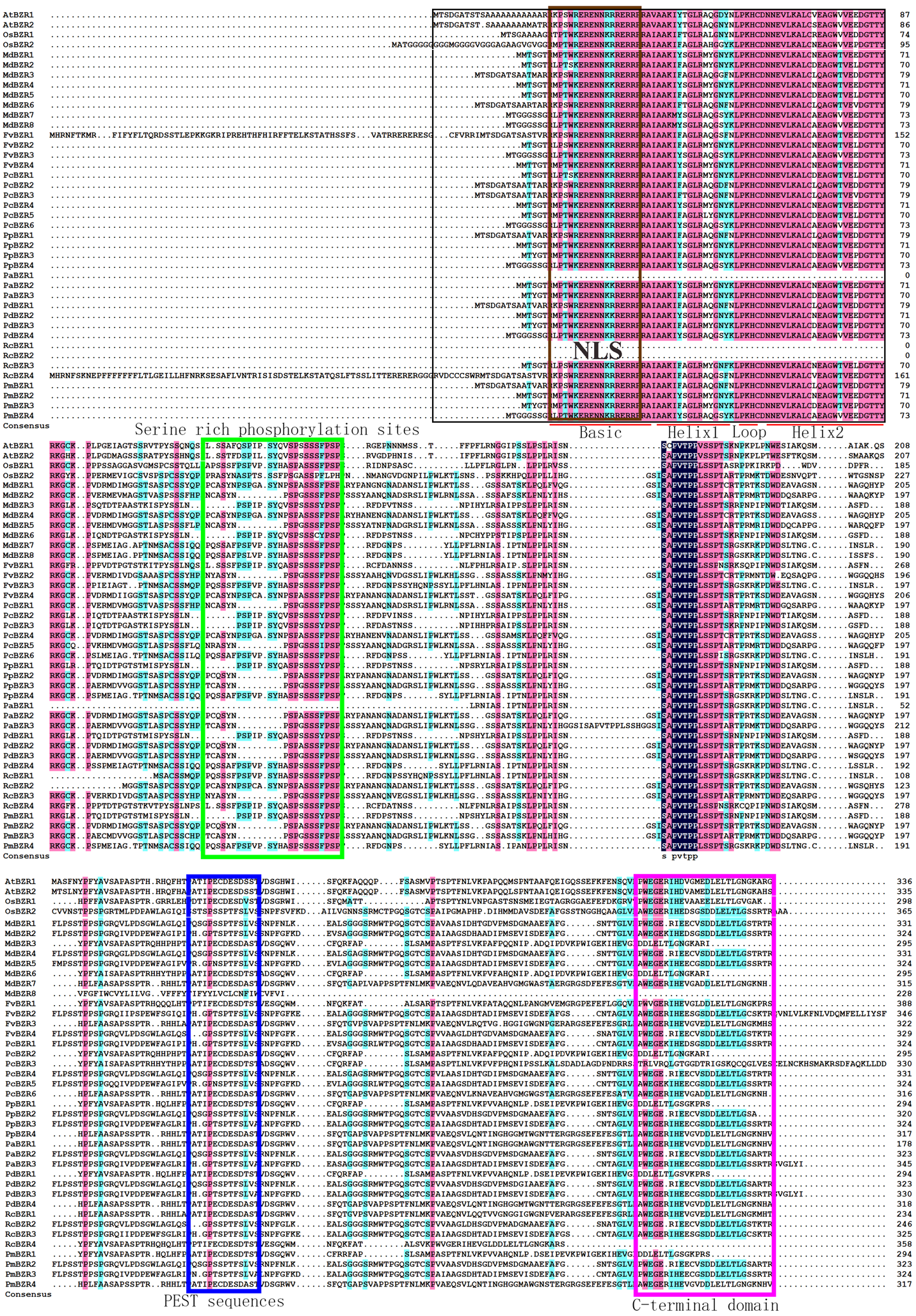


**Supplemental Figure 6-2 Alignment of multiple DLT proteins.**

Black, blue, red, pink, and indigo underlines respectively represent leucine heptad I domain with Ⅰ A and Ⅰ B regions, VHIID domain, leucine heptad II domain containing II A and II B regions, PFYRE domain and SAW domain, respectively.


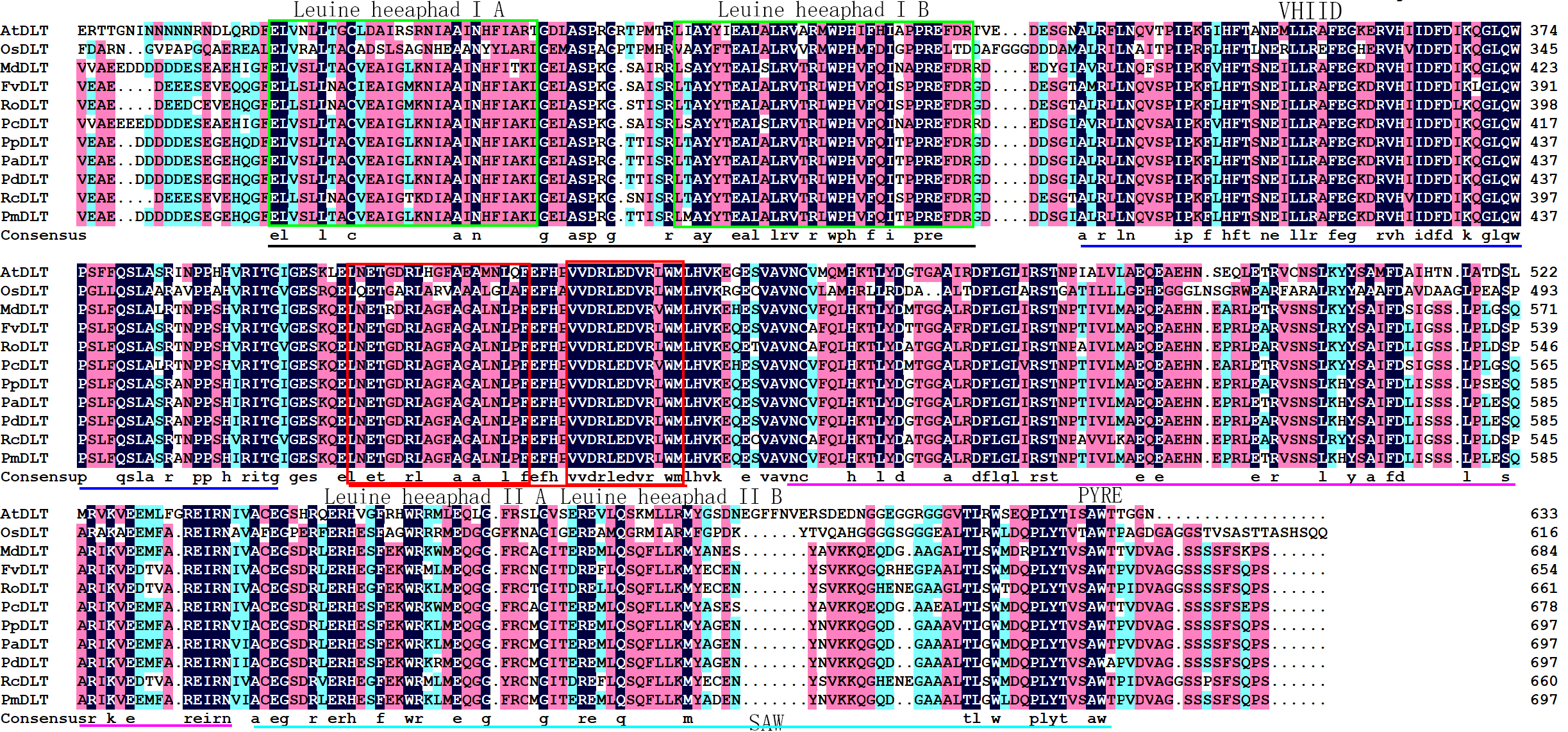


**Supplemental Figure 6-3 Alignment of multiple RAVL1/RAV6 proteins.**

B3 DNA-binding domain is indicated by black underline in RAVL1-RAV6 proteins


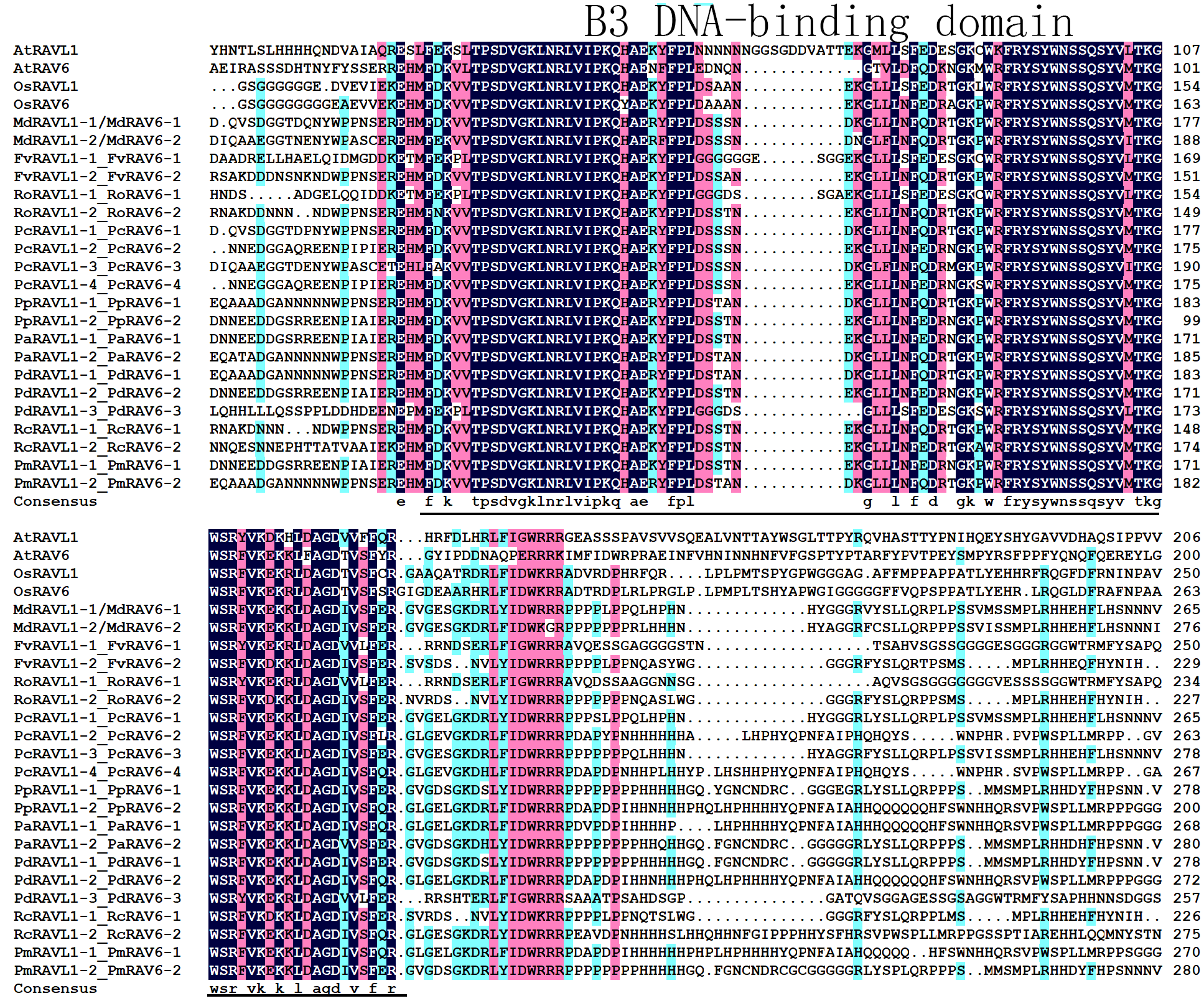


**Supplemental Figure 6-4 Alignment of multiple LIC proteins.**

CCCH domain, EELR domain and serine rich region are indicated by red, bottle-green and indigo rectangles, respectively


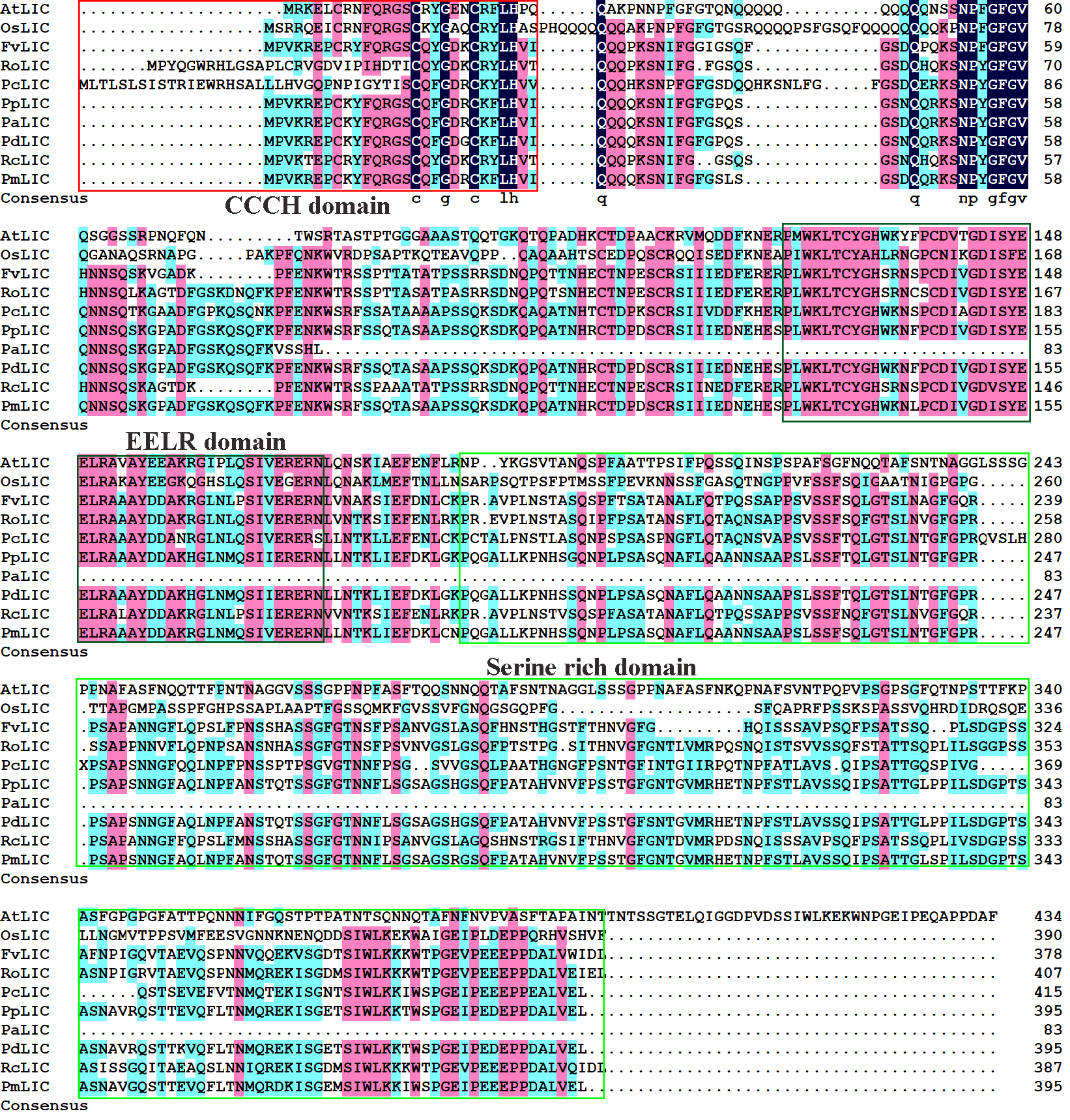


**Supplemental Figure 6-5 Alignment of multiple OSH1 proteins.**

Black and light green underlines respectively indicate KNOX domain and ELK domain; In ELK domain, red, black, red and blue lines respectively represent basic, helix I, helix II, and helix Ⅲ.


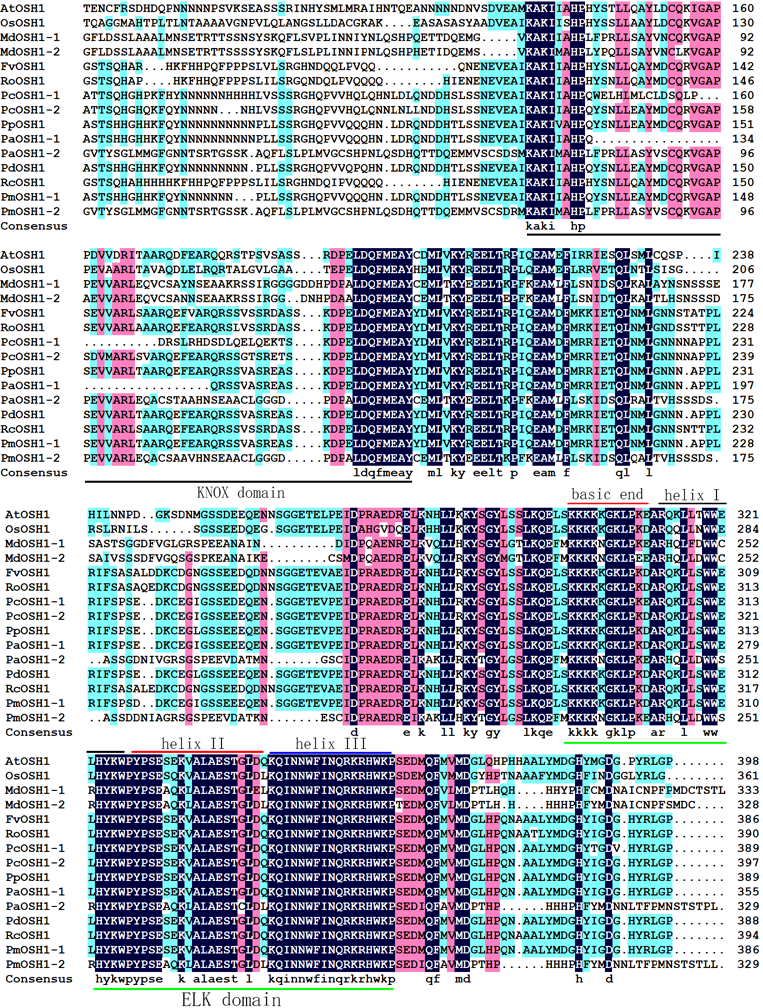


**Supplemental Figure 6-6 Alignment of multiple SMOS1 proteins.**

Black, red, indigo, blue, and azury underlines respectively represent AKER domain, AP2 domain (containing AP2-R1 domain, black rectangle; linker region, indigo rectangle and AP2-R2 domain, azury rectangle), EPY domain, ILS domain, and WTNF domain


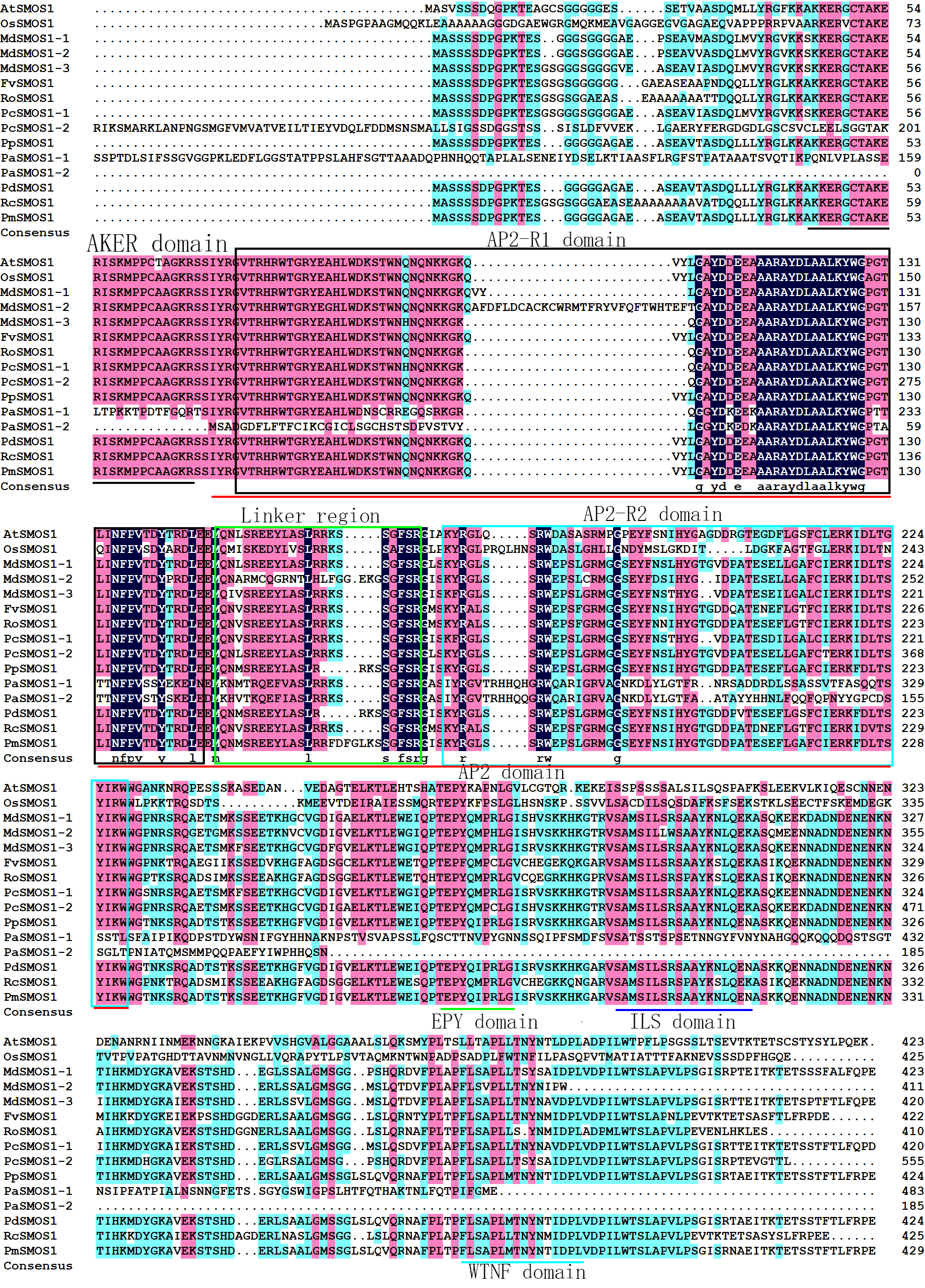


**Supplemental Figure 6-7 Alignment of multiple CSA proteins.**

Red and light green rectangles represent R2 domain and R3 domain, respectively


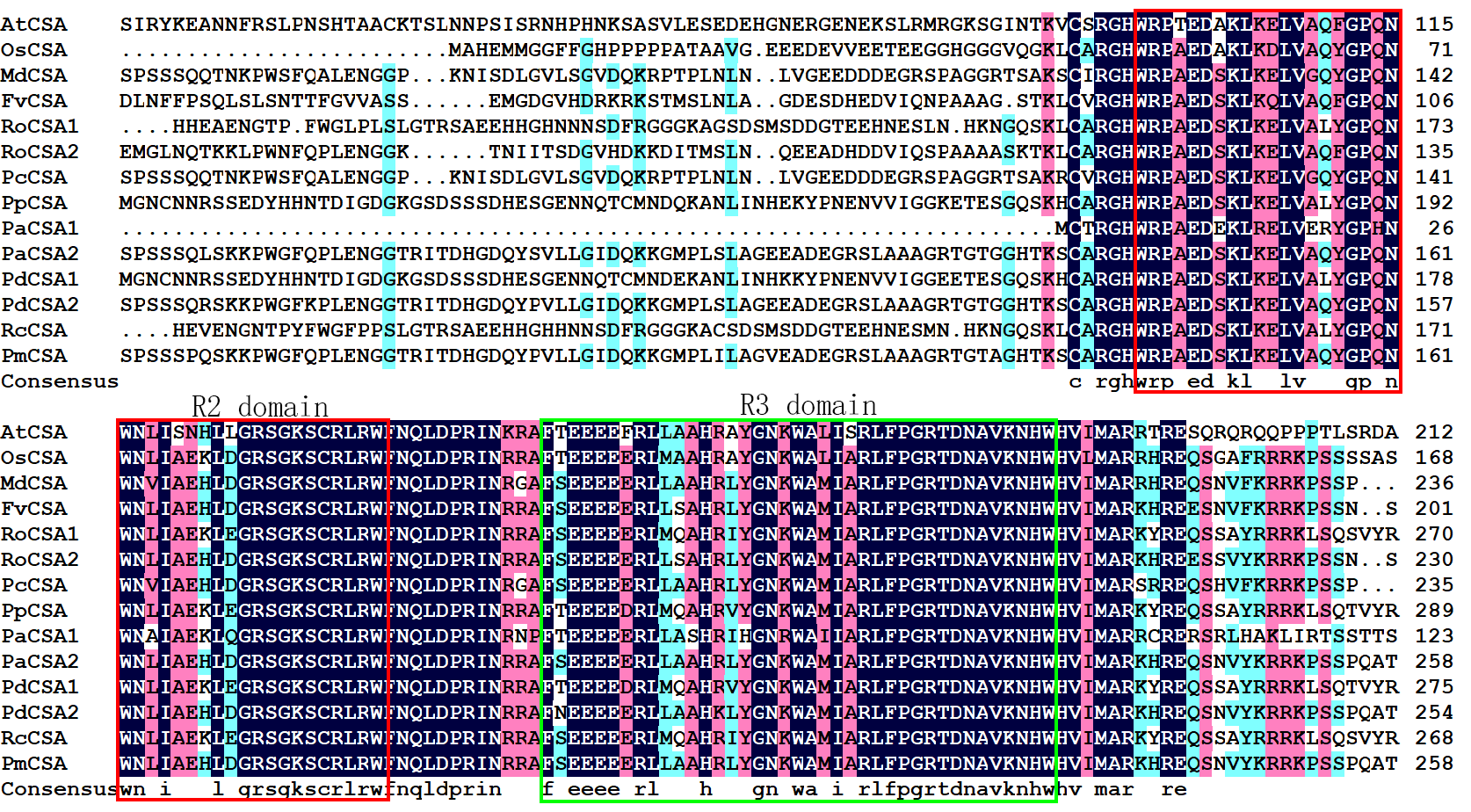


**Supplemental Figure 6-8 Alignment of multiple GSR1 proteins.**

Black underline, represent C-terminal region, conserved cys sites are indicated by asterisks


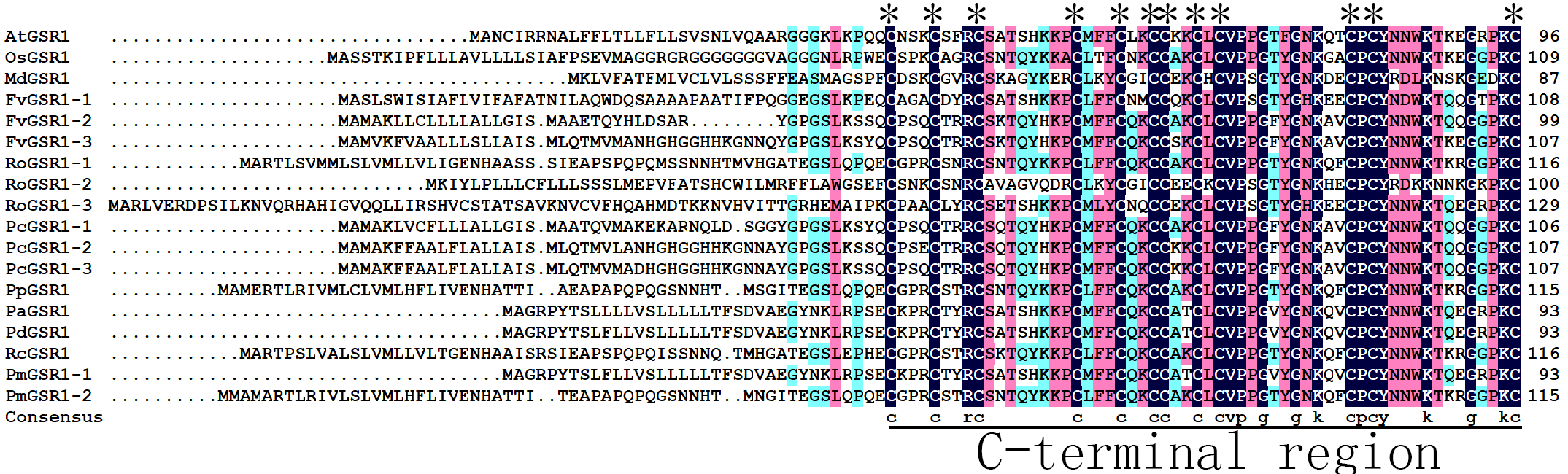


**Supplemental Figure 6- 9 Alignment of multiple SPY proteins.**

Conserved TPR domain, CD I and CD II are respectively indicated by red, green, and black underlines


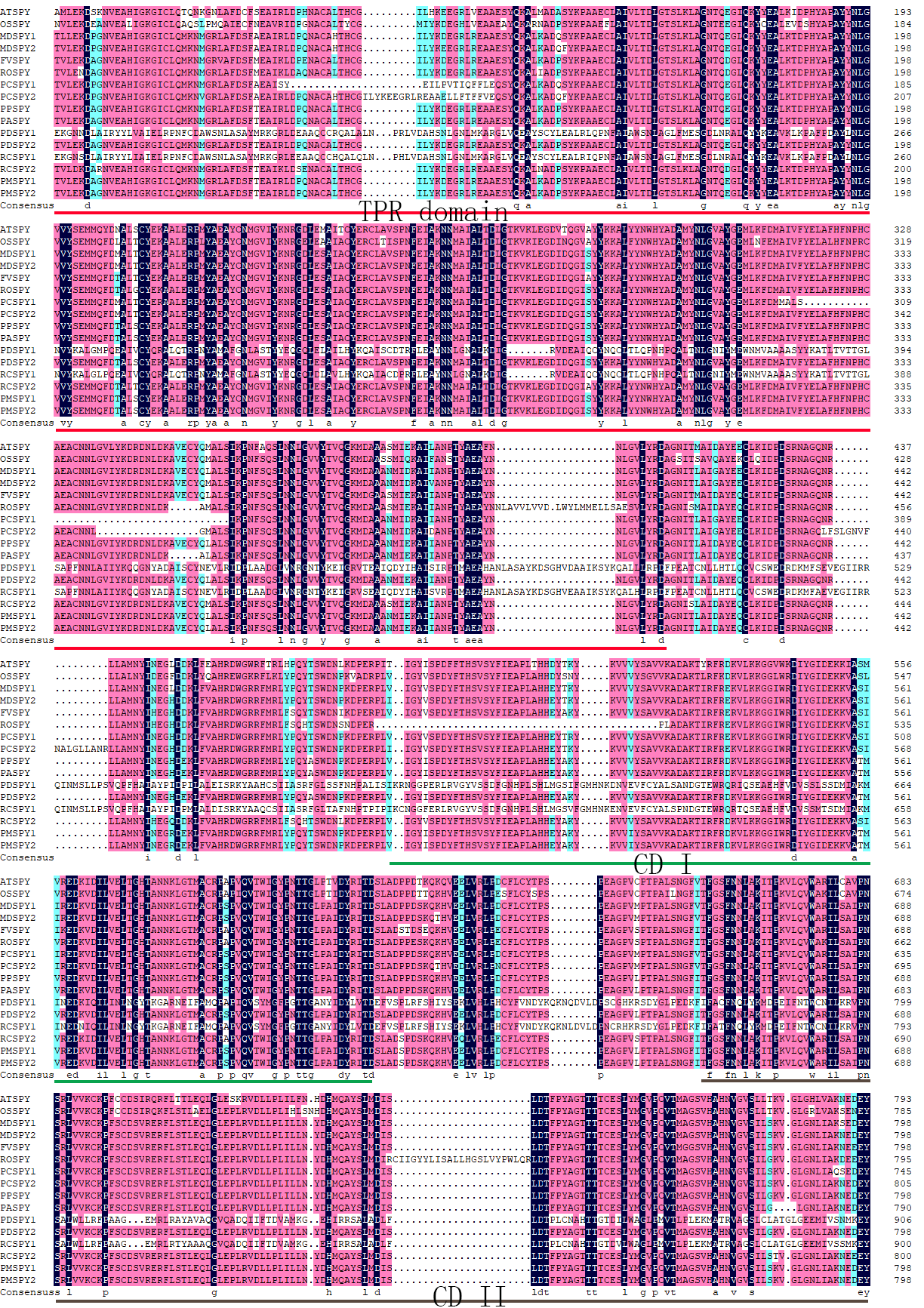


**Supplemental Figure 6- 10 Alignment of multiple ILI1 proteins.**

Two helix and loop regions were indicated by black underlines.


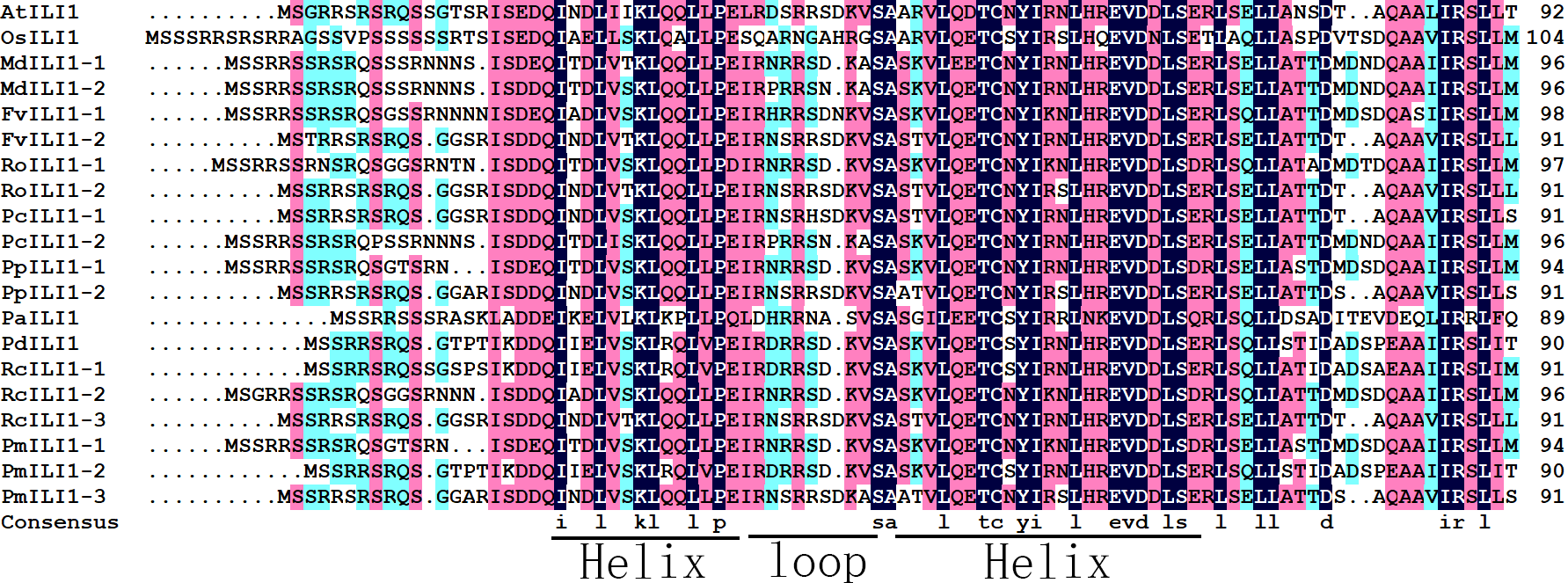

Supplement: Supplementary Figure 6 — Alignment of multiple BR downstream proteins in Arabidopsis, rice, and nine Rosaceae species. [file Data_Sheet_6.docx]
