# Supplementary material for: Genome-Wide Identification of Brassinosteroid Signaling Downstream Genes in Nine Rosaceae Species and Analyses of Their Roles in Stem Growth and Stress Response in Apple
Source: Front Genet. 2021 Mar 18;12:640271. doi: 10.3389/fgene.2021.640271 (PMC8012692; doi:10.3389/fgene.2021.640271)

**Supplemental Figure 7 Predicted secondary structures of BR downstream proteins**

**Supplemental Figure 7-1 Predicted secondary structures of BZR proteins**


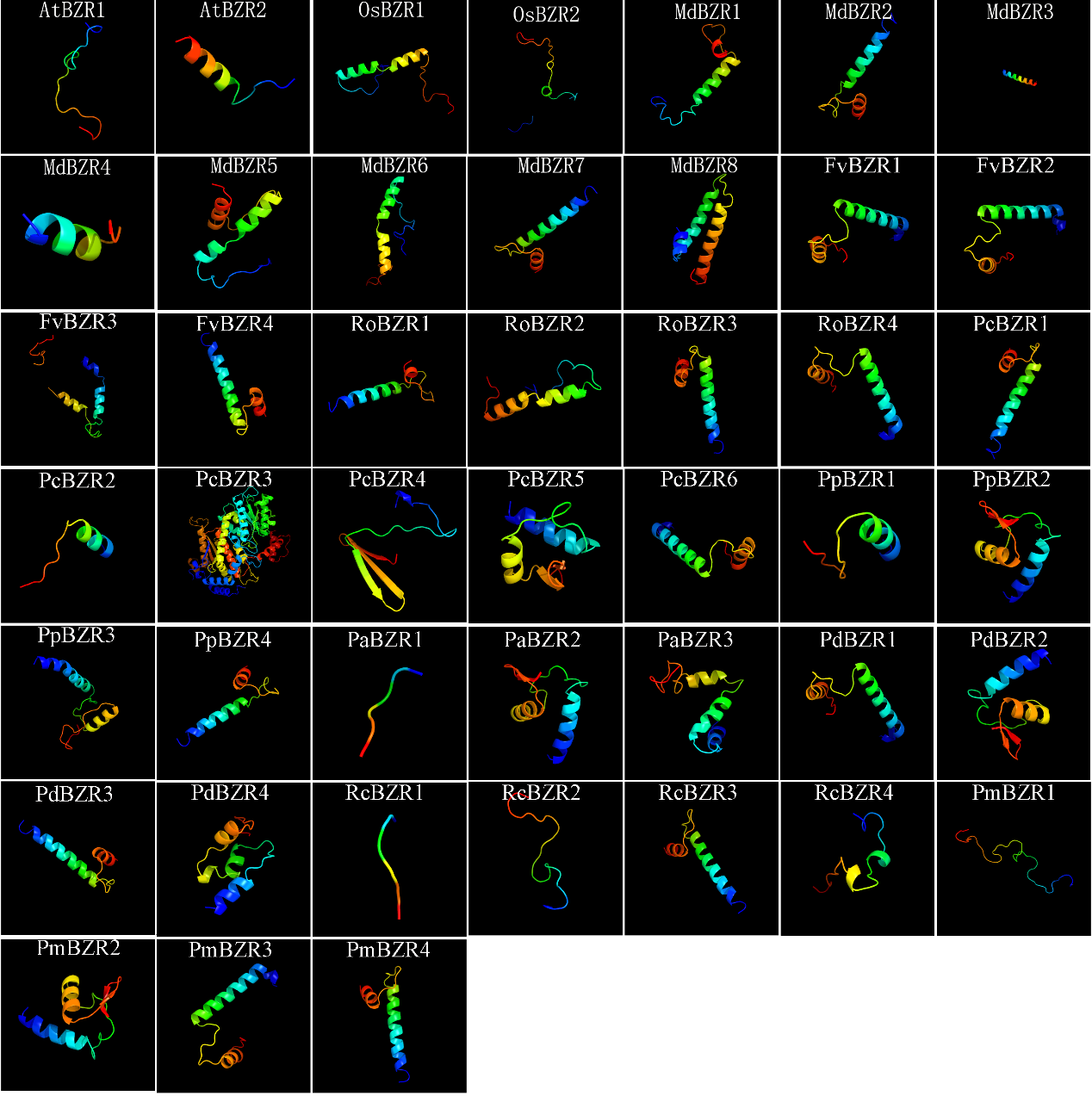


**Supplemental Figure 7-2 Predicted secondary structures of DLT proteins**


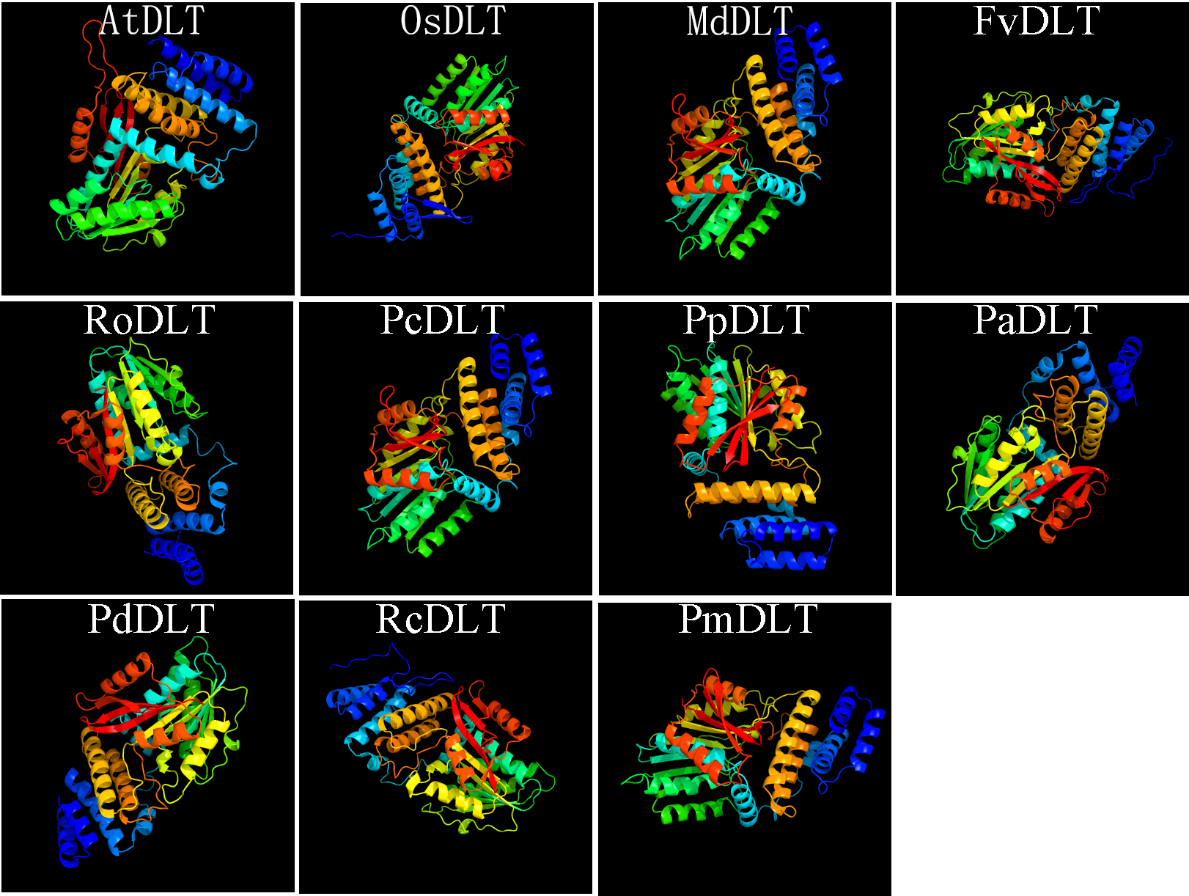


**Supplemental Figure 7-3 Predicted secondary structures of LIC proteins**


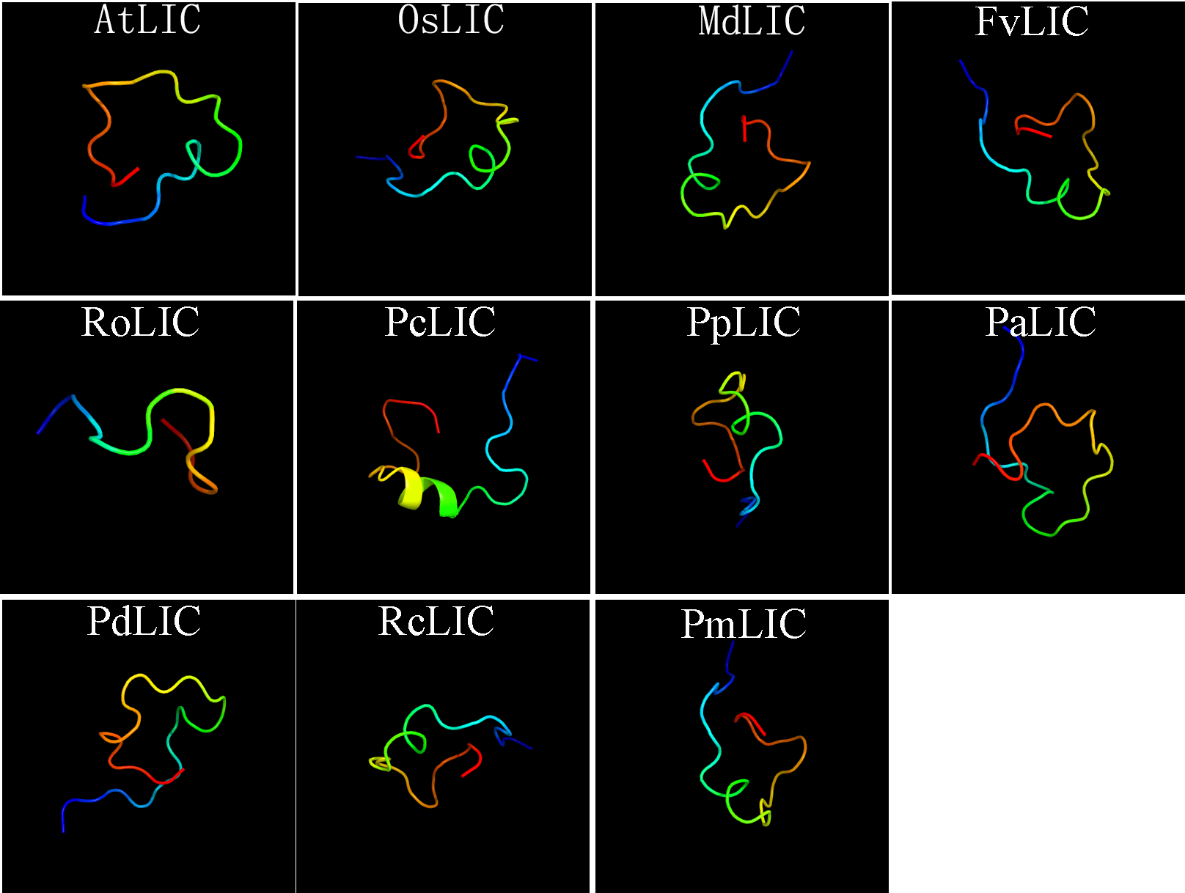


**Supplemental Figure 7-4 Predicted secondary structures of ILI1 proteins**


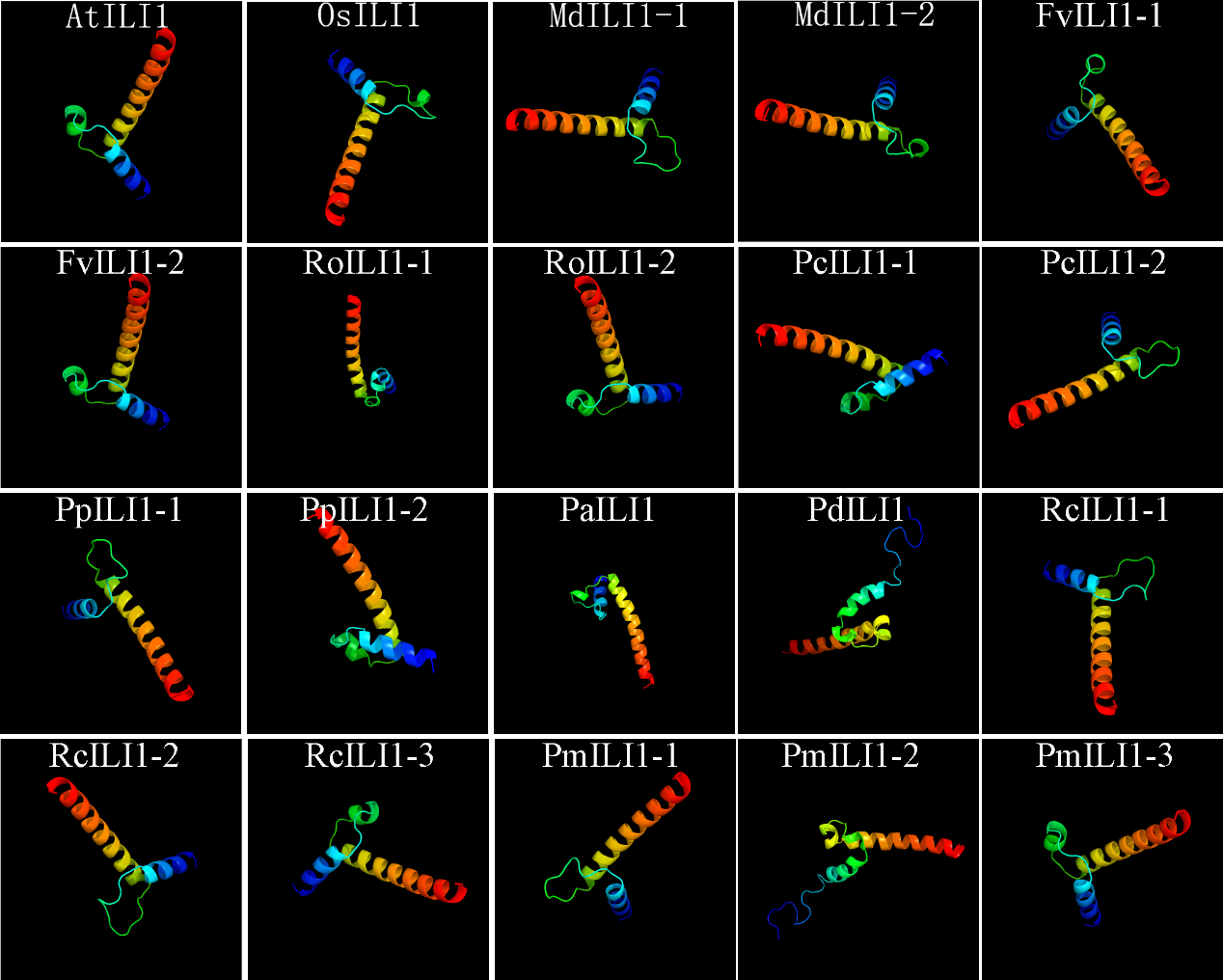


**Supplemental Figure 7-5 Predicted secondary structures of OSH1 proteins**


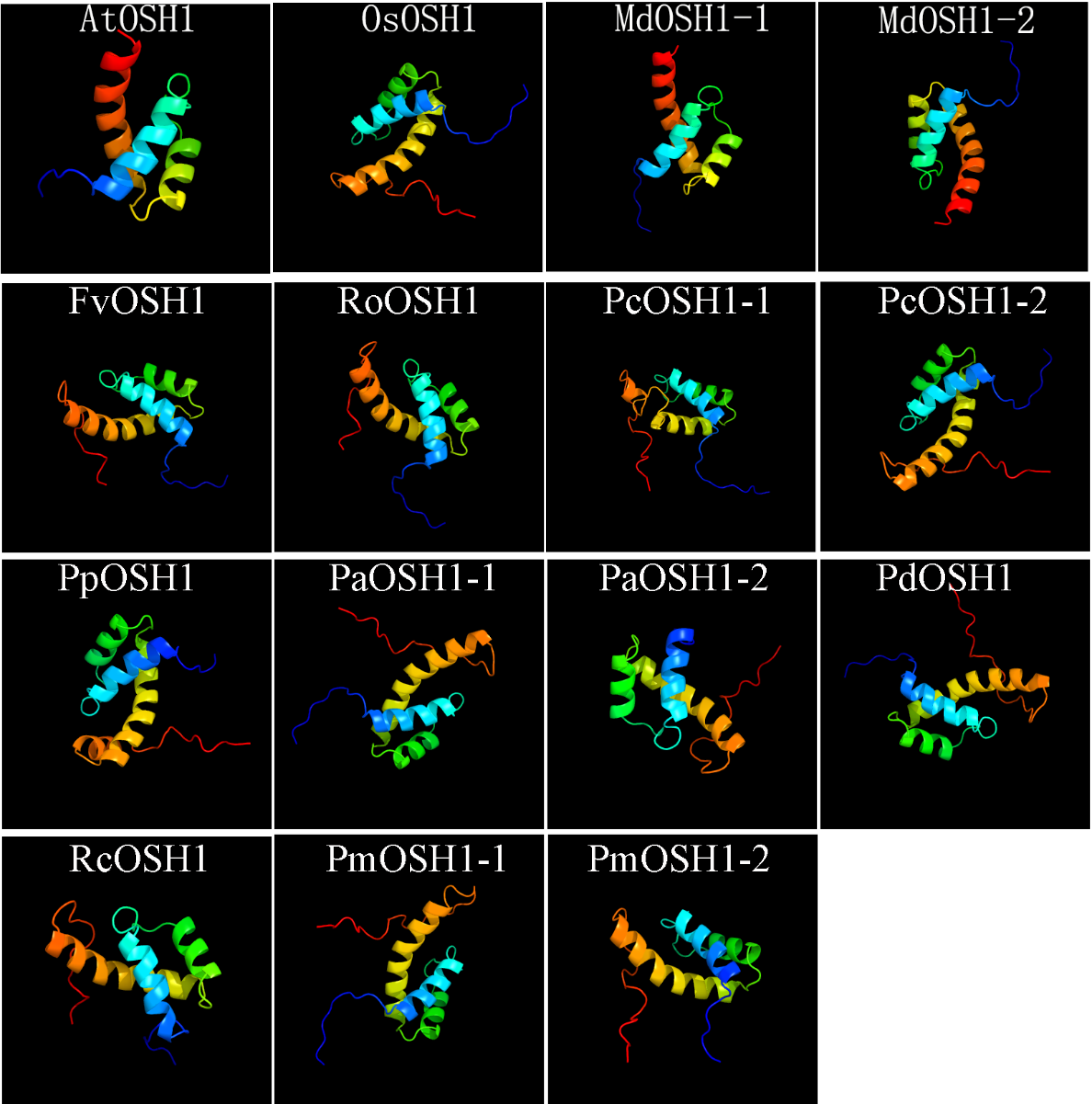


**Supplemental Figure 7-6 Predicted secondary structures of RAVL1/RAV6 proteins**


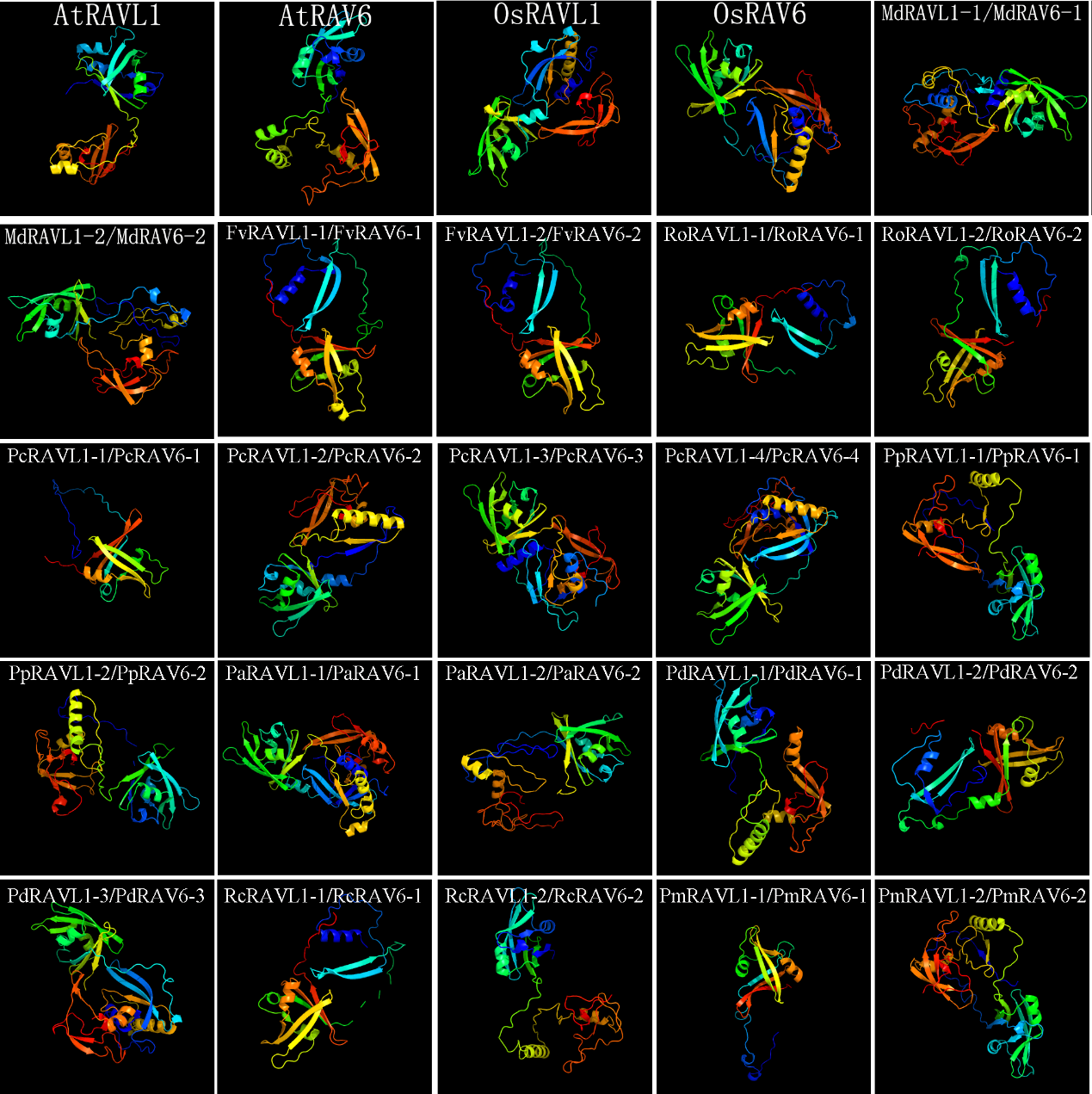


**Supplemental Figure 7-7 Predicted secondary structures of SMOS1 proteins**


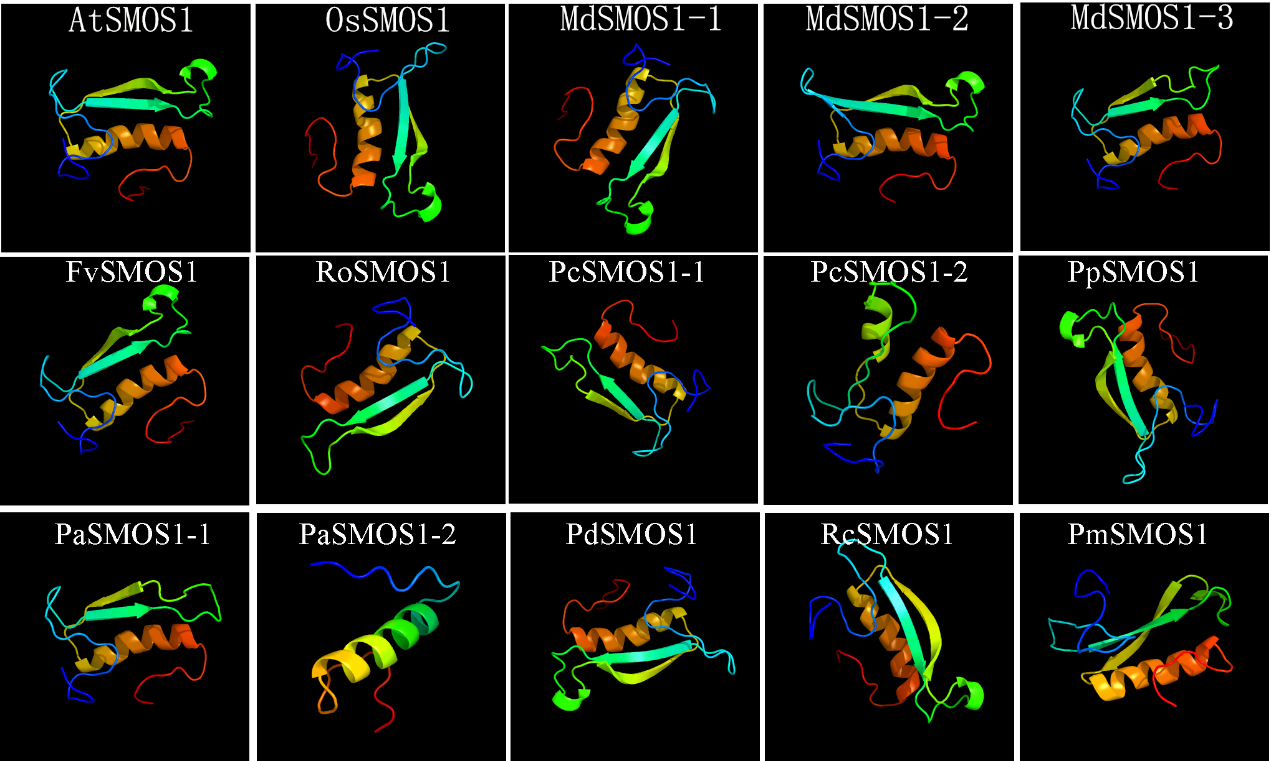


**Supplemental Figure 7-8 Predicted secondary structures of CSA proteins**


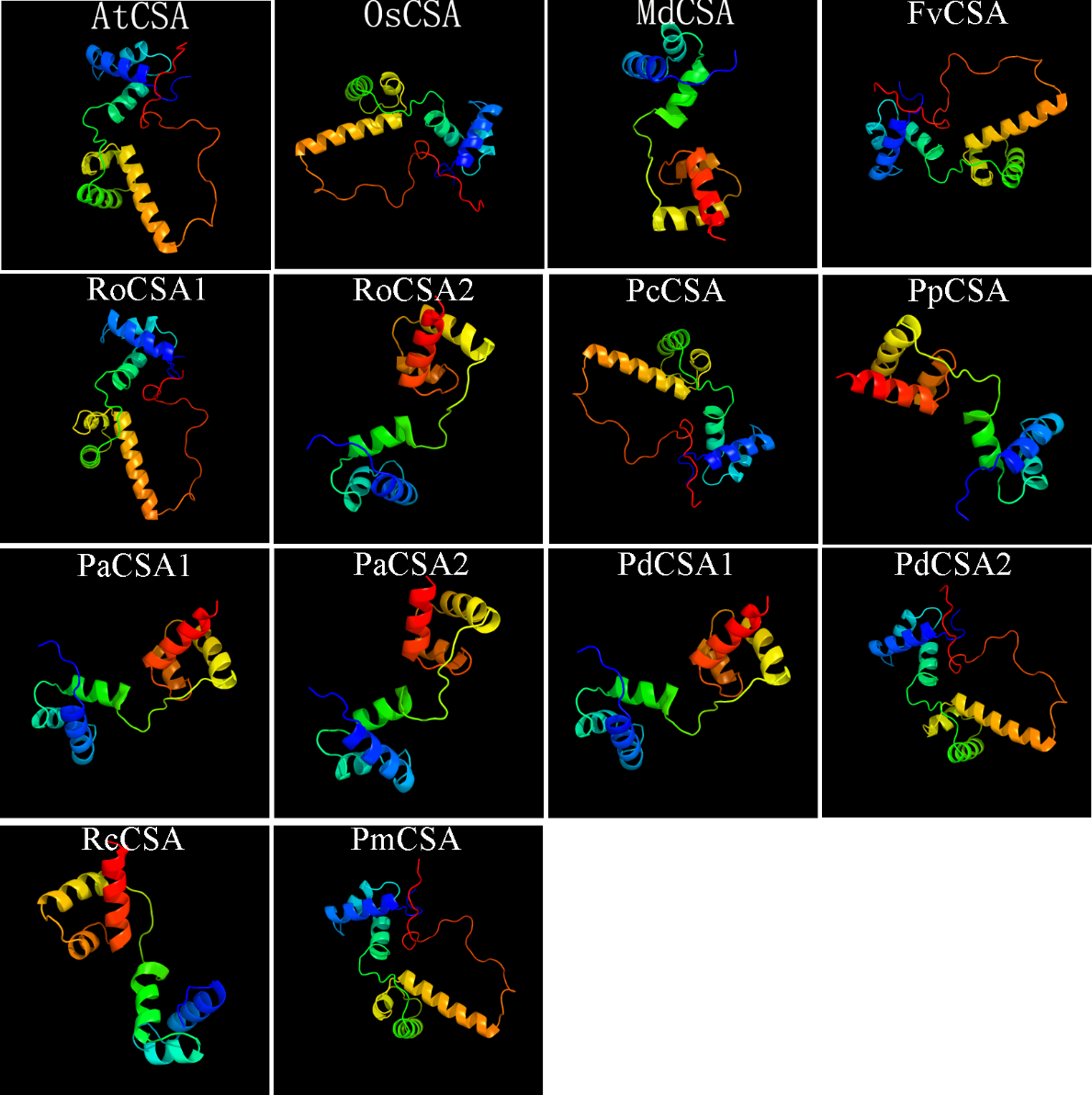


**Supplemental Figure 7-9 Predicted secondary structures of SPY proteins**


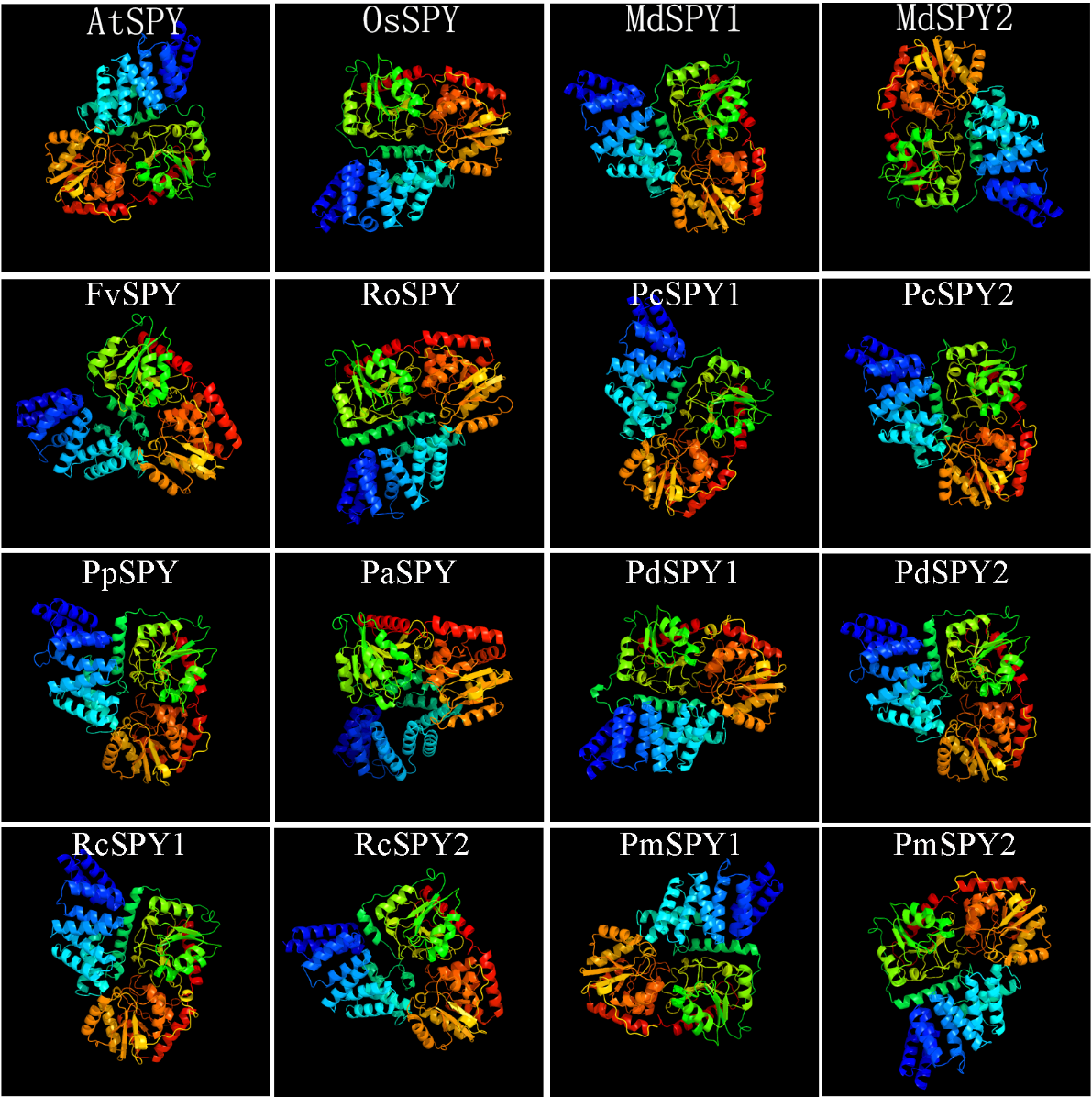


**Supplemental Figure 7-10 Predicted secondary structures of GSR1 proteins**


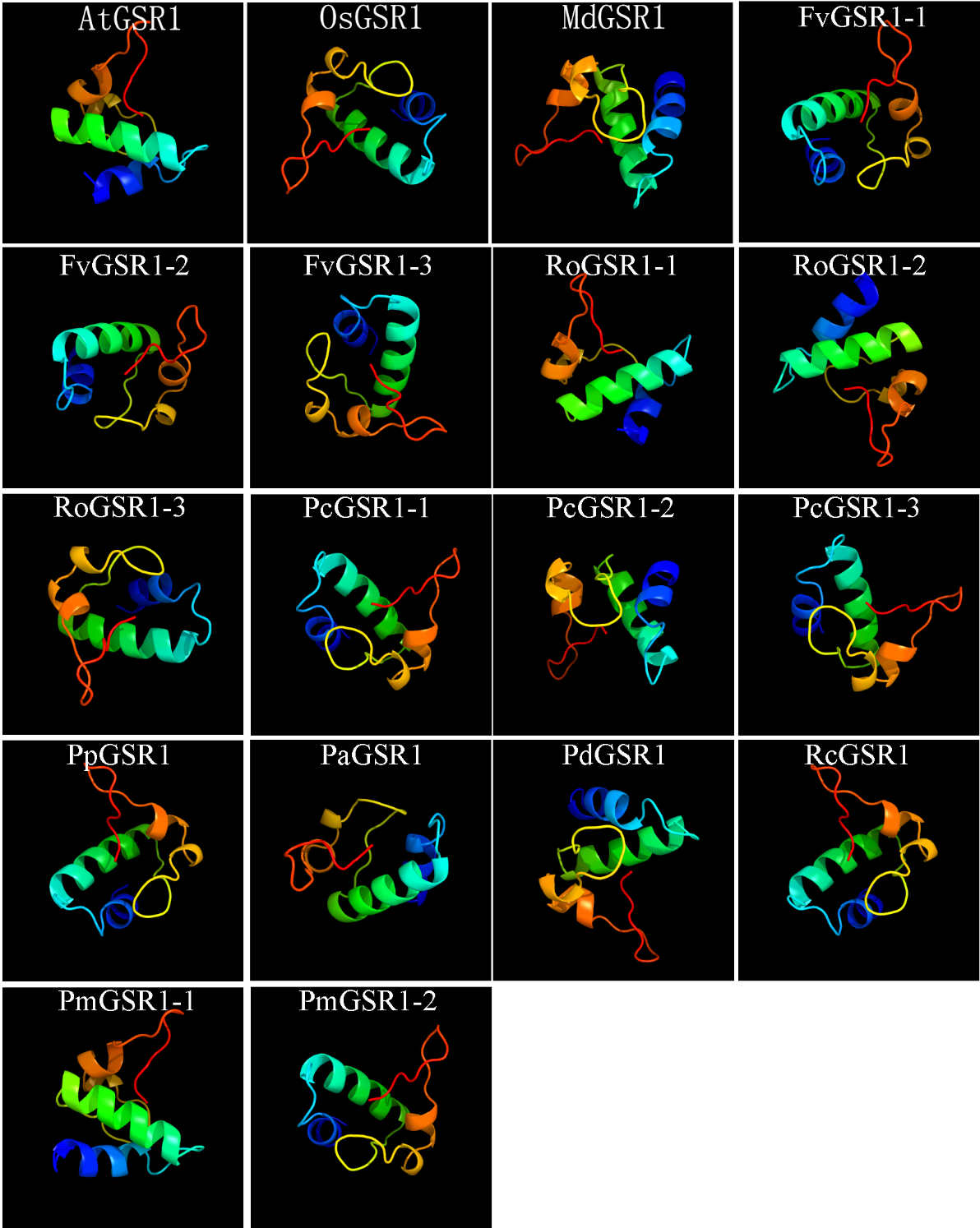

Supplement: Supplementary Figure 7 — Predicted secondary structures of BR downstream proteins. [file Data_Sheet_7.docx]
