# Supplementary figures and images for: Genome-Wide Identification of Brassinosteroid Signaling Downstream Genes in Nine Rosaceae Species and Analyses of Their Roles in Stem Growth and Stress Response in Apple
Source: Front Genet. 2021 Mar 18;12:640271. doi: 10.3389/fgene.2021.640271 (PMC8012692; doi:10.3389/fgene.2021.640271)

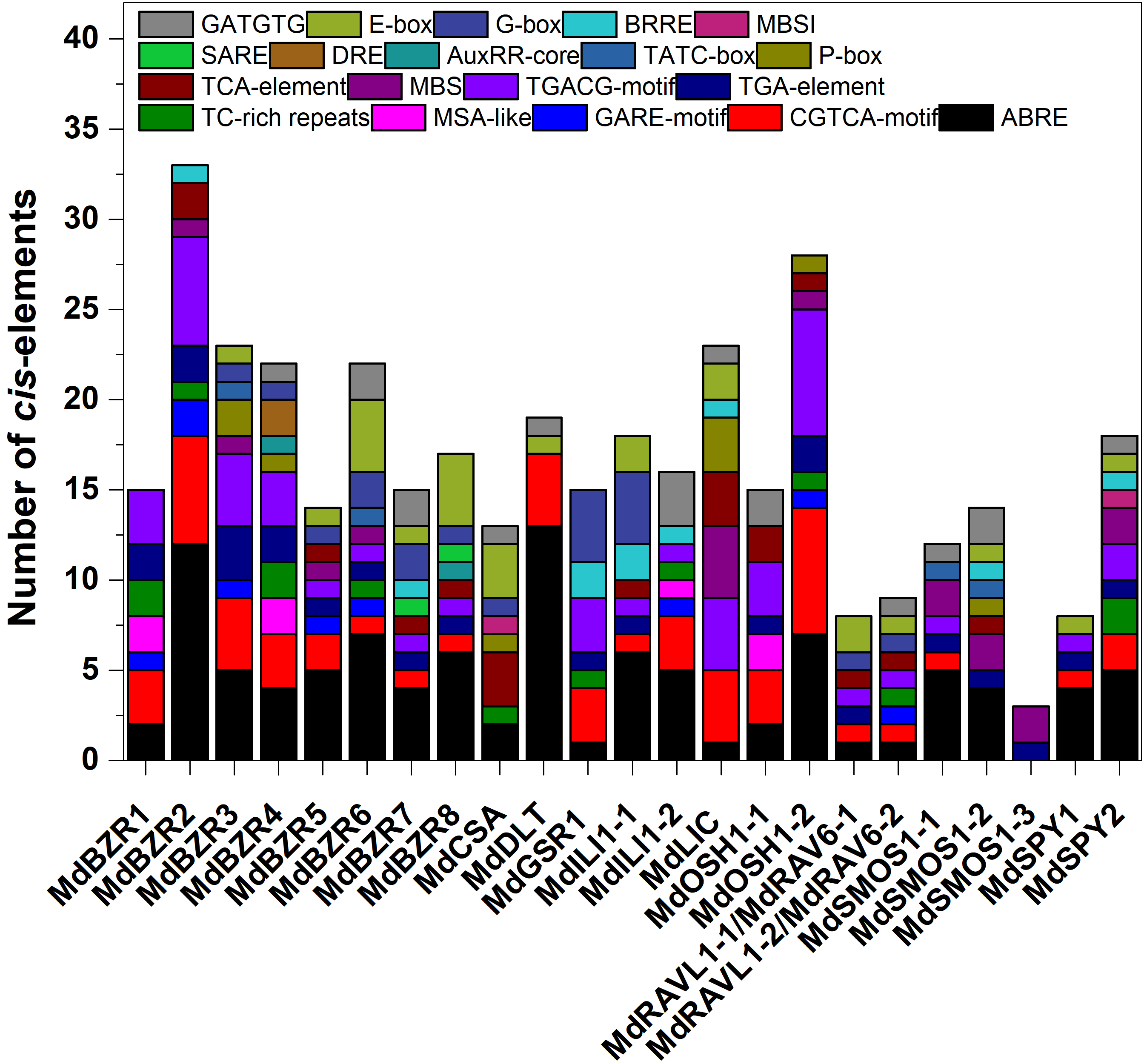

Supplement: Supplementary Figure 8 — Promoter analysis of apple BR downstream genes. [file Image_1.PNG]

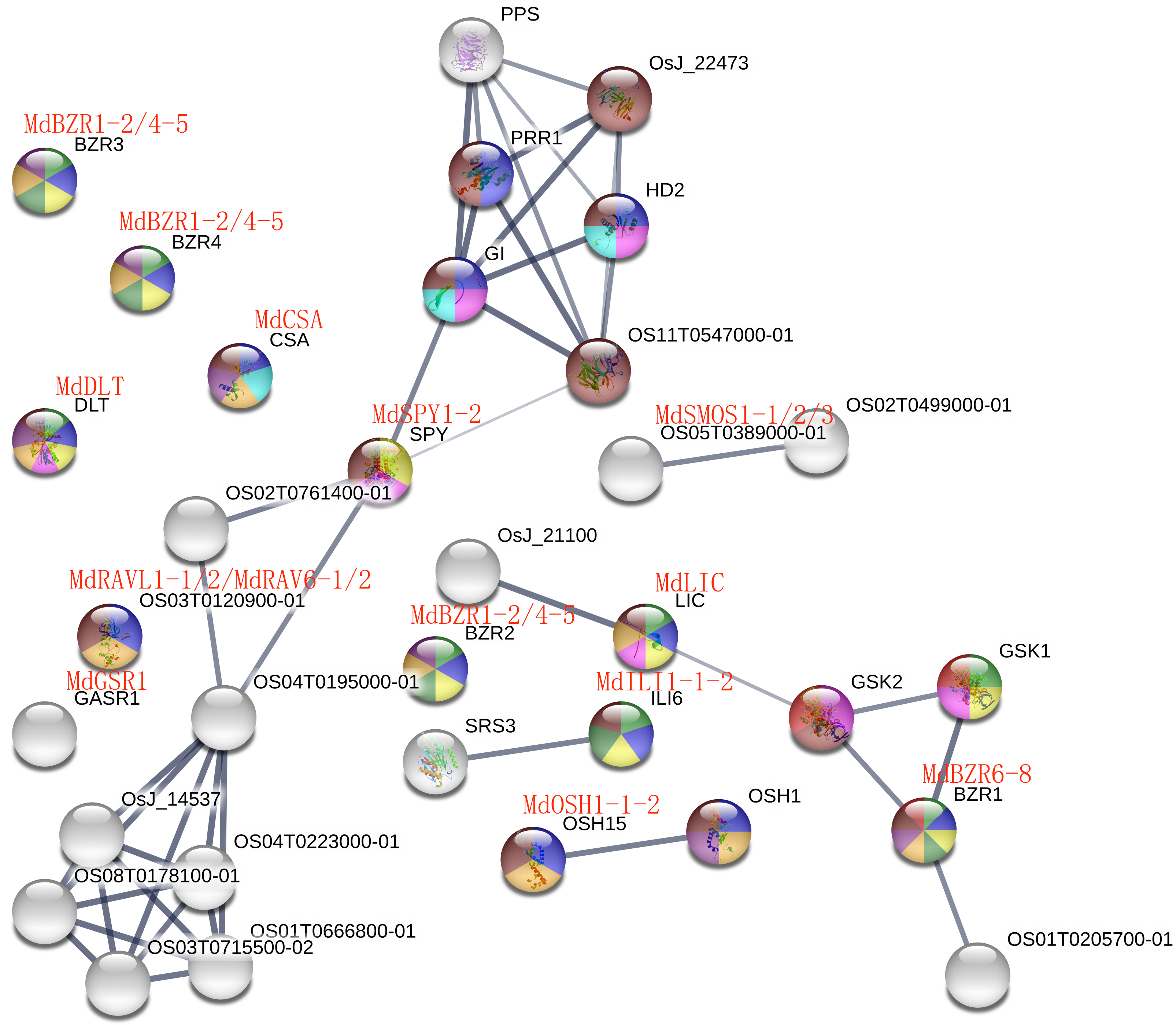

Supplement: Supplementary Figure 9 — Protein-protein interaction analysis of apple BR downstream proteins. [file Image_2.PNG]

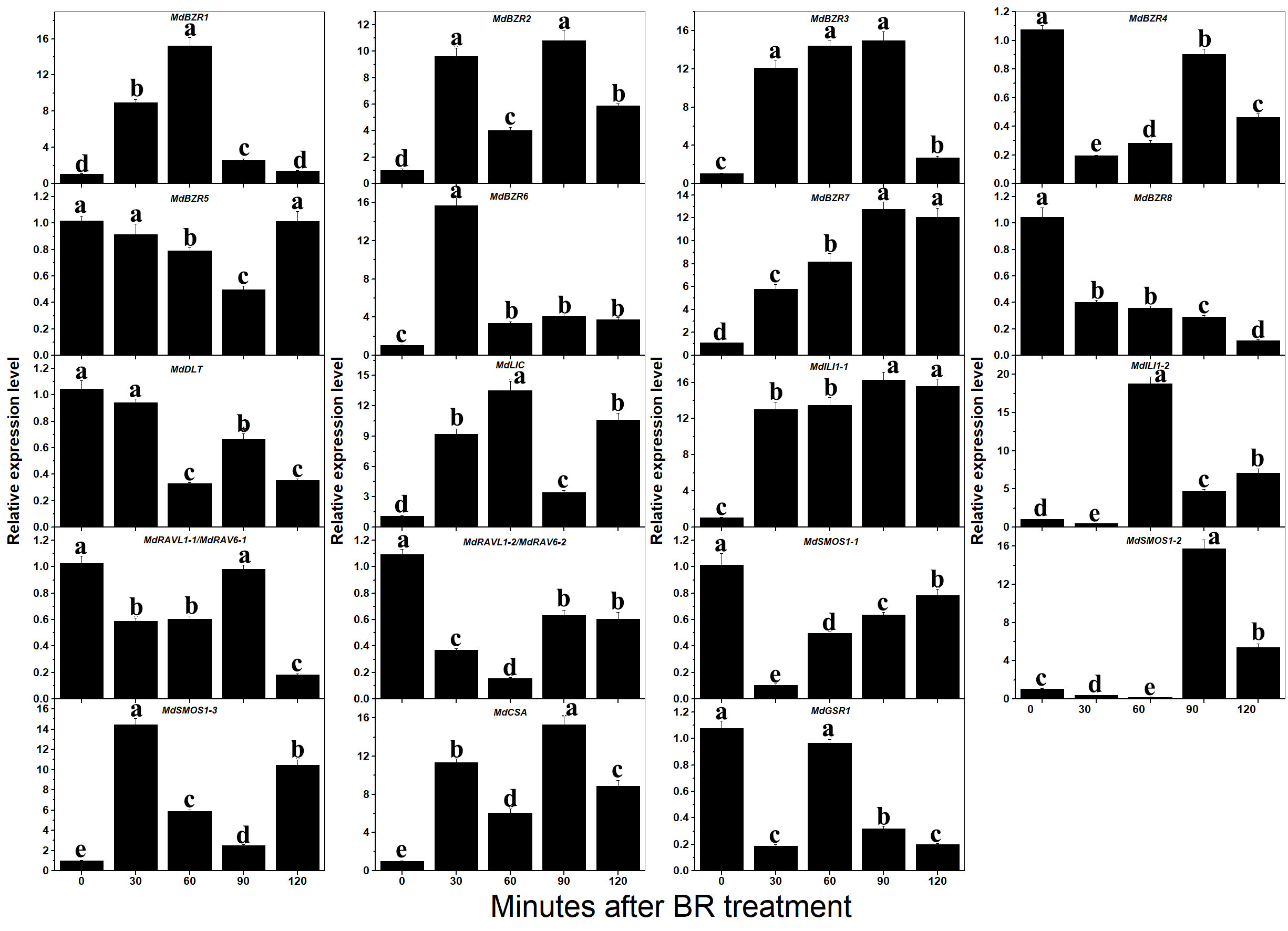

Supplement: Supplementary Figure 10 — Response of BR downstream genes to exogenous BR treatment over 2 h. Each value represents mean ± standard error of three biological replicates. Means followed by small letters are significantly different at 0.05 level (∗). [file Image_3.PNG]

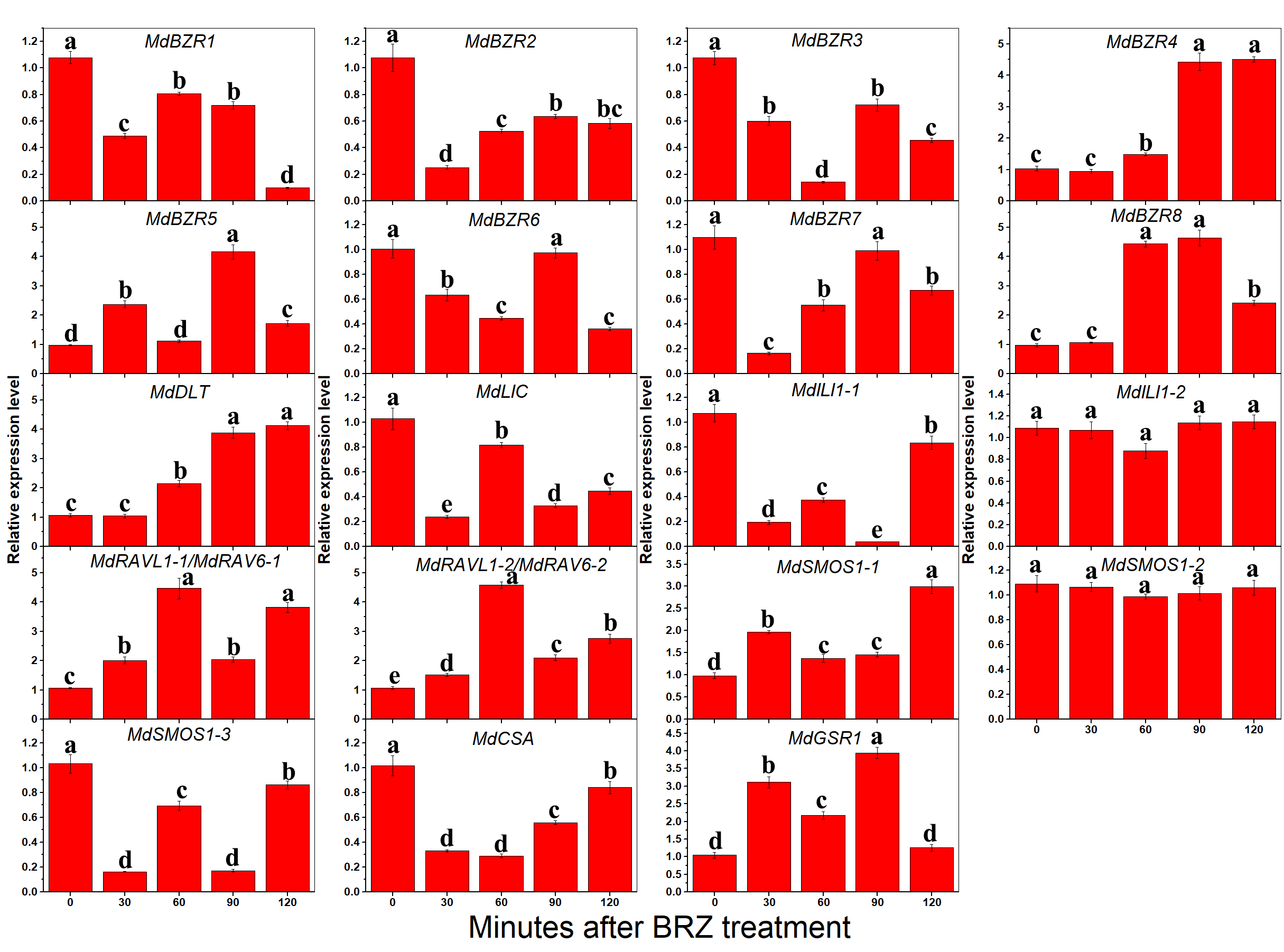

Supplement: Supplementary Figure 11 — Response of BR downstream genes to exogenous BRZ treatment over 2 h. Each value represents mean ± standard error of three biological replicates. Means followed by small letters are significantly different at 0.05 level (∗). [file Image_4.PNG]

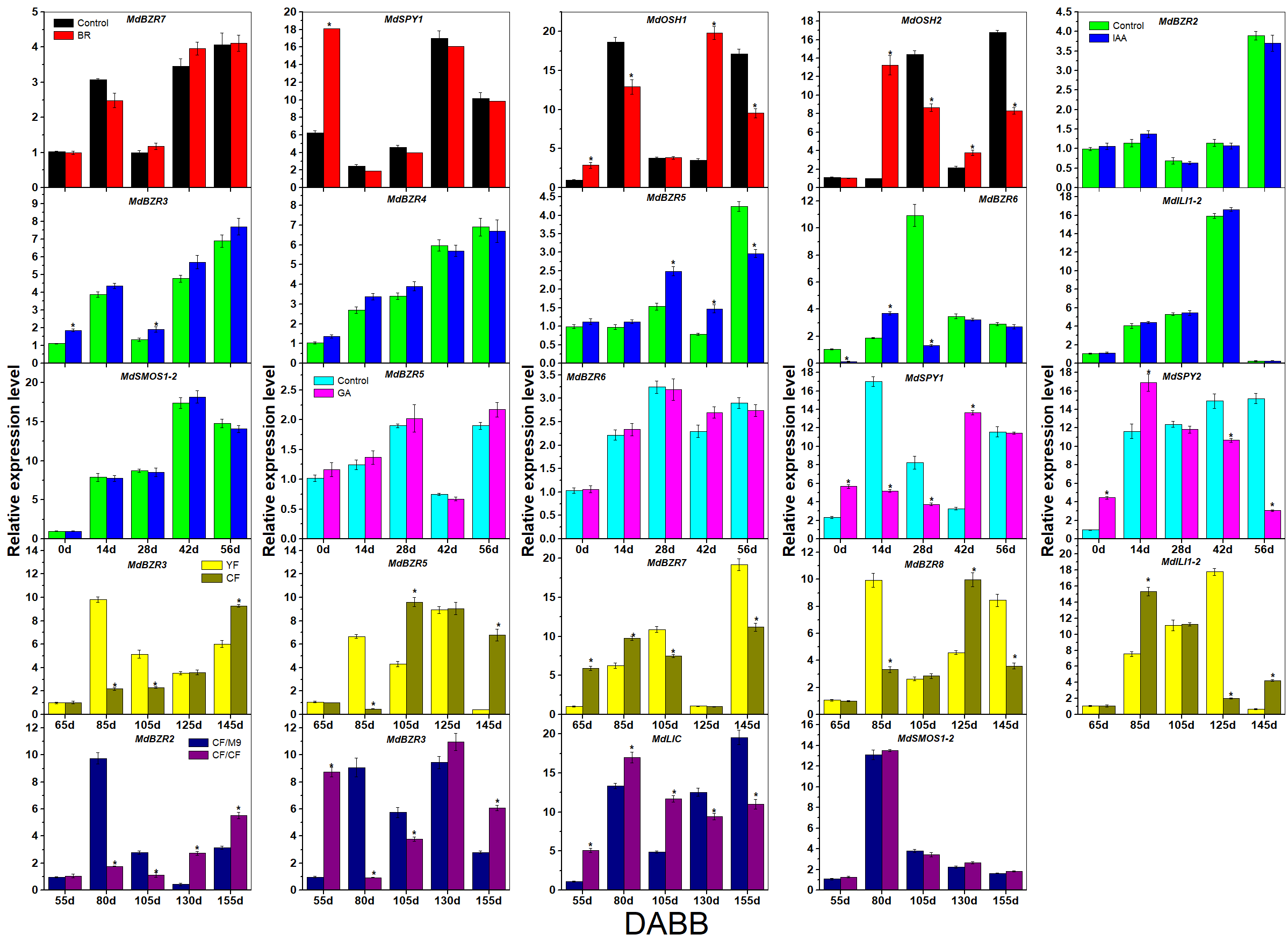

Supplement: Supplementary Figure 12 — Irregular expression patterns of BR downstream genes in response to different treatments. Each value represents mean ± standard error of three biological replicates. ∗ indicates significant differences at 0.05. [file Image_5.PNG]

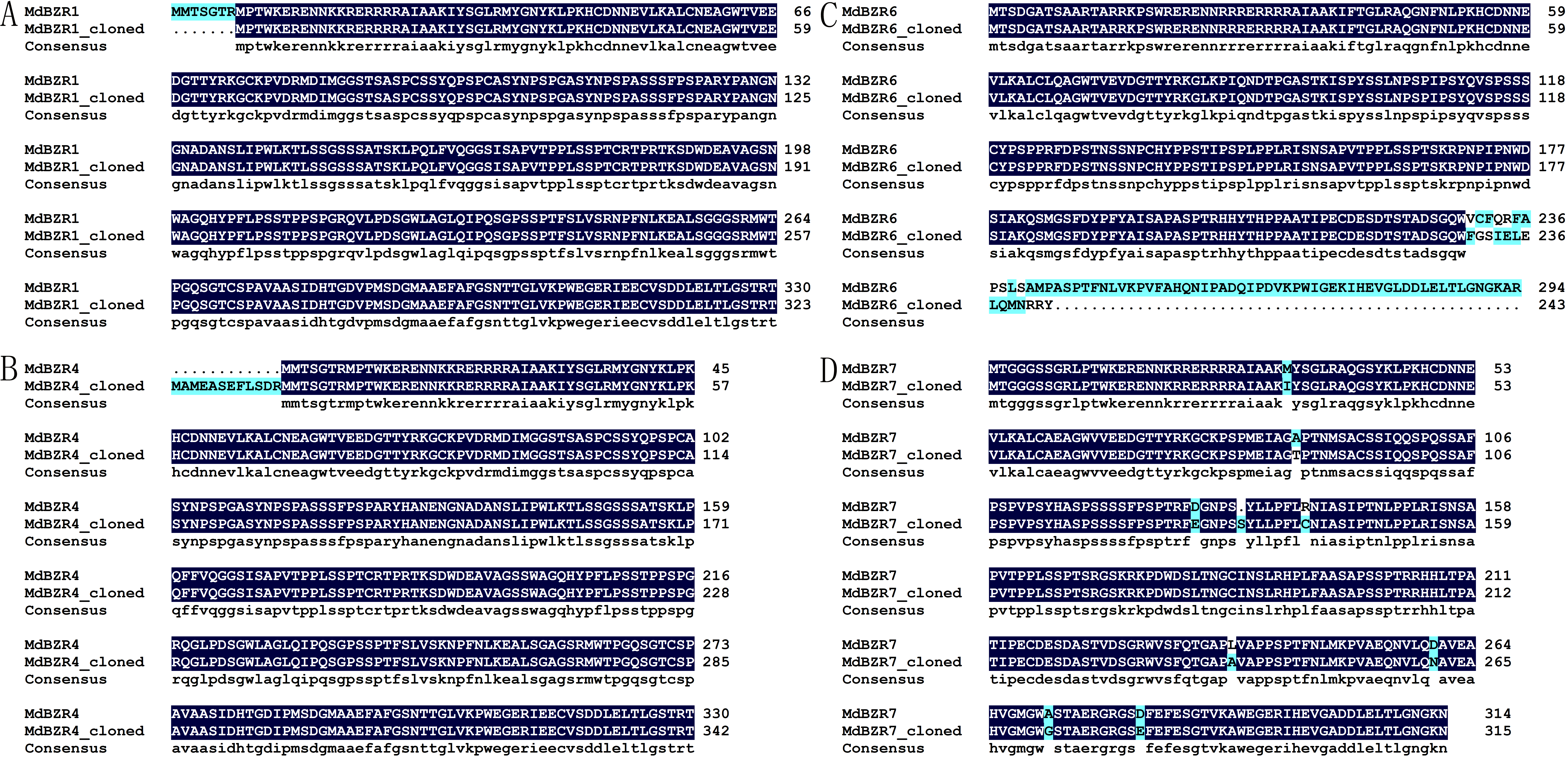

Supplement: Supplementary Figure 14 — Protein sequence alignment between cloned and reference MdBZR genes. (A) MdBZR1 protein; (B) MdBZR4 protein; (C) MdBZR6 protein; (D) MdBZR7 protein. [file Image_7.PNG]
